# Provision of knee bracing for knee osteoarthritis (PROP OA): multicentre, parallel group, superiority, statistician blinded, randomised controlled trial 

**DOI:** 10.1136/bmj-2025-086005

**Published:** 2026-01-26

**Authors:** Melanie A Holden, Elaine Nicholls, Zainab Abdali, Fraser Birrell, Belinda Borrelli, Michael Callaghan, Krysia Dziedzic, David Felson, Nadine E Foster, Nicola Halliday, Carol Ingram, Clare Jinks, Sue Jowett, George Peat, PROP OA trial team, Evans A Asamane, Rachel Browell, Sarah Bathers, Katherine Dobb, Tina Hadley-Barrows, Liz Hartshorne, Dan Herron, Lucy Huckfield, Katrina Humphreys, Jesse Kigozi, Sarah Lawton, Christian Mallen, Michelle Marshall, John McBeth, Gail Sowden, Martin J Thomas

**Affiliations:** 1 School of Medicine, Primary Care Centre Arthritis UK, Keele University, Keele ST5 5BG, UK; 2 Keele Clinical Trials Unit, Keele University, Keele, UK; 3 Health Economics Unit, Department of Applied Health Sciences, University of Birmingham, Birmingham, UK; 4 Medical Research Council Versus Arthritis Centre for Integrated Research into Musculoskeletal Ageing, Population, and Health Sciences Institute, Newcastle University, Newcastle upon Tyne, UK; 5 Northumbria Healthcare NHS Foundation Trust, North Shields, UK; 6 Faculty of Biology, Medicine, and Health, University of Manchester, Manchester, UK; 7 Henry M Goldman School of Dental Medicine, Boston University, Boston, MA, USA; 8 School of Health Sciences, Division of Psychology and Mental Health, Manchester Centre for Health Psychology and Manchester Academic Health Science Centre, University of Manchester, Manchester, UK; 9 Faculty of Health and Education, Manchester Metropolitan University, Manchester, UK; 10 NIHR Manchester Biomedical Research Centre, Manchester University Foundation NHS Trust, Manchester, UK; 11 Impact Accelerator Unit, Keele University, Keele, UK; 12 Research in OsteoArthritis Manchester (ROAM), Centre for Epidemiology Versus Arthritis, Centre for Musculoskeletal Research, Institute of Inflammation and Repair, University of Manchester, Manchester, UK; 13 School of Medicine, Boston University, Boston, MA, USA; 14 STARS Education and Research Alliance, Surgical Treatment and Rehabilitation Service, University of Queensland and Metro North Health, Brisbane, QLD, Australia; 15 Public representative, Research User Group, Primary Care Centre Arthritis UK, School of Medicine, Keele University, Keele, UK; 16 Centre for Applied Health and Social Care Research (CARe), School of Health and Social Care, Sheffield Hallam University, Sheffield, UK

## Abstract

**Objective:**

To determine whether adding compartment specific knee bracing with an adherence intervention to advice, written information, and exercise instruction (AIE+B) is superior to advice, written information, and exercise instruction (AIE) only in improving patient reported outcomes in people with knee osteoarthritis.

**Design:**

Multicentre, parallel group, superiority, statistician blinded, randomised (1:1; block; stratified; centralised web based) controlled trial.

**Setting:**

Recruitment through general practices and the community in Cheshire, Manchester, North Tyneside, and Staffordshire, England; enrolment 25 November 2019 to 16 September 2022.

**Participants:**

466 adults aged ≥45 years with symptoms of knee osteoarthritis.

**Interventions:**

AIE was delivered in one in-person consultation by a trained physiotherapist. Individuals randomised to the AIE+B group were also fitted with a patellofemoral, tibiofemoral unloading, or neutral stabilising knee brace, according to their predominant compartmental distribution of knee osteoarthritis, and were offered a two week follow-up consultation. Brief motivational interviewing with targeted text reminders supported brace adherence.

**Main outcome measures:**

The primary outcome was a composite patient reported Knee Osteoarthritis Outcomes Score (KOOS)-5 (0-100) at six months after randomisation. Key secondary outcomes were KOOS-5 at three and 12 months, KOOS-5 subscale scores, and pain on weight bearing activity at three, six, and 12 months.

**Results:**

466 participants (mean age 64 (standard deviation 9) years; 46% female participants) were randomised, with 401 (86%), 394 (85%), and 370 (79%) participants followed up with analysable data at three, six, and 12 months, respectively. At six months, greater improvement in KOOS-5 was seen in the AIE+B group than in the AIE group (adjusted mean difference 3.39, 95% confidence interval (CI) 0.96 to 5.82; effect size 0.24). Secondary outcomes showed the benefits of AIE+B over AIE that diminished over time. The largest effects observed were for pain reduction (KOOS pain (0-100) adjusted mean difference at six months 6.13, 95% CI 3.36 to 8.91; effect size 0.39). Adverse events were minor and expected.

**Conclusions:**

Adding compartment specific knee bracing and an adherence intervention to advice, written information, and exercise instruction resulted in small improvements in patient reported outcomes among individuals with knee osteoarthritis. This safe intervention offers a potential treatment option for this common condition.

**Trial registration:**

ISRCTN28555470.

## Introduction

Painful knee osteoarthritis affects about 365 million adults worldwide, posing a major and growing challenge to population health, health systems, and economies.^1^ Although primary knee arthroplasty is a highly cost effective intervention, more effective preventive and non-surgical interventions are needed earlier in the care pathways and delivered at scale. Management currently relies on the core strategies of education and self-management advice, exercise, and weight management, with topical and oral analgesia as needed.^2^ ^3^ Knee bracing has been considered as an adjunct, but its value is unclear.

Internationally, clinical guidelines offer conflicting recommendations on the use of knee bracing for knee osteoarthritis,^4^ and evidence from high quality randomised controlled trials is sparse.^5^ The Provision of Braces for Patients with Knee Osteoarthritis (PROP OA) trial was designed to answer the need for a large, independent, high quality randomised controlled trial with outcomes for >6 months. Our primary aim was to determine if compartment specific knee bracing with an adherence intervention as well as advice, written information, and exercise instruction (AIE+B) was superior to advice, written information, and exercise instruction (AIE) only, in adults with symptoms of knee osteoarthritis, in terms of Knee Osteoarthritis Outcomes Score (KOOS)-5, a composite score of patient reported pain, other symptoms, activities of daily living, function in sport or recreation, and knee related quality of life,^6^ at six months. We also wanted to evaluate the effects on overall KOOS-5 at three and 12 months, and the effects on KOOS-5 subscales and several secondary outcomes at three, six, and 12 months. A cost effectiveness evaluation, nested qualitative studies, and comparison between objective and self-reported brace adherence data will be reported separately.

## Methods

### Study design

PROP OA was a multicentre, randomised (1:1), parallel group superiority trial. The trial included an internal pilot phase that assessed the feasibility of progressing to the full trial (supplementary appendix eTable 1). Participants were recruited from general practice consulters and community advertisements from four regions in England (Cheshire, Manchester, North Tyneside, and Staffordshire), with treatment delivered in four NHS Trusts. The study protocol was published by open access,^7^ and the final protocol with a list of amendments is available from the ISRCTN registry. We made minor changes to the protocol because of the covid-19 pandemic, including 10 months of paused recruitment and in-person research activity in response to three national lockdowns, switching from postal to online or telephone supported follow-up questionnaires, and changing from in-person to a mixture of in-person and online physiotherapist training. Also, social participation (from Patient Reported Outcomes Measurement Information System, PROMIS) was an intended secondary outcome, but was erroneously not included in any follow-up questionnaires, and therefore cannot be reported as a secondary outcome.

### Participants

Participants had symptoms of knee osteoarthritis diagnosed after clinical assessment.^8^ Inclusion criteria were participants aged ≥45 years, with moderate-to-severe pain during weight bearing activity (numerical rating scale ≥4), with or without knee buckling, residing in England and Wales, who could undergo knee radiography, who could read and write English, who had access to a mobile phone that could receive SMS messages, who could give full informed consent, and who were willing to participate in the study. Key exclusion criteria were potential serious underlying pathology (eg, cancer or joint infection), inflammatory arthritis (eg, rheumatoid arthritis or psoriatic arthritis), symptoms not attributable to osteoarthritis, previous major knee surgery, on the waiting list for total joint replacement in the next six months, unable or unwilling to wear a knee brace, using a knee brace, course of physiotherapy, or knee injection in the past three months (supplementary appendix eTable 2).^7^

Participants were identified by screening of electronic general practice records to identify adults aged ≥45 years who had consulted for knee pain in the past 24 months and self-referrals from a community awareness raising campaign. Our protocol also allowed for recruitment by physiotherapy services and prospective general practice consultations, but these were not required. After receiving a trial invitation pack, the eligibility of participants was confirmed by initial screening by telephone interview with a trained administrator. Potentially eligible participants were invited to an in-person appointment with a trained physiotherapist who confirmed eligibility and determined, on clinical grounds, the predominant compartmental distribution of knee osteoarthritis (medial tibiofemoral, lateral tibiofemoral, patellofemoral, or no predominant compartment; supplementary appendix eTable 3). When suitable x ray images were not available from the previous 24 months, new plain knee radiographs (weight bearing anteroposterior or posteroanterior, skyline, and lateral views) were taken and reported by the radiology department at the site, based on standard NHS protocols (to rule out possible serious underlying pathology). Eligible participants were invited to return to clinic two weeks later where the physiotherapist assessed the x ray images to verify the predominant compartmental distribution of knee osteoarthritis (supplementary appendix eTable 3). The physiotherapist obtained written informed consent from participants before collection of self-reported baseline data (including sex, defined as female, male, or other), randomisation, and delivery of the interventions.

### Randomisation and masking

We used a computerised web based randomisation service and random number generator, stratified by clinic site, predominant compartmental distribution of knee osteoarthritis (based on a combination of clinical assessment and radiographic presentation), and the presence of instability (buckling), with a 1:1 allocation with random permuted blocks of sizes 2, 4, and 6. Randomisation was executed in real time by a clinic administrator. The randomisation schedule was protected by a password to conceal allocation. Although masking participants or physiotherapists to treatment allocation was not possible, the trial statistician was masked to treatment allocation.

### Procedures

Trial interventions were delivered by physiotherapists who completed a standardised 21 hour training package. This training was delivered face to face or, in response to covid-19 restrictions, by a mixed approach of prerecorded lectures, live virtual sessions, and in-person practical sessions. The training covered the rationale for the trial, processes, and delivery of the interventions (supplementary appendix eTable 4). The content of AIE+B was informed by the existing literature, patient and public involvement representatives, a multidisciplinary clinical advisory group, and current NHS bracing services. The supplementary appendix has full details of the trial interventions, which are summarised below.

#### Comparator: advice, written information, and exercise instruction (AIE) only

Participants assigned to AIE were offered one in-person, 20 minute consultation with a physiotherapist. In line with UK guidance,^8^ this consultation included verbal education and advice about the pathogenesis and prognosis of knee osteoarthritis, the benefits of exercise, increasing physical activity, and weight loss, simple self-help strategies for pain management, written information, and advice and instruction to complete a home based lower limb exercise programme. The exercise programme focused on muscle strengthening, knee range of movement, and proprioception, informed by our previous knee osteoarthritis exercise trial.^9^ This care reflects the routine non-pharmacological care commonly provided in NHS general practice, where knee osteoarthritis is typically managed.^2^ ^3^

#### Intervention: knee bracing as well as advice, written information, and exercise instruction (AIE+B)

Participants assigned to AIE+B were offered an initial one hour in-person treatment session with a physiotherapist, a 30 minute in-person follow-up consultation two weeks later, and motivational prompts by SMS text message to enhance adherence to the brace, tapering in frequency over six months. The initial treatment session included AIE, in line with the comparator arm. Participants were then given a patellofemoral (Bioskin Q Brace), tibiofemoral unloading (first choice brace was Össur Unloader One and second choice brace was Donjoy Nano), or a neutral stabilising knee brace (Össur Formfit Knee Hinged). Braces were selected according to the participant’s predominant compartmental distribution of knee osteoarthritis based on the combined findings of clinical assessment and radiographic presentation. Current and desired level of physical activity, ability to put on and take off the brace, willingness to wear the brace type, and immediate symptom response when the brace was tried on in clinic were also considered. Braces were fitted to ensure maximum comfort. The dose was individually tailored, with participants advised to wear the brace on painful weight bearing activity initially for a minimum of one hour on ≥2 days/week, gradually increasing based on tolerance up to a maximum of 8-12 hours/day. Individuals were advised to wear the brace for six months and to continue wearing it beyond this time if beneficial. Verbal and written information was provided on application and care of the brace, including what to do in the event of slippage, discomfort, or skin irritation. 

Supporting participant material (eg, written information and short video clips) on applying the brace, produced by the manufacturers of the braces, was also made available. Physiotherapists were trained to use brief motivational interviewing techniques to build participants’ intrinsic motivation and resolve ambivalence about adhering to brace use.^10^ The techniques were based on brief strategies to enhance motivation to change, including both communication strategies and motivational techniques. To support self-monitoring and thus enhance adherence, participants were provided with a diary to record their daily use of the brace, barriers to using the brace, and possible solutions to those barriers.

During the follow-up consultation at two weeks, the physiotherapist checked the response to, and fit of, the brace, with the options of recommending increased use, readjustment, and temporary reduction in use, or change in brace if not tolerated and alternative solutions had been explored. Adherence to the use of the brace was reviewed and addressed by using brief motivational interviewing techniques and based on information provided in the brace diary.

Automated motivational prompts to encourage adherence to the brace were sent by SMS text message weekly for the first four weeks, every two weeks for the next eight weeks, and then monthly until the intervention period ended at six months. Content was tailored to the participant’s reported level of use of the brace (low, moderate, or high), based on the number of days they wore the brace in the past week and the average number of hours worn each day.

#### Co-interventions

Participants in both arms could continue to access usual healthcare, including drug treatments and consultations with other health professionals. Participants allocated to AIE were asked not to wear a knee brace for six months after randomisation. Details of co-interventions were recorded in follow-up questionnaires.

### Outcomes

Participants were followed up by self-report questionnaires at three, six, and 12 months after randomisation. To enhance responses, participants received reminders, minimal data collection at the primary end point (six months), and a £10 (€11.5; US$13.4) gift voucher with each follow-up questionnaire.

The primary outcome was KOOS-5 at six months (0-100).^6^ Key secondary outcomes included KOOS-5 at three and 12 months, and KOOS subscale scores (0-100) (pain, other symptoms, activities of daily living, function in sport or recreation, and knee related quality of life) and knee pain on weight bearing activity (0-10) at three, six, and 12 months. Other secondary outcomes were KOOS-4,^11^ Western Ontario and McMaster Universities Osteoarthritis Index (WOMAC) pain, stiffness, and function,^12^ the Intermittent and Constant Osteoarthritis Pain Scale,^13^ instability or buckling,^14^ OMERACT-OARSI (Outcome Measures in Rheumatology-Osteoarthritis Research Society International) responder criteria,^15^ ^16^ score on the International Physical Activity Questionnaire for the Elderly^17^ and Arthritis Self-Efficacy Scale,^18^ treatment acceptability (measured by self-reported items aligned with the theoretical framework of acceptability),^19^ and intervention adherence (self-report from a Likert scale) at three, six, and 12 months. Adherence to the use of the brace (brace wear time) was also captured in those allocated to AIE+B by questionnaires and SMS text messaging. Treatment fidelity was assessed with case report forms that physiotherapists completed at every treatment session to record the care provided. Case report forms were also used to record physiotherapists’ confidence in judging the predominant compartmental distribution of knee osteoarthritis by clinical assessment only, radiographic presentation only, and in combination. Adverse events were monitored and recorded from case report forms, contact with the trial team, physiotherapist reports, and questionnaires at each follow-up time point.

### Sample size

The trial was powered to detect an effect size between groups of 0.35 (small-to-medium effect) in KOOS-5 at six months with two sided 5% significance and 90% power. Assuming a standard deviation of 23,^9^ this value equated to a minimum clinically important difference of eight points on the KOOS-5, which aligns with published evidence for the tool.^20^ We aimed to randomise 434 participants to allow for 20% of participants lost to follow-up at six months (target of n=346 at six months; 173 participants in each arm).

### Statistical analysis

A full analysis plan was specified before the last patient follow-up and data lock, and was approved by the independent trial steering committee and data monitoring committee. The data analysis plan is provided in the online supplementary material and is publicly available on the ISRCTN trial register. An outline of the data analysis plan is given here.

Longitudinal mixed models were used to estimate treatment effects for primary and secondary outcomes at the three, six, and, 12 month follow-up periods, with results presented as adjusted mean differences and effect sizes, or adjusted odds ratios (AIE+B *v* AIE) for continuous and categorical outcomes, respectively, with 95% confidence intervals (CIs). We used longitudinal mixed models rather than analysis of covariance on imputed data because our multiple imputation model that included all primary and secondary outcomes failed to converge (this approach aligns with our prespecified analysis plan). The choice of covariates for adjustment followed recommendations to include factors used to stratify randomisation,^21^ adjust for the continuous baseline score of the primary outcome measure,^21^ and use a parsimonious set of covariates known to be prognostic of symptom progression in osteoarthritis trials.^22^ Adjusted covariates were: baseline value of the outcome of interest (if applicable for the modelled outcome); clinic site; predominant knee osteoarthritis compartment involved; presence or absence of instability (buckling) (ie, the randomisation strata); age; sex; and anxiety or depression. Primary and secondary analyses were conducted using a treatment policy estimand^23^ (equivalent to intention to treat), excluding outcome data collected after knee replacement. Safety was analysed for all randomised participants. Multiple imputation (focused around estimating the primary outcome only) was used to explore the sensitivity of the trial findings to the assumption that data were missing at random (controlled imputation^24^), estimate the treatment effect in participants treated according to the trial protocol, and explore the potential role of the covid-19 pandemic on the findings of the trial. We prespecified the covid-19 analysis to acknowledge that participants were recruited into the trial before, during, and after the covid-19 pandemic and to examine the impact of the pandemic on our treatment effect of interest. We investigated this impact by (temporarily) deleting any data from the dataset that could potentially be affected by the covid-19 pandemic and used multiple imputation to then estimate what the outcome measure would have been if it had not been collected during the covid-19 pandemic.^25^ The treatment effect was then re-estimated on the imputed data.

Treatment adherence, along with self-reported reasons for non-adherence, were reported with descriptive statistics. Structural equation modelling (latent mixture modelling) was also used to estimate complier average causal effects for two a priori definitions of treatment adherence (ie, participants were adherent if they reported wearing the brace for at least one hour on ≥2 days in the past seven days at either the three or six month follow-up period (definition 1); or the same criteria were met at both the three and six month follow-up times (definition 2)). Our aim was to estimate the treatment effect in participants adhering to AIE+B by comparing the difference in KOOS-5 at six months between participants randomised to AIE+B who adhered to the use of the brace and participants randomised to AIE who would have (hypothetically) adhered to the use of the brace if they had been allocated to AIE+B.^26^ ^27^

Exploratory subgroup analyses for predominant compartmental distribution of knee osteoarthritis, knee buckling, adherence, sex, anxiety, depression, and baseline KOOS-5 were performed by adding an interaction term to the mixed models in the primary analysis. The impact of correlation in outcomes caused by each physiotherapist delivering the intervention to multiple participants was investigated by adding a random effect term to the primary treatment model to account for this variation in the data. Per cent agreement and an unweighted, unadjusted κ statistic were used to determine whether, and to what extent, clinical judgment on the predominant compartmental distribution of knee osteoarthritis and brace allocation changed based on radiographic findings; numbers and percentages were also used to describe physiotherapists’ confidence in determining this assessment. Treatment delivery, acceptability, and fidelity, missing data rates, recruitment flow, and participant characteristics were described with descriptive statistics. No interim analysis was undertaken to assess clinical effectiveness. Data were analysed with Stata version 18.0.^28^

### Patient and public involvement

Patient and public involvement were embedded throughout the study. All patient and public involvement activities were supported by a patient and public involvement support worker and reimbursed in line with National Institute of Health and Care Research (NIHR) guidance (supplementary appendix).

## Results

**Fig 1:**
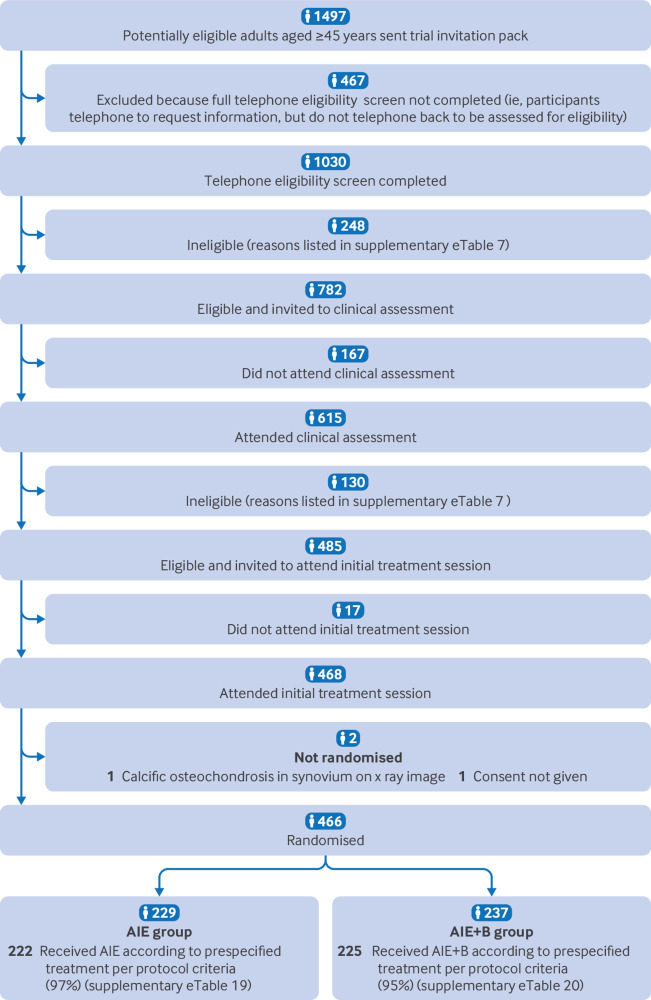
Flowchart of study population selection. AIE=advice, written information, and exercise instruction; AIE+B=advice, written information, and exercise instruction with knee bracing

**Fig 2:**
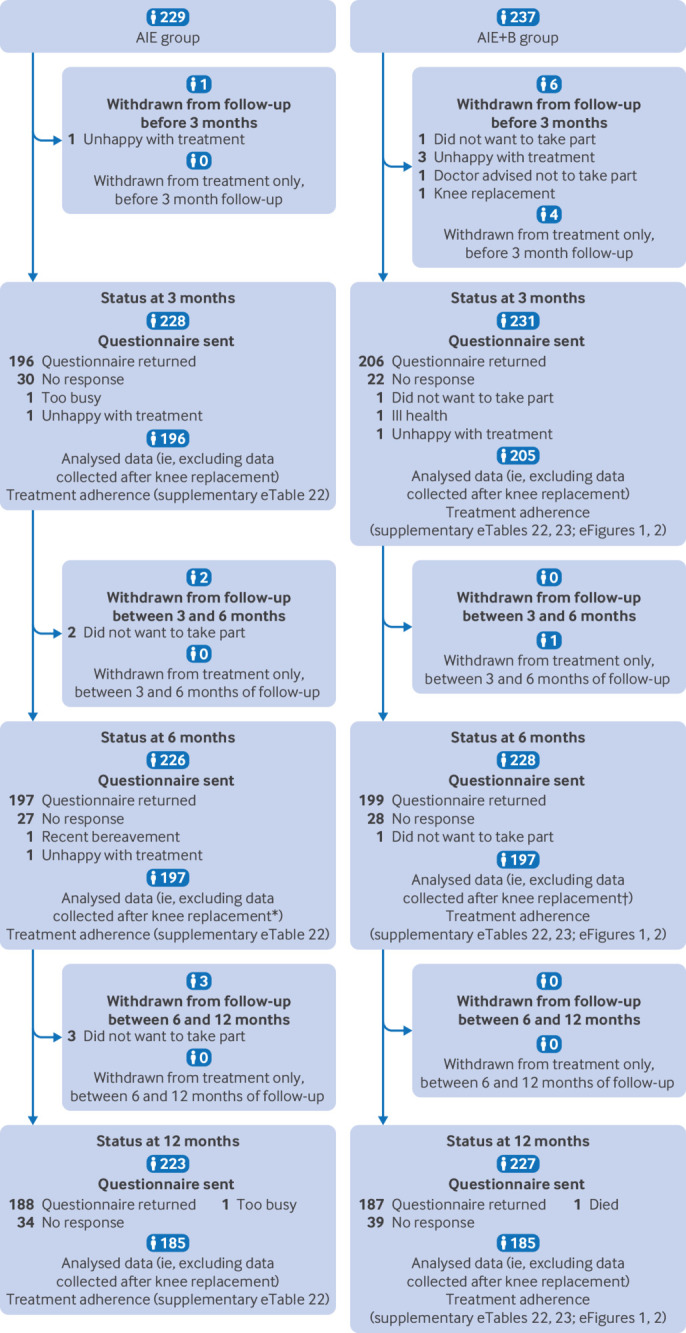
Trial profile after randomisation up to 12 month follow-up period. *Of 197 questionnaires analysed at six month follow-up, 12 were from minimum data collection (ie, a shorter version of the full questionnaire). †Of 197 questionnaires analysed at six month follow-up, 12 were from minimum data collection (ie, a shorter version of the full questionnaire). AIE=advice, written information, and exercise instruction; AIE+B=advice, written information, and exercise instruction with knee bracing

Between 25 November 2019 and 16 September 2022, 1497 potential participants were issued a trial invitation pack, 1030 and 615 participants underwent telephone and in-person screening, respectively, and 466 participants were eligible and randomised to AIE (n=229) or AIE+B (n=237) (fig 1, fig 2, and supplementary appendix eTable 6). The number of participants enrolled was above the target sample size because some participants had already met the telephone screening inclusion criteria when the recruitment target had been achieved. These participants therefore continued with the trial recruitment processes and were randomised if eligible. Predominant reasons for ineligibility at the telephone screening were knee pain severity <4 (n=65, 26%) and recent physiotherapy (n=35, 14%). Predominant reasons for ineligibility at the in-person screening were knee brace contraindicated (n=37, 28%) and knee pain not attributable to osteoarthritis (n=33, 25%) (supplementary appendix eTable 7). Among trial participants, 153 (33%) had osteoarthritis predominantly in the medial tibiofemoral compartment, 21 (5%) in the lateral tibiofemoral compartment, 101 (22%) in the patellofemoral compartment, and 191 (41%) showed no clear predominant compartmental involvement, based on clinical assessment and radiographic presentation. Both arms had similar characteristics at baseline (table 1, table 2, and supplementary appendix eTables 8 and 9).

**Table 1:** Baseline characteristics: personal and general health and wellbeing characteristics, and radiographic osteoarthritis severity in all participants, and AIE group (advice, written information, and exercise instruction) and AIE+B group (advice, written information, and exercise instruction with knee bracing)

	All randomised participants (n=466)*	AIE group (n=229)*	AIE+B group (n=237)*
Mean (SD) age (years)	64 (9)	64 (9)	64 (9)
Female participants	213 (46)	113 (49)	100 (42)
Ethnic group:			
White	449 (97)	221 (97)	228 (96)
Black Caribbean	0 (0)	0 (0)	0 (0)
Black African	1 (0)	1 (0)	0 (0)
Black other	2 (0)	0 (0)	2 (1)
Indian	4 (1)	1 (0)	3 (1)
Pakistani	2 (0)	1 (0)	1 (0)
Bangladeshi	0 (0)	0 (0)	0 (0)
Chinese	3 (1)	2 (1)	1 (0)
Prefer not to say	3 (1)	1 (0)	2 (1)
Other, stated as North African	1 (0)	1 (0)	0 (0)
Left school to attend full time education or university	195 (42)	86 (38)	109 (46)
Currently in paid employment (full or part time)	193 (42)	92 (41)	101 (43)
Index of multiple deprivation (1-32 844):			
Mean (SD)	19 676 (9006)	19 622 (9182)	19 727 (8851)
Median (IQR)	21 376 (12 178-27 107)	21 395 (12 292-27 571)	21 356 (12 034-26 829)
Index of multiple deprivation group:			
Group 1 (most deprived): 1-6568	56 (12)	28 (12)	28 (12)
Group 2: 6569-13137	67 (14)	31 (14)	36 (15)
Group 3: 13 138-19 706	80 (17)	43 (19)	37 (16)
Group 4: 19 707-26 275	126 (27)	56 (24)	70 (30)
Group 5 (least deprived): 26 276-32 844	137 (29)	71 (31)	66 (28)
Pain in the past 4 weeks lasting ≥1 day in any part of the body:	381 (86)	186 (85)	195 (87)
Bilateral knee pain†	161 (35)	83 (36)	78 (33)
Upper limb pain†	173 (37)	86 (38)	87 (37)
Lower limb pain, excluding the knee†	312 (67)	158 (69)	154 (65)
Manchester definition of widespread pain†	58 (12)	27 (12)	31 (13)
Long term (>12 months) physical or mental health condition, disability, or illness:	210 (46)	101 (45)	109 (47)
Blindness or partial sight	15 (3)	9 (4)	6 (3)
Breathing condition (eg, asthma or COPD)	54 (12)	22 (10)	32 (14)
Cancer (diagnosis or treatment in past 5 years)	14 (3)	4 (2)	10 (4)
Deafness or hearing loss	64 (14)	28 (12)	36 (15)
Diabetes	37 (8)	13 (6)	24 (10)
Heart condition (eg, angina or atrial fibrillation)	37 (8)	16 (7)	21 (9)
High blood pressure	132 (28)	70 (31)	62 (26)
Kidney or liver disease	10 (2)	3 (1)	7 (3)
Mental health condition	29 (6)	13 (6)	16 (7)
Neurological condition (eg, epilepsy)	7 (2)	4 (2)	3 (1)
Stroke (which affects day-to-day life)	3 (1)	1 (0)	2 (1)
Taking >5 medications on a regular basis	130 (28)	66 (29)	64 (27)
Body mass index:			
Mean (SD)	29.0 (5.6)	29.0 (5.7)	29.0 (5.5)
Median (IQR)	28.0 (24.9-31.7)	28.2 (25.1-31.5)	28.0 (24.8-31.9)
Categorised body mass index:			
Underweight (<18.5)	0 (0)	0 (0)	0 (0)
Normal weight (≥18.5 and <24.9)	112 (25)	53 (24)	59 (26)
Overweight (≥24.9 and <29.9)	168 (37)	85 (38)	83 (37)
Obese (≥29.9)	169 (38)	84 (38)	85 (37)
Hospital Anxiety and Depression Scale: anxiety (0-21):			
Mean (SD)	5.4 (4.0)	5.3 (3.9)	5.6 (4.0)
Median (IQR)	5.0 (2.0-8.0)	5.0 (2.0-8.0)	5.0 (3.0-8.0)
Hospital Anxiety and Depression Scale: depression (0-21):			
Mean (SD)	4.5 (3.1)	4.5 (3.2)	4.5 (3.0)
Median (IQR)	4.0 (2.0-7.0)	4.0 (2.0-7.0)	4.0 (2.0-7.0)
Kellgren-Lawrence highest grade per knee:			
0	16 (3)	7 (3)	9 (4)
1	6 (1)	4 (2)	2 (1)
2	122 (26)	57 (25)	65 (27)
3	215 (46)	107 (47)	108 (46)
4	107 (23)	54 (24)	53 (22)

Values are number (%) unless indicated otherwise.

High score indicates least deprived for index of multiple deprivation, and worse outcome for Hospital Anxiety and Depression Scale and Kellgren-Lawrence score. X ray images were scored for Kellgren-Lawrence grade at the end of the trial and were the highest grade given to a compartment (or compartments if no predominant compartment involvement), so may not directly align with the clinical judgment of the x ray image that was used to guide brace allocation. Intra-rater reliability of the radiographic scoring was high: intraclass correlation coefficient=0.94 (95% confidence interval 0.90 to 0.96), as estimated from a two way random effects model with absolute agreement.

* Baseline questionnaire data missing for one participant, so baseline questionnaire variables are based on 465 participants with data.

† Defined based on the pain regions of the Manchester definition of widespread pain.

COPD= chronic obstructive pulmonary disease; IQR=interquartile range; SD=standard deviation.

**Table 2:** Trial outcome measures (where measured) at baseline in all participants, and in AIE group (advice, written information, and exercise instruction) and AIE+B group (advice, written information, and exercise instruction with knee bracing)


All randomised participants (n=466)*
AIE group (n=229)*
AIE+B group (n=237)*
Mean (SD) KOOS-5 (primary outcome) (0-100)
45.3 (13.9)
44.9 (13.5)
45.7 (14.2)
Mean (SD) KOOS: pain (0-100)
54.2 (15.7)
53.9 (15.2)
54.5 (16.2)
Mean (SD) KOOS: symptoms (0-100)
45.0 (13.4)
44.5 (13.8)
45.5 (13.0)
KOOS: activities of daily living (0-100):



Mean (SD)
60.0 (18.8)
59.8 (18.3)
60.2 (19.4)
Median (IQR)
60.3 (45.6-75.0)
58.8 (45.6-73.5)
60.3 (45.6-76.5)
KOOS: sport or recreation (0-100):



Mean (SD)
32.8 (23.5)
31.6 (21.2)
34.0 (25.6)
Median (IQR)
30.0 (15.0-50.0)
30.0 (15.0-45.0)
30.0 (15.0-50.0)
Mean (SD) KOOS: knee related quality of life (0-100)
33.9 (17.2)
33.8 (16.8)
34.0 (17.6)
Mean (SD) KOOS-4: (0-100)
48.3 (13.3)
48.0 (13.1)
48.6 (13.6)
WOMAC:



Mean (SD) pain (0-20)
8.1 (3.5)
8.1 (3.4)
8.0 (3.6)
Mean (SD) stiffness (0-8)
4.0 (1.5)
4.0 (1.5)
3.9 (1.5)
Function (0-68):



Mean (SD)
27.2 (12.8)
27.4 (12.4)
27.0 (13.2)
Median (IQR)
27.0 (17.0-37.0)
28.0 (18.0-37.0)
27.0 (16.0-37.0)
Mean (SD) knee pain during activity in the knee in past 7 days, (0-10)
6.3 (1.8)
6.4 (1.7)
6.3 (1.8)
ICOAP, constant pain subscale (0-100):



Mean (SD)
36.6 (26.8)
37.6 (26.2)
35.7 (27.4)
Median (IQR)
35.0 (10.0-60.0)
40.0 (15.0-60.0)
35.0 (10.0-55.0)
Mean (SD) intermittent pain subscale (0-100)
49.1 (20.4)
49.9 (20.0)
48.2 (20.8)
Mean (SD) total pain scale (0-100)
43.4 (21.7)
44.3 (21.0)
42.5 (22.5)
Instability (buckling): knee buckled at least once in past 3 months (No (%)):



No or not sure
228 (49)
111 (49)
117 (49)
Yes
237 (51)
117 (51)
120 (51)
Mean (SD) Arthritis Self-Efficacy Scale (1-10)
5.3 (1.9)
5.2 (1.8)
5.4 (2.1)
IPAQ-E, physical activity (MET min/week; 0–19 278):



Mean (SD)
4265 (3262)
4507 (3316)
4032 (3199)
Median (IQR)
3590 (1635-6132)
3706 (1760-6399)
3386 (1515-5718)

All outcome measures completed in reference to the knee to be treated. High score indicates more active for IPAQ-E, worse outcome for WOMAC, knee pain on activity, and ICOAP, and better outcome for KOOS and Arthritis Self-Efficacy Scale.

* Baseline questionnaire data missing for one participant, so baseline questionnaire variables are based on 465 participants with data.

† Defined based on the pain regions of the Manchester definition of widespread pain.

IQR=interquartile range; SD=standard deviation; KOOS=Knee Osteoarthritis Outcomes Score; WOMAC=Western Ontario and McMaster Universities Arthritis Index; ICOAP=Intermittent and Constant Osteoarthritis Pain Scale; IPAQ-E=International Physical Activity Questionnaire for the Elderly; MET=metabolic equivalent of task.

Of the 466 participants randomised, 394 (85%) returned analysable data at six months, and 401 (86%) and 370 (79%) at three and 12 months, respectively. Twelve participants withdrew from trial follow-up (AIE n=6; AIE+B n=6). We found some evidence to suggest that participants lost to follow-up had more severe knee pain than those who completed follow-up (supplementary appendix eTables 10 and 11). Missing data rates for the primary outcome (KOOS-5) were low at all time points (supplementary appendix eTables 12 and 13). Eighty eight minor protocol deviations occurred, mostly in the AIE+B group because of missed SMS prompts for brace adherence (n=59) (supplementary appendix eTable 14).

Eighteen physiotherapists with a median of 10 years of experience (range 1-36 years) undertook PROP OA trial training and delivered both interventions. Agreement between physiotherapists’ clinical judgment of the predominant compartmental distribution of knee osteoarthritis alone and when combined with radiographic presentation was 86% (κ=0.79, 95% CI 0.74 to 0.84) (supplementary appendix eTables 15-17). Physiotherapists seemed more confident in judging the predominant compartmental distribution of knee osteoarthritis when clinical assessment findings were combined with radiographic presentation (very or extremely confident, n=344, 74%), rather than when based on clinical assessment only (very or extremely confident, n=242, 53%) (supplementary appendix eTable 18).

Interventions were delivered according to our prespecified treatment per protocol criteria for 222 (97%) participants in the AIE group and for 225 (95%) participants in the AIE+B group. Although not considered a protocol violation, 91 (40%) participants in the AIE group also received at least one element of motivational interviewing (supplementary appendix eTables 19-21). Thirty seven participants (16%) in the AIE group reported wearing a knee brace (type not specified) in at least one follow-up questionnaire.

Participant self-reported adherence to the trial interventions diminished over time in both arms. In the AIE+B group, 120 (66%) participants met our a priori definition of self-reported brace adherence (minimum time wearing the brace of one hour on ≥2 days/week) at three and six months. Reasons for not wearing the brace varied, but most commonly was because of the brace not fitting under clothing (n=45, 24% at six months) (supplementary appendix eTables 22 and 23, and eFigures 1 and 2).

**Fig 3:**
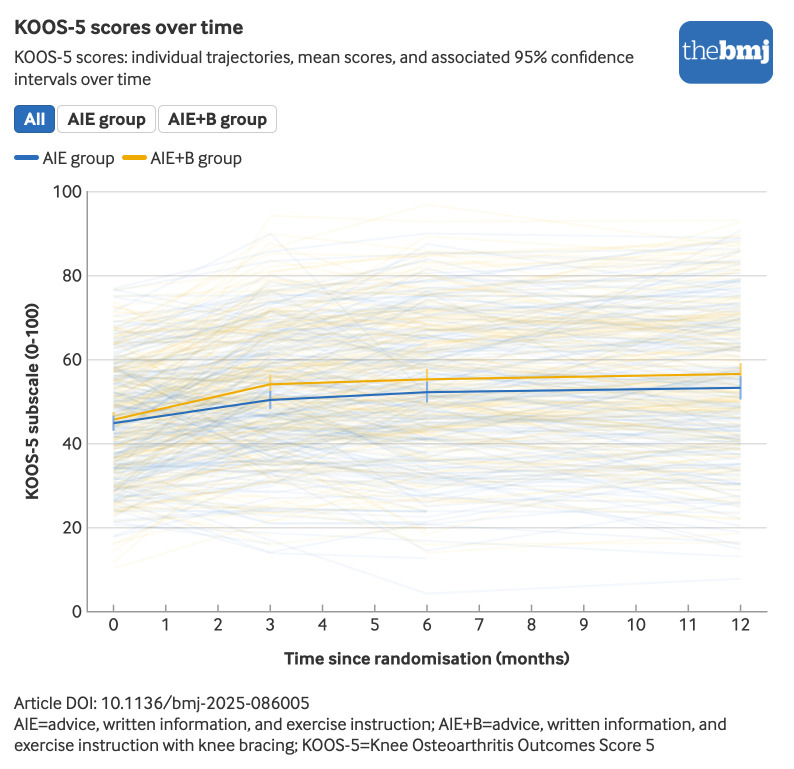
KOOS-5 scores over time. An interactive version of this graphic is available at https://public.flourish.studio/visualisation/26004727/

At six months, we found greater improvement in KOOS-5 in the AIE+B group than in the AIE group (adjusted mean difference 3.39, 95% CI 0.96 to 5.82; effect size 0.24). Results were similar at three months (adjusted mean difference 3.67, 95% CI 1.47 to 5.87; effect size 0.26). At 12 months, although improvement was still greater in the AIE+B group than in the AIE group, this improvement was reduced in magnitude and was no longer significant (adjusted mean difference 2.67, 95% CI −0.24 to 5.57; effect size 0.19) (fig 3, table 3, table 4, and supplementary appendix eTable 24). Sensitivity analyses were largely consistent with the primary analysis (table 3). Although the treatment effect was smaller in analyses assessing the impact of covid-19, this effect was not significant (adjusted mean difference 1.59, 95% CI −1.37 to 4.56).

**Table 3:** Treatment effect estimates for the primary outcome (Knee Osteoarthritis Outcomes Score (KOOS)-5) at six months

KOOS-5	Treatment effect: AIE *v* AIE+B (adjusted mean difference (95% CI))*
Longitudinal mixed models	3.39 (0.96 to 5.82)
Data after multiple imputation of missing data:†	
Treatment effect	3.32 (0.96 to 5.68)
Treatment effect accounting for outcome variation between physiotherapists‡	3.32 (0.73 to 5.90)
Treatment effect investigating impact of data not being missing at random (controlled imputation):	
Scenario 1: data defined as missing if participants have at least one KOOS-5 subscale missing: n=87 (19%):	
Delta=2	3.38 (1.02 to 5.75)
Delta=4	3.44 (1.06 to 5.83)
Delta=6	3.51 (1.10 to 5.91)
Delta=8	3.57 (1.13 to 6.01)
Delta=−2	3.26 (0.90 to 5.61)
Delta=−4	3.19 (0.83 to 5.56)
Delta=−6	3.13 (0.75 to 5.51)
Delta=−8	3.07 (0.66 to 5.48)
Scenario 2: data defined as missing if participants have all KOOS-5 subscales missing: n=72 (15%):	
Delta=2	3.37 (1.01 to 5.74)
Delta=4	3.43 (1.05 to 5.81)
Delta=6	3.48 (1.08 to 5.88)
Delta=8	3.53 (1.10 to 5.97)
Delta=−2	3.26 (0.91 to 5.62)
Delta=−4	3.21 (0.85 to 5.58)
Delta=−6	3.16 (0.78 to 5.53)
Delta=−8	3.10 (0.70 to 5.50)
Treatment effect for the hypothetical scenario that all participants met our a priori definition of treatment delivered per protocol§	3.20 (0.85 to 5.56)
Treatment effect for the hypothetical scenario that all participants were recruited in a world where covid-19 already existed, and where individuals could have covid-19 infections	1.59 (−1.37 to 4.56)
Complete case analysis: complier average causal effect:	
Treatment effect for the principal stratum of participants who adhered to the bracing component of the AIE+B trial arm (adherence definition 1¶)	3.87 (1.06 to 6.69)**
Treatment effect for the principal stratum of participants who adhered to the bracing component of the AIE+B trial arm (adherence definition 2††)	5.21 (1.48 to 8.94)**

Mean KOOS-5 was 52.3 (standard deviation 17.3) in the AIE group and 55.3 (17.0) in the AIE+B group at six months.

* Adjusted for PROP OA (Provision of Braces for Patients with Knee Osteoarthritis) trial clinic site, predominant compartmental distribution based on clinical and radiographic presentation, presence or absence of instability (buckling), age, sex, baseline anxiety, baseline depression, and baseline KOOS-5 score.

† Variables included in the imputation model: KOOS (separate subscales); Intermittent and Constant Osteoarthritis Pain Scale; Arthritis Self-Efficacy Scale; knee pain during activity and International Physical Activity Questionnaire for the Elderly (baseline, and three, six, and 12 month follow-up); knee buckling; anxiety; depression (baseline only); adherence (followed advice and treatment from physiotherapist (three, six, and 12 month follow-up); age; sex; PROP OA clinic site; and predominant compartmental distribution based on clinical and radiographic presentation. Imputed data after knee replacement were excluded from the analysis. For the sensitivity analysis, to explore the impact of the covid-19 pandemic, the imputation model would not converge on this dataset, so the model was simplified by removing the adherence variables, and imputing knee buckling with predictive mean matching rather than logistic regression. Lack of convergence may have occurred because about 50% of KOOS-5 scores at six months were based on imputed data.

‡ Defined as the physiotherapist who delivered the initial treatment session. For participants with a follow-up visit, 159 (70%) were treated by the same physiotherapist who delivered their initial treatment session.

§ AIE was delivered according to the protocol if participants were given verbal advice and education (about osteoarthritis or about things to try at home to help with symptoms), were provided with written information about osteoarthritis (the osteoarthritis guidebook), and were prescribed a knee exercise programme. AIE+B was delivered according to the protocol if participants received AIE according to the protocol, a knee brace, at least one brief motivational interviewing technique, at least one SMS motivational prompt, and a follow-up treatment session (either remotely or face to face).

¶ Participants were defined as adherent to treatment if, at either three or six months, they reported wearing the brace for at least one hour on ≥2 days in the past seven days (based on self-reported questionnaire data).

** See supplementary appendix eTable 27 for complier average causal effect models for the primary outcome at the six month follow-up.

†† Participants were defined as adherent to treatment if, at both three and six months, they reported wearing the brace for at least one hour on ≥2 days in the past seven days (based on self-reported questionnaire data).

AIE=advice, written information, and exercise instruction; AIE+B=advice, written information, and exercise instruction with knee bracing; CI=confidence interval; SMS= short message service.

**Table 4:** Treatment effect estimates for primary and secondary outcome measures at all time points

Outcome measure*†	3 months (n=401)	6 months (n=394)	12 months (n=370)
KOOS-5 (0-100):			
AIE: mean (SD)	50.4 (15.1)	52.3 (17.3)	53.3 (18.6)
AIE+B: mean (SD)	54.1 (15.8)	55.3 (17.0)	56.6 (17.4)
AIE *v* AIE+B: adjusted mean difference (95% CI)‡§	3.67 (1.47 to 5.87)	3.39 (0.96 to 5.82)	2.67 (−0.24 to 5.57)
KOOS: pain (0-100):			
AIE: mean (SD)	58.3 (18.0)	58.7 (19.1)	60.6 (20.5)
AIE+B: mean (SD)	62.8 (16.8)	64.4 (18.1)	65.5 (18.4)
AIE *v* AIE+B: adjusted mean difference (95% CI)‡§	4.30 (1.71 to 6.89)	6.13 (3.36 to 8.91)	4.76 (1.48 to 8.04)
KOOS: symptoms (0-100):			
AIE: mean (SD)	48.3 (13.2)	49.7 (14.7)	49.6 (13.8)
AIE+B: mean (SD)	52.0 (13.3)	52.3 (13.6)	52.7 (13.2)
AIE *v* AIE+B: adjusted mean difference (95% CI)‡§	2.97 (0.95 to 4.98)	2.15 (−0.08 to 4.39)	2.01 (−0.19 to 4.20)
KOOS: activities of daily living (0-100):			
AIE: mean (SD)	64.8 (19.2)	65.1 (21.2)	67.1 (21.4)
AIE+B: mean (SD)	68.6 (19.1)	69.8 (19.4)	70.3 (20.0)
AIE *v* AIE+B: adjusted mean difference (95% CI)‡§	4.12 (1.55 to 6.69)	5.24 (2.47 to 8.02)	3.60 (0.30 to 6.89)
KOOS: sport or recreation (0-100):			
AIE: mean (SD)	38.9 (23.7)	42.5 (26.9)	43.7 (28.0)
AIE+B: mean (SD)	42.6 (26.6)	44.3 (27.9)	45.6 (29.0)
AIE *v* AIE+B: adjusted mean difference (95% CI)‡§	3.32 (−0.75 to 7.38)	1.09 (−3.34 to 5.53)	0.30 (−4.70 to 5.29)
KOOS: knee related quality of life (0-100):			
AIE: mean (SD)	41.0 (18.3)	43.4 (20.4)	45.3 (21.8)
AIE+B: mean (SD)	43.8 (19.6)	45.5 (20.2)	47.7 (20.9)
AIE *v* AIE+B: adjusted mean difference (95% CI)‡§	3.86 (1.19 to 6.53)	3.16 (0.22 to 6.11)	2.61 (−0.97 to 6.19)
Past 7 days, knee pain during activity in the knee: (0-10):			
AIE: mean (SD)	5.4 (2.0)	5.2 (2.2)	5.1 (2.4)
AIE+B: mean (SD)	4.5 (2.1)	4.4 (2.1)	4.3 (2.4)
AIE *v* AIE+B: adjusted mean difference (95% CI)‡§	−0.97 (−1.30 to −0.63)	−0.80 (−1.15 to −0.44)	−0.72 (−1.15 to −0.29)
ICOAP: constant pain subscale (0-100):			
AIE: mean (SD)	30.2 (26.8)	31.4 (28.1)	28.6 (27.7)
AIE+B: mean (SD)	25.9 (24.1)	24.4 (24.0)	23.1 (24.0)
AIE *v* AIE+B: adjusted mean difference (95% CI)‡§	−3.82 (−7.53 to −0.10)	−6.53 (−10.48 to −2.57)	−4.83 (−9.18 to −0.48)
ICOAP: intermittent pain subscale (0-100):			
AIE: mean (SD)	41.8 (22.7)	41.4 (24.0)	39.7 (25.6)
AIE+B: mean (SD)	36.3 (21.9)	35.3 (21.6)	33.7 (22.0)
AIE *v* AIE+B: adjusted mean difference (95% CI)‡§	−5.41 (−8.92 to −1.91)	−5.89 (−9.49 to −2.29)	−5.44 (−9.49 to −1.40)
ICOAP: total pain scale (0-100):			
AIE: mean (SD)	36.5 (23.0)	36.9 (25.1)	34.7 (25.7)
AIE+B: mean (SD)	31.6 (21.6)	30.3 (21.3)	28.8 (21.7)
AIE *v* AIE+B: adjusted mean difference (95% CI)‡§	−4.68 (−7.96 to −1.40)	−6.21 (−9.69 to −2.74)	−5.25 (−9.21 to −1.30)
Knee buckling experienced:			
AIE: (No (%))	85 (44)	85 (46)	76 (42)
AIE+B: (No (%))	67 (33)	64 (35)	62 (34)
AIE *v* AIE+B: adjusted odds ratio (95% CI) ¶§	0.39 (0.19 to 0.77)	0.40 (0.20 to 0.82)	0.57 (0.28 to 1.17)
OMERACT-OARSI responder criteria met:			
AIE: (No (%))	62 (32)	65 (33)	69 (38)
AIE+B: (No (%))	86 (43)	93 (48)	93 (51)
AIE *v* AIE+B: adjusted odds ratio (95% CI) ¶§	3.44 (1.41 to 8.43)	4.28 (1.84 to 9.94)	3.57 (1.48 to 8.62)
IPAQ-E (MET min/week; 0-19278):			
AIE: mean (SD)	4528 (3627)	3891 (3044)	4263 (3511)
AIE+B: mean (SD)	4690 (3653)	4393 (3693)	4165 (3353)
AIE *v* AIE+B: adjusted mean difference (95% CI)‡§	601 (−6 to 1209)	760 (145 to 1376)	264 (−341 to 869)
Arthritis Self-Efficacy Scale (1-10):			
AIE: mean (SD)	5.7 (2.0)	5.8 (2.3)	5.9 (2.3)
AIE+B: mean (SD)	6.1 (2.1)	6.4 (2.1)	6.3 (2.3)
AIE *v* AIE+B: adjusted mean difference (95% CI)‡§	0.39 (0.06 to 0.73)	0.53 (0.16 to 0.90)	0.37 (−0.05 to 0.79)
KOOS-4 (0-100):			
AIE: mean (SD)	53.1 (14.7)	54.2 (16.5)	55.7 (17.4)
AIE+B: mean (SD)	56.8 (14.7)	58.0 (15.4)	59.1 (16.0)
AIE *v* AIE+B: adjusted mean difference (95% CI)‡§	3.80 (1.80 to 5.79)	4.17 (1.94 to 6.39)	3.29 (0.61 to 5.97)
WOMAC pain (0-20):			
AIE: mean (SD)	7.1 (3.8)	7.1 (4.1)	6.8 (4.2)
AIE+B: mean (SD)	6.2 (3.4)	6.0 (3.7)	5.8 (3.7)
AIE *v* AIE+B: adjusted mean difference (95% CI)‡§	−0.89 (−1.43 to −0.34)	−1.18 (−1.77 to −0.60)	−0.96 (−1.62 to −0.30)
WOMAC stiffness (0-8):			
AIE: mean (SD)	3.5 (1.6)	3.6 (1.6)	3.4 (1.7)
AIE+B: mean (SD)	3.4 (1.5)	3.2 (1.6)	3.0 (1.6)
AIE *v* AIE+B: adjusted mean difference (95% CI)‡§	−0.14 (−0.37 to 0.10)	−0.33 (−0.58 to −0.07)	−0.38 (−0.66 to −0.11)
WOMAC function (0-68):			
AIE: mean (SD)	23.9 (13.1)	23.7 (14.4)	22.4 (14.6)
AIE+B: mean (SD)	21.4 (13.0)	20.5 (13.2)	20.2 (13.6)
AIE *v* AIE+B: adjusted mean difference (95% CI)‡§	−2.80 (−4.55 to −1.06)	−3.56 (−5.45 to −1.68)	−2.44 (−4.68 to −0.21)

High score indicates more active for IPAQ-E, worse outcome for WOMAC, knee pain on activity, and ICOAP, and better outcome for KOOS and Arthritis Self-Efficacy Scale.

* Social participation (from Patient Reported Outcomes Measurement Information System, PROMIS) was an intended secondary outcome, but was erroneously not included in any follow-up questionnaires, and therefore cannot be reported as a secondary outcome.

† All model assumptions were largely satisfied in the data, despite the raw scores for some outcomes not following a normal distribution. Some evidence exists indicating a correlation between the model residuals and the random intercept, and hence models were fitted with an unstructured covariance matrix and robust standard errors. Model residuals were fitted separately for each follow-up time point, except for the mixed logistic regression models, where this specification was not an option.

‡ Fitted with linear mixed models.

§ Adjusted for PROP OA (Provision of Braces for Patients with Knee Osteoarthritis) trial clinic site, predominant compartmental distribution based on clinical and radiographic presentation, presence or absence of instability (buckling), age, sex, baseline anxiety, baseline depression, and baseline in the outcome of interest (except for the OMERACT-OARSI responder criteria because no baseline measure exists for this variable).

¶ Fitted with logistic mixed models.

AIE=advice, written information, and exercise instruction; AIE+B=advice, written information, and exercise instruction with knee bracing. CI=confidence interval; SD=standard deviation; IPAQ-E=International Physical Activity Questionnaire for the Elderly; ICOAP=Intermittent and Constant Osteoarthritis Pain; KOOS=Knee Osteoarthritis Outcomes Score; MET=metabolic equivalent of task; WOMAC=Western Ontario and McMaster Universities Arthritis Index; OMERACT-OARSI=Outcome Measures in Rheumatology-Osteoarthritis Research Society International.

AIE+B resulted in greater improvements than AIE for all KOOS-5 subscales, except for function in sport or recreation at three, six, and 12 months, other symptoms at six and 12 months, and knee related quality of life at 12 months. The greatest improvements were seen in the pain and activities of daily living subscales (adjusted mean difference on a 0-100 scale at six months for pain (6.13, 95% CI 3.36 to 8.91; effect size 0.39) and activities of daily living (5.24, 95% CI: 2.47 to 8.02; effect size 0.28). These effects were maintained at 12 months (pain 4.76, 95% CI 1.48 to 8.04, effect size 0.30; activities of daily living 3.60, 95% CI 0.30 to 6.89, effect size 0.19). Greater overall mean improvements in knee pain during weight bearing were also seen for AIE+B compared with AIE at three, six, and 12 months (adjusted mean difference on a 0-10 scale at 6 months: −0.80, 95% CI −1.15 to −0.44, effect size 0.34). The finding of greater benefit of AIE+B compared with AIE was consistent for most other secondary outcomes, except for physical activity at three and 12 months, Arthritis Self-Efficacy Scale at 12 months, and WOMAC stiffness at three months, where differences between groups did not reach statistical significance. Differences between groups were generally larger at three and six months than at 12 months (table 4 and supplementary appendix eTables 24-25). Although both interventions seemed to be acceptable, participants rated AIE+B more positively than AIE on self-reported items aligned with the theoretical framework of acceptability (eg, in the AIE group 158 (69%) participants liked or strongly liked the advice and treatment received from the physiotherapist compared with 185 (79%) participants in the AIE+B group; supplementary appendix eTable 26). 

We found no suspected unexpected serious adverse reactions to either intervention. Most adverse events were expected, and a similar overall number occurred in both arms (total number of self-reported adverse events at six months was 87 in the AIE+B group and 113 in the AIE group). Other than blisters, expected adverse events occurred across both arms, including skin irritation or redness, swelling, temporary increased soreness, and new or abnormal symptoms. The most common expected adverse event from AIE+B was skin irritation or redness, reported by up to 20% of participants, whereas blisters or broken skin were reported by up to 4% of participants (table 5 and table 6).

**Table 5:** Adverse events. Participants’ self-reports of adverse events in or around the knee for AIE group (advice, written information, and exercise instruction) and AIE+B group (advice, written information, and exercise instruction with knee bracing)

Adverse event in or around the knee	At 3 months*		At 6 months*		At 12 months*	
	AIE (n=196)	AIE+B (n=205)	AIE (n=185†)	AIE+B (n=185†)	AIE (n=185)	AIE+B (n=185)
Irritation or redness of skin	10 (5)	41 (20)	10 (5)	29 (16)	7 (4)	25 (14)
Blisters	0 (0)	5 (2)	0 (0)	2 (1)	0 (0)	3 (2)
Increased swelling	54 (28)	23 (11)	50 (27)	18 (10)	63 (34)	36 (19)
Temporary increased soreness	72 (37)	38 (19)	51 (28)	33 (18)	59 (32)	36 (19)
New or abnormal symptoms‡						
Joint clicking or crunching	2 (1)	0 (0)	0 (0)	0 (0)	0 (0)	1 (1)
Joint locking or giving way	2 (1)	0 (0)	0 (0)	0 (0)	2 (1)	1 (1)
Pain in other joints	1 (1)	5 (2)	1 (1)	0 (0)	1 (1)	1 (1)
Raised or irritated veins or arteries	0 (0)	1 (0)	0 (0)	3 (2)	0 (0)	1 (1)
Cruciate injury	0 (0)	0 (0)	0 (0)	0 (0)	0 (0)	1 (1)
Numbness or pins and needles	0 (0)	2 (1)	1 (1)	0 (0)	0 (0)	1 (1)
Stiffness	1 (1)	0 (0)	0 (0)	0 (0)	0 (0)	0 (0)
Injury	3 (2)	1 (0)	0 (0)	1 (1)	0 (0)	0 (0)
Fall	0 (0)	0 (0)	0 (0)	0 (0)	0 (0)	1 (1)
Shingles	0 (0)	0 (0)	0 (0)	1 (1)	0 (0)	0 (0)
New or abnormal symptoms indicated, but with no further detail for full symptom description	0 (0)	1 (0)	0 (0)	0 (0)	0 (0)	1 (1)

Values are number (%).

* Data collected from self-reported questionnaires using time frames of the past three months for the three and six month follow-up questionnaire, and the past six months” for the 12 month follow-up questionnaire.

† Denominator relates to full questionnaires returned because adverse event data were not collected on the minimum data collection form.

‡ Derived from coding of text data into categories.

**Table 6:** Adverse events. Physiotherapist reported* at two week follow-up for participants in AIE+B group (advice, written information, and exercise instruction with knee bracing)

Adverse event	AIE+B group (n=226)
Skin redness	42 (19)
Broken skin	10 (4)
Severe skin soreness	8 (4)
Marked increase in pain or swelling caused by knee brace	7 (3)
Blistering	4 (2)
Sensation changes in the leg	1 (0)
Other:†	
Bruising	7 (3)
Itching	1 (0)
Varicose veins	1 (0)
Pain in other joints	1 (0)

Values are number (%).

* Determined from the physiotherapist case report form based on the question: Did the participant report, or have you observed any of the following over the site of the knee brace?

† Derived from coding of text data into categories.

We saw a greater magnitude of benefit from AIE+B than from AIE on the primary outcome at six months in individuals who met our a priori definition of brace adherence at three and six months (complier average causal effect on KOOS-5 (0-100 scale): 5.21, 95% CI 1.48 to 8.94; supplementary appendix eTable 27). Although we found no significant results in the subgroup analyses, trends for larger treatment effects were seen in participants with osteoarthritis predominantly in the tibiofemoral compartment, more severe knee symptoms at baseline, lower levels of depression at baseline, self-reported adherence to treatment all of the time, and among men (supplementary appendix eTable 28).

## Discussion

### Principal findings

The findings of our study showed that adding compartment specific knee bracing with an adherence intervention to advice, written information, and exercise instruction in people with symptoms of knee osteoarthritis resulted in greater improvements in KOOS-5 at three and six months (primary outcome) compared with advice, written information, and exercise instruction only. These improvements, however, were no longer significant at 12 months. The magnitude of these benefits was small at three and six months, and very small at 12 months, as indicated by the effect sizes. Because the AIE+B group received more contact time and attention from the physiotherapist than the one session delivered in the AIE group, contextual factors might have contributed, at least in part, to the additional benefit observed. The treatment effect for the primary outcome did not reach the predefined minimum clinically important difference of eight points on KOOS-5, used to inform our sample size calculation. Although this finding raises questions about the clinical importance of adding knee bracing to AIE, growing awareness exists of the limitations of extrapolating criteria for clinically important changes in individuals to the evaluation of group differences.^29^

Several factors must be considered when interpreting the clinical significance of our results, including differences between the groups in primary and secondary outcomes, percentage of treatment responders, effect size relative to other available treatments, safety, acceptability, adherence, and cost.^29^ We showed that adherence to the use of a brace was an important determinant of outcome, and analyses of KOOS-5 subscales suggested that key drivers of the effect of AIE+B were likely to be because of improvements in pain and activities of daily living, which were maintained at 12 months. We found improvements for AIE+B compared with AIE for most other secondary outcomes at three and six months, and some at 12 months. We also saw a greater percentage of treatment responders in the AIE+B than in the AIE group at an odds ratio classified as clinically important by the National Institute for Health and Care Excellence.^8^ Minor expected adverse events were seen in both the AIE+B and AIE groups, and although both interventions seemed acceptable, participants rated AIE+B more positively on self-reported items aligned with the theoretical framework of acceptability.^19^ These factors, along with treatment effect sizes similar to those observed for core recommended treatment for osteoarthritis, such as exercise versus education or self-management only,^30^ suggests that, from a broader perspective, a small but important benefit exists in adding knee bracing to AIE. This interpretation is further supported by feedback from patient and public involvement, although the true extent of its clinical importance is uncertain.

The covid-19 pandemic represented a major intercurrent event during this trial. Social distancing, workplace closures, and three national stay-at-home lockdowns between March 2020 and March 2021 reduced levels of physical activity in the general population.^31^ This decreased activity, along with being unwell if infected, may have resulted in less opportunities and incentives for brace wearing, thus biasing estimates of trial effectiveness towards the null. Although the sensitivity analysis did not support this hypothesis, the true effects of the covid-19 pandemic on trial outcomes are difficult to determine.

### Strengths and limitations of this study

The key strengths of PROP OA are its large sample size and high follow-up rates. Informed by patient and public involvement and a clinical advisory group, our bracing intervention included readily available off-the-shelf braces delivered by physiotherapists. Physiotherapists represent a large NHS based professional group who routinely provide AIE to people with knee osteoarthritis and are therefore well placed to also deliver bracing. After standardised training (that could be delivered online, in-person, or in a mixed format), physiotherapists of varying levels of experience delivered AIE+B according to the protocol. Furthermore, despite lower physiotherapist confidence, the good level of agreement^32^ between their clinical judgment of the predominant compartmental distribution of knee osteoarthritis only and when combined with radiographic presentation, suggests that radiography may not always be necessary to determine the appropriate type of brace. These factors, together with the use of automated SMS text messaging to support brace adherence, would facilitate the implementation of AIE+B in routine clinical practice and integrating AIE+B into existing care pathways.

The PROP OA trial had some limitations. Although our analyses were undertaken masked to treatment allocation, in response to the covid-19 pandemic, outcome data were collected for some participants over the telephone by an unblinded trial manager. Masking participants or physiotherapists delivering trial interventions was not possible. Although the risk of confounding was minimised by training participating physiotherapists, the same physiotherapist treated patients in both arms and we found evidence of motivational interviewing being used in the control arm. This finding could have potentially enhanced care and outcomes beyond typical clinical practice, but change within the AIE group was no greater than in a similar control group from a previous randomised controlled trial in hip osteoarthritis.^33^ Thirty seven participants in the AIE group reported using some form of knee brace during the trial, which may have biased the findings towards the null. This behaviour might have been because of resentful demoralisation, but overall dropout rates were similar between the groups and the AIE intervention was broadly acceptable. 

Reflecting the pragmatic nature of PROP OA, although we used several recommended strategies for inclusive research,^34^ including a range of recruitment sites that covered urban deprived areas, community and social media advertising, offering some flexibility in the choice of clinic appointment times, and incentives, most trial participants were white, and only 56 participants (12%) were from the most deprived group of neighbourhoods in England. This inability to attract a more socioeconomically and ethnically diverse population is a limitation and questions the relative acceptability and effectiveness of braces in under-served communities. For example, differences in acceptability between communities may influence brace adherence and, in turn, treatment effectiveness. The under-representation of some communities in our trial is likely a result of multiple factors. These factors may include limited involvement of under-served groups in our patient and public involvement activities, inclusion criteria requiring access to a mobile phone for SMS text messages (not necessarily a smartphone), the ability to read and write English, and a lack of targeted outreach or culturally tailored engagement strategies to raise awareness among diverse communities. The acceptability of, and access to, bracing in under-served communities warrants further attention. Future research should explore effective approaches to improve diversity and inclusion in randomised controlled trials of non-pharmacological interventions for osteoarthritis.

Our trial did not include a placebo or non-compartment specific brace comparator, and the AIE+B intervention comprised multiple components. Because we evaluated the effectiveness of the intervention as a whole, we could not determine which specific components were responsible for the observed effects. Although we included components in AIE+B to target adherence, use of the brace decreased over time, and we could not evaluate the specific effectiveness of our adherence enhancing strategies in supporting the use of the brace. Given the positive association between brace adherence and clinical effectiveness, future research should further investigate how best to target the offer of a knee brace and to enhance adherence to brace use over time to improve the long term effectiveness of knee braces.

About 50% of people who were allocated to receive AIE+B were classed as a treatment responder, similar to previous trials of exercise for osteoarthritis.^9^ This finding highlights individual variability in response to knee bracing. Although we identified trends towards a differential response to bracing among some subgroups, these analyses were under-powered. Future research should combine individual participant data from randomised controlled trials to further assess potential moderators of the effect of bracing for knee osteoarthritis.

### Comparison with other studies

Before PROP OA, randomised controlled trials of knee bracing for knee osteoarthritis were hampered by targeting only one knee compartment for all participants, small sample sizes, risk of bias, lack of follow-up beyond three months, and heterogeneity. Also, the focus on brace adherence was limited, despite the recognised problem of low long term adherence to brace use, with little measurement and few interventions incorporated to optimise adherence.^5^ ^35^ PROP OA addressed these challenges by tailoring the type of brace to participant presentation and incorporating strategies to promote and measure adherence to the use of the brace. Compared with previous randomised controlled trials, PROP OA is the largest, included a broad suite of outcomes, and followed participants over the longer term with good follow-up rates. PROP OA added bracing to advice, written information, and exercise instruction that was delivered by physiotherapists in a format applicable for UK general practice, where knee osteoarthritis is predominantly managed. PROP OA provides more certainty that adding compartment specific bracing and an adherence intervention to AIE leads to, on average, small additional benefits on patient reported outcomes for people with knee osteoarthritis. The trial also allays previous concerns about the potential risk of bracing causing major adverse events, such as blistering and other pressure damage.^8^

### Policy implications

The evidence generated by the PROP OA trial can be used to update and reduce conflicting recommendations about knee bracing for knee osteoarthritis in international clinical guidelines and support treatment decision making for patients, healthcare providers, policy makers, and commissioners.

### Conclusion

Our trial showed that adding compartment specific knee bracing and an adherence intervention to advice, written information, and exercise instruction resulted in small improvements in patient reported outcomes in individuals with knee osteoarthritis. This safe and acceptable intervention offers a potential treatment option for this common condition.

**What is already known on this topic**

International guidelines offer conflicting recommendations on the use of knee bracing for knee osteoarthritis
Previous systematic reviews have been inconclusive because of limitations in existing trials, such as risk of bias, small sample sizes, short follow-up, and lack of targeting of adherence
The limited evidence base reflects challenges in evaluating and implementing knee bracing for knee osteoarthritis, including heterogeneity in patient presentation and variability in brace types, indications, mechanisms, and costs

**What this study adds**

PROP OA evaluated the effect of compartment specific knee bracing, supported by adherence strategies
Compared with advice, written information, and exercise instruction only, adding compartment specific knee bracing with an adherence intervention resulted in small improvements in patient reported outcomes
This safe and acceptable intervention offers potential treatment for people with knee osteoarthritis

## Data Availability

The code used to analyse the data in the paper can be found in the supplementary files. The data underlying the findings in this paper are openly and publicly available and can be found here: https://doi.org/10.21252/9tpn-8970. If you encounter problems accessing the data, please contact the corresponding author.

## Web extra

Extra material supplied by authors

Web appendix 1: Appendix

Web appendix 2: Statistical analysis plan

Web appendix 3: Statistical code used for analyses (Stata format)

Web appendix 4: Statistical code used for analyses (text format)

## Notes

Contributors: MAH was involved in conceptualisation, resources, formal analysis, funding acquisition, methodology, project administration, data curation, supervision, validation, visualisation, writing-original draft, and writing-review and editing. EN was involved in conceptualisation, resources, formal analysis, funding acquisition, methodology, data curation, software, validation, visualisation, writing-original draft, and writing-review and editing. ZA was involved in formal analysis, data curation, software, validation, visualisation, writing-original draft, and writing-review and editing. FB was involved in conceptualisation, funding acquisition, investigation, methodology, project administration, software, supervision, validation, and writing-review and editing. BB was involved in conceptualisation, funding acquisition, methodology, supervision, and writing-review and editing. MC was involved in conceptualisation, funding acquisition, investigation, methodology, project administration, software, supervision, validation, and writing-review and editing. KD was involved in conceptualisation, funding acquisition, methodology, supervision, and writing-review and editing. DF was involved in conceptualisation, funding acquisition, methodology, supervision, and writing-review and editing. NEF was involved in conceptualisation, funding acquisition, methodology, supervision, and writing-review and editing. NH was involved in investigation, resources, project administration, data curation, supervision, and writing-review and editing. CI (patient and public involvement representative) was involved in conceptualisation, resources, funding acquisition, methodology, and writing-review and editing. CJ was involved in conceptualisation, resources, formal analysis, funding acquisition, methodology, project administration, data curation, software, supervision, validation, and writing-review and editing. SJ was involved in conceptualisation, resources, formal analysis, funding acquisition, methodology, data curation, supervision, validation, visualisation, and writing-review and editing. GP was involved in conceptualisation, resources, formal analysis, funding acquisition, methodology, project administration, data curation, software, supervision, validation, visualisation, writing-original draft, and writing-review and editing. MAH, EN, and GP are the study guarantors. The corresponding author attests that all listed authors meet authorship criteria and that no others meeting the criteria have been omitted.

Transparency: The lead author (MAH, the manuscript’s guarantor), affirms that the manuscript is an honest, accurate, and transparent account of the study being reported; that no important aspects of the study have been omitted; and that any discrepancies from the study as planned (and, if relevant, registered) have been explained.

Dissemination to participants and related patient and public communities: Our dissemination strategy is designed to ensure our findings reach a wide range of relevant audiences, including healthcare professionals (particularly physiotherapists, other allied health professionals, and primary healthcare professionals), researchers, service managers, healthcare planners and commissioners, national and international policy makers, trial participants, and people living with knee osteoarthritis, including those currently accessing healthcare services. To engage these audiences effectively, we will produce a variety of outputs, including written plain English summaries, posters (scientific and lay), press releases, social media content (eg, posts on X and LinkedIn), original research articles, metadata, written reports and synopsis, slide presentations, and practical guidebooks or manuals. These outputs will be disseminated through multiple channels, including Keele University web pages, media, presentations at national and international scientific conferences, peer reviewed journals, local and national communities of practice, the ISRCTN trial registry, and open access repositories. To further support the accessibility of our findings beyond the scientific community, we will provide interpretive aids and alternative visual formats, and an extended plain language summary (available at: https://doi.org/10.21252/9tpn-8970).

Provenance and peer review: Not commissioned; externally peer reviewed.
