# Supplementary material for: Provision of knee bracing for knee osteoarthritis (PROP OA): multicentre, parallel group, superiority, statistician blinded, randomised controlled trial
Source: BMJ. 2026 Jan 26;392:e086005. doi: 10.1136/bmj-2025-086005 (PMC12829467; doi:10.1136/bmj-2025-086005)
Supplement: Supplementary file 1 — Web appendix 1: Appendix [file holm086005.ww1.pdf]

Supplementary appendix

Supplement to: Holden MA, Nicholls N, Abdali Z, et al. Provision of knee bracing for knee Osteoarthritis (PROP OA): a multicentre randomised controlled trial

Table of Contents

	Page
List of Supplementary Tables and Figures	2
PROP OA Investigators	4
Internal pilot: quantitative findings	6
Full inclusion and exclusion criteria	7
Criteria for determining predominant compartmental distribution of knee OA: instructions provided to physiotherapists	8
Training programme for trial physiotherapists	11
Trial intervention details (AIE and AIE+B)	13
Patient and Public Involvement (PPI)	21
Appendix reference list	60

List of Supplementary Tables and Figures

	Page
eTable 1: Internal pilot findings: consideration of quantitative findings in relation to the pre-specified progression criteria	6
eTable 2: Eligibility criteria	7
eTable 3: Clinical features to consider when determining predominant compartmental distribution of knee OA	8
eTable 4: Outline of the three day in person physiotherapist PROP OA trial training programme	12
eTable 5: Brace type suitable for predominant compartmental distribution of knee OA	15
eTable 6: Number randomised by recruitment method	24
eTable 7: Reasons for ineligibility	25
eTable 8: Additional baseline characteristics of knee problem by participant self-report	27
eTable 9: Key baseline characteristics by method of recruitment	28
eTable 10: Key baseline characteristics by loss to follow-up	31
eTable 11: Characteristics of participants at each stage of trial recruitment	34
eTable 12: Missing data rates for the primary outcome (KOOS-5) and associated subscales	35
eTable 13: Missing data patterns for the primary outcome (KOOS-5)	36

eTable 14: Protocol deviations that could potentially impact on the primary and secondary outcome measures	37
eTable 15: Clinical assessment and radiographic presentation of the predominant compartmental distribution of knee OA in the knee to be treated	38
eTable 16: Clinical judgement on predominant compartmental distribution of knee OA in the knee to be treated: comparing clinical judgement alone, with clinical judgement and radiographic presentation combined	39
eTable 17: Comparing brace allocation based on clinical judgement alone with clinical judgement combined with radiographic presentation	40
eTable 18: Physiotherapists' confidence in judging the predominant compartmental distribution of knee OA in the knee to be treated	41
eTable 19: Treatment delivery – AIE	42
eTable 20: Treatment delivery – AIE+B: initial treatment session	43
eTable 21: Treatment delivery – AIE+B: follow-up treatment session	45
eTable 22: Adherence to the trial treatment (AIE or AIE+B)	47
eTable 23: Brace use in the AIE+B arm only	48
eFigure 1: Graphs of the total time spent wearing the knee brace in the last 7 days for each occasion of SMS text data collection	50
eFigure 2: Graph of the proportion (and associated 95% confidence intervals) of those reporting they had worn the knee brace for the minimal time for each occasion of SMS text data collection	51
eTable 24: effect size calculations: sensitivity analyses using a range of standard deviation estimates	52
eTable 25: Median and interquartile range for outcome measures with a skewed distribution at follow-up	55
eTable 26: Intervention acceptability at 3-month follow-up	55
eTable 27: Complier Average Causal Effect (CACE) models for the primary outcome at 6-month follow-up (KOOS-5)	57
eTable 28: Exploratory subgroup analyses for the KOOS-5 primary outcome at 6-month follow-up	58

PROP OA Investigators

Names and affiliations of the wider members of the PROP OA trial team

**First and middle name(s) or initials**	**Surname(s)**	**Affiliation(s)**
Evans A	Asamane	School of Medicine, Primary Care Centre Versus Arthritis, Keele University, Keele, UK
Rachel	Browell	Northumbria Healthcare NHS Foundation Trust, North Shields, Tyne and Wear, UK
Sarah	Bathers	Keele Clinical Trials Unit, Keele University, Keele, UK
Katharine	Dobb	Research in OsteoArthritis Manchester (ROAM), Centre for Epidemiology Versus Arthritis, Centre for Musculoskeletal Research, Institute of Inflammation and Repair, The University of Manchester, Manchester, UK
Tina	Hadley-Barrows	Royal Wolverhampton NHS Trust, Therapy Services Department, New Cross Hospital, Wolverhampton, UK<br><br>Impact Accelerator Unit, Primary Care Centre Versus Arthritis, School of Medicine, Keele University, Keele, UK
Liz	Hartshorne	Nottingham Clinical Trials Unit, University of Nottingham, UK
Dan	Herron	Department of Psychology, School of Health, Education, Policing and Science, University of Staffordshire, UK
Lucy	Huckfield	School of Medicine, Primary Care Centre Versus Arthritis, Keele University, Keele, UK
Katrina	Humphreys	School of Medicine, Primary Care Centre Versus Arthritis, Keele University, Keele, UK
Jesse	Kigozi	Health Economics Unit, Department of Applied Health Sciences, University of Birmingham, Birmingham, UK
Sarah	Lawton	Keele Clinical Trials Unit, Keele University, Keele, UK
Christian	Mallen	School of Medicine, Primary Care Centre Versus Arthritis, Keele University, Keele, UK
Michelle	Marshall	School of Medicine, Primary Care Centre Versus Arthritis, Keele University, Keele, UK

John	McBeth	Primary Care Research Centre (Faculty of Medicine) Digital Health and Biomedical Engineering Group, University of Southampton, UK
Gail	Sowden	Mersey and West Lancashire Teaching Hospitals NHS Trust, Whiston Hospital, Warrington Road, Prescot, UK
Martin J	Thomas	School of Medicine, Primary Care Centre Versus Arthritis, Keele University, Keele, UK; Haywood Academic Rheumatology Centre, Midlands Partnership University NHS Foundation Trust, Haywood Hospital, Staffordshire, UK

Participating National Health Service (NHS) Trusts
Midlands Partnership University NHS Foundation Trust, Manchester University NHS Foundation Trust, the National Institute for Health and Care Research (NIHR) Manchester Biomedical Research Centre (BRC) (NIHR203308), Mid-Cheshire Hospitals NHS Foundation Trust, Northumbria Healthcare NHS Foundation Trust.

Participating physiotherapists
Blanca Alhambra Olalla, Thomas Arkinson, Michael Callaghan, Anna Cromie, Richard Dann, Lucy Huckfield, Katrina Humphreys, John Leah, Lumbani Munthali, Jane Platt, Johny Quicke, Shaun Roberts, Heidi Smith, Dan Storey, Martin J Thomas, Lucy Thompson, Daragh Walshe, Sabrina Welsh.

Clinical Advisory Group
Leela Biant (University of Manchester), Tina Hadley-Barrows (Royal Wolverhampton NHS Trust), Katrina Humphreys (Mid Cheshire Hospitals NHS Foundation Trust), Vicky Jeffrey (Cornwall Partnership NHS Foundation Trust), Treena Larkin ((Staffordshire & Stoke-on-Trent Partnership Trust), Claire Minshull (Shropshire Community Health NHS Trust), Tim Powell (Cornwall Partnership NHS Foundation Trust), Gail Sowden (Staffordshire & Stoke-on-Trent Partnership Trust), Samantha Watson-Smith (Heart of England NHS Foundation Trust).

Independent Trial Steering Committee
Professor David Beard, University of Oxford (chair), Professor Jeff Breckon, Teeside University, Mr Derek Cope, PPI member, Professor Michael Hurley, St George's, University of London and Kingston University London, Professor Rafael Pinedo-Villanueva, University of Oxford, Professor Lee Shepstone, University of East Anglia Mrs Christine Walker, PPI member.

Independent Data Monitoring Ethics Committee
Professor Catherine Hewitt, University of York (chair), Professor Philip Conaghan, University of Leeds, Professor Martijn Steultjens, Glasgow Caledonain University.

Internal pilot: quantitative findings

The PROP OA trial commenced recruitment on the 25th November 2019. The database for the internal pilot was frozen on the 1st July 2021, at which point 130 participants had been randomised and contributed data for the internal pilot. Pre-specified quantitative progression criteria (alongside qualitative findings, reported separately) were used to inform whether to proceed to the main trial, proceed with protocol amendments, or to stop the trial. As summarised in the eTable 1 below, quantitative data supported proceeding to the main trial.

eTable 1: Internal pilot findings: consideration of quantitative findings in relation to the pre-specified progression criteria

	**Proceed to main trial**	**Proceed to main trial with protocol amendments**	**Stop: Do not proceed to main trial**	**QUANTITATIVE FINDINGS**
**Recruitment** ***In months 7-9 of recruitment:***	Per site: >4 participants per month; Overall: 19+ participants per month	Per site: 3-4 participants per month; Overall: 12-18 pts per month	Per site: <3 participants per month; Overall: <12 participants per month	**GREEN/AMBER** Mean per site*: 3.67, 2.67, 8.67, 3.00 per month (range 2-6 per month); Mean overall: 18 per month (range 8-24 per month)†
**Intervention fidelity‡**	Interventions delivered per protocol for 75%+ of pts (per site; overall)	Interventions delivered per protocol for 45-74% of pts (per site; overall)	Interventions delivered per protocol for <45% of pts (per site; overall)	**GREEN** Per site: AIE - 100%, 78%, 100%, 100%; AIE+B - 79%, 83%, 89%, 87% Overall: AIE: 96%, AIE+B: 84% *(main reason due to being unable to attend 2 week follow-up appointment due to COVID-19)*
**Adherence to brace use¶ (AIE+B only)**	75%+ of pts reporting minimal level of brace adherence at 3 months* (per site; overall)	45-74% of pts reporting minimal level of brace adherence at 3 months* (per site; overall)	<45% of pts reporting minimal level of brace adherence at 3 months* (per site; overall)	**GREEN/AMBER** Per site: Not provided due to small numbers Overall: 92% (conservative estimate 58%§)
**Retention and follow-up at 3 months**	75%+ retention and follow-up at 3 months (per site; overall)	50-74% retention and follow-up at 3 months (per site; overall)	<50% retention and follow-up at 3 months (per site; overall)	**GREEN** Per site: 88%, 89%, 90%, 87% Overall: 88%

AIE+B: Advice, written information and exercise instruction plus knee bracing; AIE: Advice, written information and exercise instruction.
* Site estimates presented in the following order: Midlands Partnership Foundation Trust, Manchester University NHS Foundation Trust, Mid-Cheshire Hospitals NHS Foundation Trust, Northumbria Healthcare NHS Foundation Trust.
† Figures affected by staggered re-start in month 7 (Apr 2021).
‡ Defined as evidence from physiotherapist case report forms of having provided AIE if: participants were given verbal advice and education (about OA or about things to try at home to help with symptoms), were provided written information about OA (the OA guidebook) and prescribed a knee exercise programme.
AIE+B defined as being delivered per protocol if: participants received AIE as described above AND a knee brace, at least one Brief Motivational Interviewing technique, at least one Short Message Service (SMS) motivational prompt and a follow-up treatment session (either remotely or face to face).
AND a knee brace, use of ≥1 motivational interviewing technique, ≥1 SMS motivational prompt delivered, and attended follow-up treatment session (AIE+B).
¶ Defined as wearing the brace for 1 hour on two or more days per week estimated from responses to SMS.
§ Assumes all SMS non-respondents were non-adherent.

Full inclusion and exclusion criteria

eTable 2: Eligibility criteria

Inclusion criteria	Exclusion criteria
► Aged 45 years and over<br>► Residing in England and Wales<br>► Moderate to severe knee pain on weight bearing (NRS ≥4)<br>► With or without knee instability (buckling)<br>► Able to have knee X-ray<br>► Able to read and write English<br>► Access to a mobile phone that can receive SMS text messages<br>► Able to give full informed consent<br>► Willing to participate	► Red flags in the history or clinical examination that may indicate further investigation or referral for possible serious underlying pathology [1]<br>► Vulnerable individuals (eg, in palliative phase of care for cancer, unstable mental health disorders)<br>► Inflammatory/crystal arthritis (eg, rheumatoid arthritis, gout, psoriatic arthritis)<br>► Significant neurological disorder (eg, stroke, Parkinson's disease, multiple sclerosis, dementia)<br>► Fibromyalgia<br>► Symptoms not attributable to knee OA<br>► Previous major surgery in the knee to be treated (partial/ total knee replacement; high tibial osteotomy, not other previous arthroscopic surgery)<br>► Autologous cartilage implantation in last 12 months in the knee to be treated<br>► On the waiting list for total hip or knee replacement within the next 6 months<br>► Unwilling to wear a knee brace<br>► Brace size unavailable for leg circumference<br>► Knee brace contraindicated (superficial wounds where the knee brace would reside, psoriasis, eczema or poor circulation, arterial insufficiency, or severe varicosities that could result in skin at risk with regular brace wear, a history of thrombophlebitis in either leg)<br>► Significant fixed flexion deformity that prevents fitting of brace<br>► Injection in the knee to be treated within the last 3 months<br>► Recent/routine knee brace wear within the last 3 months<br>► Nursing home resident<br>► Unable to attend clinic<br>► Close family member already a trial participant<br>► Course of physiotherapy for the knee to be treated in the last 3 months

NRS = Numerical Rating Scale; OA = osteoarthritis.

Criteria for determining predominant compartmental distribution of knee OA: instructions provided to physiotherapists

Clinical assessment

No single piece of information from the clinical assessment is likely to allow you to confidently determine which compartment of the knee is most severely affected by OA. Instead, this is a judgement based on information on risk factors, pattern of symptoms, and findings on the physical examination. In the Clinical Eligibility Assessment this judgement should be made **without referring to patient X-rays**.

Some of the features from the clinical assessment that would lead you to suspect predominant medial tibiofemoral joint involvement, predominant lateral tibiofemoral joint involvement (which is relatively uncommon), or predominant patellofemoral joint involvement are shown below.

eTable 3: Clinical features to consider when determining predominant compartmental distribution of knee OA

	THINK **MEDIAL TIBIOFEMORAL JOINT** IF...	THINK **LATERAL TIBIOFEMORAL JOINT** IF...	THINK **PATELLOFEMORAL JOINT** IF...
Previous surgery/injury	Previous total or partial medial menisectomy [2,3], medial meniscal repair [4]	Previous total or partial lateral menisectomy, lateral meniscal repair	Previous patella subluxation and/or dislocation
Location of maximal pain/tenderness	Medial aspect of knee/medial joint line [5]	Lateral aspect of knee/lateral joint line [5]	Anterior aspect of knee/retropatellar [6-10]
Aggravating factors	Standing/walking [11]	Standing/walking [11]	Stair climbing, rising from sitting, kneeling, squatting [6-10]
Frontal malalignment	Varus [12]	Valgus	
Other tests	**Varus thrust during gait** [13,14]	Valgus thrust during gait (rare)	Positive Clarke's test [12]
	Effusion [8,12]		

Some additional points that you may find helpful:

- Lateral tibiofemoral joint OA is relatively uncommon. Medial tibiofemoral joint OA and patellofemoral joint OA are much more common.
- Varus malalignment and varus thrust during gait are very strong indicators of medial tibiofemoral joint OA. When present, these findings should be weighted heavily in your judgement.
- There is some evidence that tibiofemoral joint OA drives symptom and disease progression more than patellofemoral joint OA. This would suggest that when presented with a mixed picture of tibiofemoral and patellofemoral signs and symptoms, a bias towards tibiofemoral joint involvement may be justified.

Radiographic presentation

On X-rays, please determine radiographically, which is the most severely affected compartment. Is there:

- **No/ minimal** radiographic OA
- Predominant **patellofemoral** compartmental involvement
- Predominant **medial tibiofemoral** compartmental involvement
- Predominant **lateral tibiofemoral** compartmental involvement
- No predominant compartment

**TOP TIPS FOR JUDGING COMPARTMENTAL INVOLVEMENT ON X-RAYS**
● Focus on joint space narrowing to determine severity of compartmental involvement. There needs to be ⅓ of joint space loss to define definite change.
● Use other radiographic features (osteophytes, sclerosis, cysts) to support your decision making.
● If there is one-step difference between two compartments, then grade it as NO PREDOMINANT COMPARTMENT (e.g. mild + moderate; moderate + severe).
● If there is a two-step (or more) difference between two compartments, then grade as predominant in the most severe compartment (e.g. mild patellofemoral + severe medial tibiofemoral = predominant medial tibiofemoral on X-ray).

Combining clinical assessment and radiographic presentation to judge appropriate brace type

Thinking back to your clinical assessment, and adding in the findings from the x-rays, make a judgement about which brace you would provide to the participant, if they were randomised to AIE+B.

**TOP TIPS FOR COMBINING CLINICAL ASSESSMENT AND X-RAYS**
X-rays are used mainly to CONFIRM your clinical assessment
If in doubt, generally err towards your clinical assessment findings
In the unusual event of clinical assessment and X-rays being completely discordant, e.g. clinical assessment suggests predominantly medial tibiofemoral involvement but X-ray shows predominant lateral tibiofemoral involvement, then evidence of varus malalignment and varus thrust would be the main considerations and lead to you choosing to unload the medial tibiofemoral compartment. However, you would closely monitor the effect of the brace on any aggravation/new symptoms on the lateral tibiofemoral compartment.

Training programme for trial physiotherapists

Pre-COVID-19, the PROP OA physiotherapist training programme was delivered face-to-face, over 3 days. Following the outbreak of COVID-19, training covered the same material, was delivered over the same equivalent time-period, but was converted to a blended programme including pre-recorded lectures, online and live question and answer sessions, online live workshops, and face-to-face practice of knee brace fitting.

Day 1: Learning outcomes:

To ensure that participating physiotherapists:

- Have up to date knowledge about the aetiology of knee OA, its impact and prognosis.
- Have up to date knowledge about the recommended management of knee pain/OA, including the role of knee bracing for knee OA.
- Understand the design of the PROP OA trial.
- Can undertake relevant processes involved in recruitment of participants into the trial, including eligibility checking through clinical assessment and taking of informed consent.
- Can deliver AIE according to protocol.
- Are aware of the types of knee brace that are being utilised within the PROP OA trial and understand the indications for brace type allocation.
- Can determine knee brace allocation by clinical assessment.

Day 2: Learning outcomes:

To ensure that participating physiotherapists:

- Can begin to read knee x-rays to determine predominant compartmental involvement.
- Can determine knee brace allocation by clinical assessment combined with x-rays.
- Can fit knee braces being utilised in the trial, including provision of advice about brace dose and how to care for braces, and expected adverse reactions from knee braces.
- Are aware of the importance of adherence to brace use.
- Are aware of the knee brace adherence enhancing intervention being utilised within the PROP OA trial.

Day 3: Learning outcomes:

To ensure that participating physiotherapists:

- Can deliver the brace adherence enhancing intervention.
- Can fit knee braces being utilised in the trial.
- Can deliver PROP OA trial interventions to adults with knee OA according to protocol.
- Understand the reporting requirements and communication processes for the trial, including case report forms and adverse event reporting.
- Are aware of the next steps in terms of the PROP OA trial commencing, and the continued mentoring and support that will be provided within the trial.

Teaching methods: Lectures, interactive sessions, case studies, practical sessions, role play.

Pre-training reading: NICE OA guidelines**,** PROP OA protocol**,** PROP OA intervention details and written participant information (OA guidebook; exercise programme).

Pre-training tasks: Undertake relevant GCP training (online module or face to face; certificated); BMJ online learning module on Motivational Interviewing

eTable 4: Outline of the three day in person physiotherapist PROP OA trial training programme

Time	Day 1	Day 2	Day 3
AM	Knee OA and its management (including the role of knee braces)<br><br>The PROP OA trial<br>• Trial recruitment<br>• Eligibility checking<br>• Informed consent taking<br>• Randomisation procedures<br>*Practical with role play*<br><br>Advice, written information, exercise instruction (AIE)<br><br>Introduction to knee braces<br>• Types of knee brace being used in the trial<br>• Indications for each knee brace type	Reading of x-rays to inform brace allocation<br>*Practical*<br><br>Combining clinical and x-rays to determine brace allocation<br>*Practice with case examples*<br><br>Fitting of knee braces<br>• Dose of knee braces<br>• Care for knee braces<br>• Expected adverse reactions from knee braces<br>*Practical with role play*	The brace adherence enhancing intervention<br>*Practical with role play*<br><br>Fitting of knee braces<br>*Practical with role play*
PM	Clinical decision making to determine brace allocation<br>*Practice with case examples*<br><br>Reading of x-rays to inform brace allocation<br>*Practical with role play*	Fitting of knee braces (continued)<br><br>Adherence to brace use<br>• The importance of brace adherence<br>• The brace adherence enhancing intervention<br>*Practical with role play*	Trial reporting requirements<br>• Case Report Forms<br>• Adverse event reporting<br>• Monitoring and audit<br>• The interview study<br><br>Putting it all together<br>*Practical*<br><br>Next steps<br>• mock clinic in situ<br>• *Objective measurement of brace adherence*<br>• post-training tasks

Post-training tasks: Remote x-ray scoring (time = 2-4 hours); practice delivering relevant components of AIE to patients with knee OA within routine clinical practice; practice fitting of knee braces on a "buddy"

Trial intervention details

Protocol for Advice, written Information and Exercise instruction (AIE) (20 minutes)

In line with NICE OA core treatment guidelines [1] and based on findings from the clinical examination, physiotherapists were instructed to include the following:

<u>Advice and education</u>

- Provide the Osteoarthritis Guidebook (with minor trial adaptions) (available at: https://www.keele.health/osteoarthritis-resources/)
- Discuss the following broad areas (examples points for discussion also provided):

Pathogenesis and prognosis of knee OA

- Knee OA in people aged over 45 years old is common.
- The condition and its symptoms vary from person to another, but typically involves pain, stiffness and reduced function.
- OA affects the whole joint not just the cartilage. It is probably a result of the joint trying to repair itself ('wear and repair' not 'wear and tear'.)
- Knee OA can be caused by genetics; medical conditions (eg childhood joint problems); injury; job or sports; being overweight.
- In most people, OA does not become severe, so most people do not need a knee replacement.
- People can go through phases where joints are painful and then phases when the pain eases off.
- There is a lot that people can do to manage their knee pain, continue to be active, and do the things they want to do.

Exercise and increasing physical activity

- Lack of physical activity is bad for joints. It can cause wasting of muscles and weakening of other tissues that can increase pain and stiffness. Keeping the joint moving is important.
- Although knee OA can be very painful and activities may need to be reduced for a time, rest for more than one day or two usually does more harm than good.
- Research has shown that muscle strengthening and physical activity that makes the heartbeat faster (eg walking) can help relieve pain, stiffness, increase mood and keep people independent. It can also help improve general health.
- Any form of physical activity that makes your heartbeat faster is good (eg walking, cycling, and swimming), but its needs to be ongoing. Choose to do something enjoyable.

Losing weight (if applicable)

- Research has shown that being overweight can make knee OA worse.
- Losing weight can help to reduce knee pain and can reduce the need to take pain killers.
- Losing weight can be difficult. People can try to lose weight by: reducing portion sizes; eating more healthy foods; going to slimming clubs like weight watchers or slimming world.
- Trying to lose weight just by changing diet does not work as well as combining diet with increasing physical activity.

Things to try at home to help manage pain*

- **Footwear.** The best kind of shoes have: thick shock-absorbing soles; very low heels; wide fronts (so toes can splay out when walking); deep soft uppers; and can fasten.
- **Warmth to relieve pain, cold to relieve swelling.** Lots of things can be used. Use trial and error to see what helps, for example: warm bath/ shower; heat packs; hot water bottle, ice pack (e.g. bag of frozen vegetables).

- **TENS machine.** A machine that sends electrical pulses through the skin, which some people find helps reduce pain. There are different makes and models that are different prices. They can be bought online (e.g. Amazon) and from different shops (e.g. Boots).
- **Walking aids.** For example, walking stick, walking pole.
- **Pain killers.** There are different types of medicine that can help knee pain. Your doctor can advise on the most appropriate medications. Some can be bought in supermarkets or pharmacies, for example: NSAIDS in cream form; paracetamol (Take according to instructions on the packet).

Provision of lower limb exercise programme

- Select the appropriate exercises and appropriate starting dosage as per instructions on exercise sheet (available at: https://doi.org/10.21252/9tpn-8970.).
- Conduct each selected exercise with the participant.
- Issue the written exercise sheet.
- Advise that:
  - Exercise dosage can be progressed as exercises become easier.
  - It is normal to feel a slight increase in pain when commencing a new exercise programme.
  - The exercise programme should be continued and incorporated into the participants lifestyle.

Co-interventions

- Participants can continue to access usual health care, including medications and consultations with other health professionals.
- **For participants allocated to AIE, please advise them NOT to wear a knee brace for the next 6 months**.

*Reflecting current NICE guidance at the time of the training

Protocol for Advice, written Information and Exercise instruction plus bracing (AIE+B)

Physiotherapists were instructed to provide the following:

INITIAL TREATMENT SESSION (60 MINUTES)

AIE

Provide AIE as described above.

Provision of a knee brace

Provide participants with either a patellofemoral, tibiofemoral unloading, or neutral stabilising knee brace according to their pattern of knee OA, ***but also taking into account current and desired level of physical activity, ability to don/doff brace, willingness to wear the brace type, and immediate symptom response when the brace is tried on and tested in clinic.*** Brace types suitable for predominant compartmental involvement are shown in the table below.

eTable 5: Brace type suitable for predominant compartmental distribution of knee OA

Predominant compartmental involvement	1st Choice brace type	2nd choice brace type
Patellofemoral joint	Bioskin Q (sleeve)	Bioskin Q (front close) - if participant unable to apply sleeve brace
Medial tibiofermoral joint*	Össur Unloader One (medial)	Donjoy OA Nano (medial) - if unable to get a good fit with the Unloader One
Lateral tibiofemoral joint*	Össur Unloader One (lateral)	Donjoy OA Nano (lateral) - if unable to get a good fit with the Unloader One
No predominant compartment	Össur Formfit Knee Hinged	NA
* If the participant is below 5ft 3in, consider ordering the short version of the Ossur Unloader One		

Points to remember:

- The Bioskin sleeve brace is the first-choice patellofemoral brace. Only if the participant is unable to apply this, should the Bioskin front close brace be offered.
- The Ossur Unloader One is the first choice tibiofemoral unloader brace. The Donjoy is to be offered as a tibiofemoral unloader brace only if the Ossur Unloader fits poorly, as identified at the Treatment visit.
- If a participant has predominant tibiofemoral involvement but has a low level of desired physical activity for example, is unable to fit an unloader brace (e.g. due to co-morbidity like hand OA), or has a marked increase in pain on trying the brace on in clinic, provide a neutral stabilising brace (Össur Formfit Knee Hinged).
- At the Follow-Up appointment (described below), if the participant reports experiencing a marked increase in symptoms a neutral stabiliser brace can be provided as a replacement brace. This is most likely to occur if a tibiofemoral unloader brace was originally provided. It is expected that change of brace type will occur rarely.

How to fit the brace: Pay close attention to how the brace is fitted to ensure maximum comfort, e.g. contour hinges on the Össur Formfit Knee Hinged brace, adjust and cut straps to match participants' body shape and size).

Whilst fitting the brace:

- Practice walking with the brace on, on the flat and up and down stairs if relevant
- Get the participant to practice taking the brace on and off
- Provide appropriate advice (see below)

Advice related to brace use

Provide each participant with advice about the brace, including on:

- **How the brace works**

Advise participants that knee braces are designed to provide knee support and stability. Advise that some also apply a gentle force, designed to restore natural alignment and reduce the pressure going through the knee joint.

- **Brace fit**

Advise participants to wear their brace directly over their skin, not over clothes. Signpost them to the videos, manufacturer instructions included with the brace, and written brace guides for further information about how to take their brace on and off. If they feel like their brace is slipping, advise them to re-check these instructions to make sure they are putting it on correctly.

- **Dose**

The dose of brace use needs to be individually tailored. Advise participants to wear the brace on painful weight-bearing activity, with a starting minimum usage of 1 hour on two or more days per week, gradually increased based on tolerance to wearing the brace on all painful weight-bearing activity up to a maximum of 8-12 hours per day (as they would like wearing in a new pair of shoes).

Advise individuals to wear the brace for 6 months and continue to wear it beyond this time if they find it beneficial.

- **Brace care**

Provide advice about how to care for the brace. Recommend that participants should wipe their knee brace clean with an anti-bacterial cleaning wipe or warm soapy water, then let it dry naturally.

- **Monitoring skin**

Advise participants to monitor their skin regularly. Recommend that every day they inspect all areas of skin that come into contact with the knee brace. Advise to check for red marks, abrasions, grazing, bruising, blisters, or wounds. Advise that if redness appears, they should decrease their knee brace usage, then increase usage slowly again as the skin tolerates the brace.

In addition to providing verbal advice, provide participants with the appropriate written brace information leaflet (available at: https://doi.org/10.21252/9tpn-8970).

Provide the participant with a knee brace diary and encourage them to complete it at home (available at: https://doi.org/10.21252/9tpn-8970).

<u>Encouraging adherence to brace use</u>

Use principles of Motivational Interviewing to encourage adherence: affirmations, reflective listening, open ended questions, summarising, eliciting change talk.

Examples include:

“What do you currently know about wearing a brace and osteoarthritis, and activity? Would you like to know what I know?”

“Wearing braces has several benefits, including x,y,z. However, it is your decision about whether to wear it (emphasize personal control).”

Set a SMART goal - “Would you like to set a goal to wear your brace this week? How many days would you like to wear it? What time of day do you see yourself putting it on/taking it off? What might get in the way of achieving that goal? What sorts of things would help you achieve that goal? Who, in your life, can help support you in this?”

“How motivated are you to wear the brace every day, as prescribed, on a 1-10 scale, where 1 is not at all motivated and 10 is extremely motivated. If they choose a 1 or 2, “what is keeping you at the lowest numbers?” “What would it take to get up to a higher number, such as 4 or 5?” if they choose a 3 or higher ‘what is getting you up to a ‘x’, why not a 10?” “What will it take to get you to a higher number?” (remember to ask one question at a time).”

Summarise discussion.

Please note: The different components of the intervention are not intended to be delivered sequentially, rather incorporated together within one treatment session. For example, advice about the brace and Motivational Interviewing techniques may be provided whilst the participant is walking with the brace on. You may also prefer to provide the exercise programme when the brace is in situ. There is no right or wrong order to deliver these different components, rather, with practice, you will find out what sequence works best for you.

FOLLOW UP TREATMENT VISIT (30 mins)

<u>General considerations</u>

- **Ask the participant about their brace use.** Find out about:

- Response to brace

- Brace fit and ability to apply the brace

- Any adverse events

- Brace adherence (check their brace diary in addition to asking questions)

- **Check:**

- Fit of the brace

- Whether the participant takes the brace on/ off correctly

For participants tolerating the brace well and finding it helpful, progress brace use. Advise participants to wear the brace for longer durations of painful weight bearing activity.

If participants are experiencing discomfort or not finding it helpful, adapt braces and provide appropriate instruction. For example, adjust the brace fit, re-educate about how to apply the brace, or provide further advice on appropriate brace dose.

In extreme cases, if the brace is not tolerated it may be changed (most likely from a tibiofemoral unloading to neutral knee stabilising brace).

Advise participants that if they continue to not tolerate the knee brace or experience ongoing discomfort or skin irritation, contact you. You can then provide appropriate advice over the telephone, which may include discontinuing with any brace use.

During the appointment address adherence to brace using Motivational Interviewing techniques (affirmations, reflective listening, open ended questions, summarising, eliciting change talk).

SMS BRACE ADHERENCE CAPTURE AND MOTIVATIONAL PROMPTS (SCHEDULED ON WEEKS 1, 2, 3, 4, 6, 8, 10, 12, 16, 20, 24, 52)

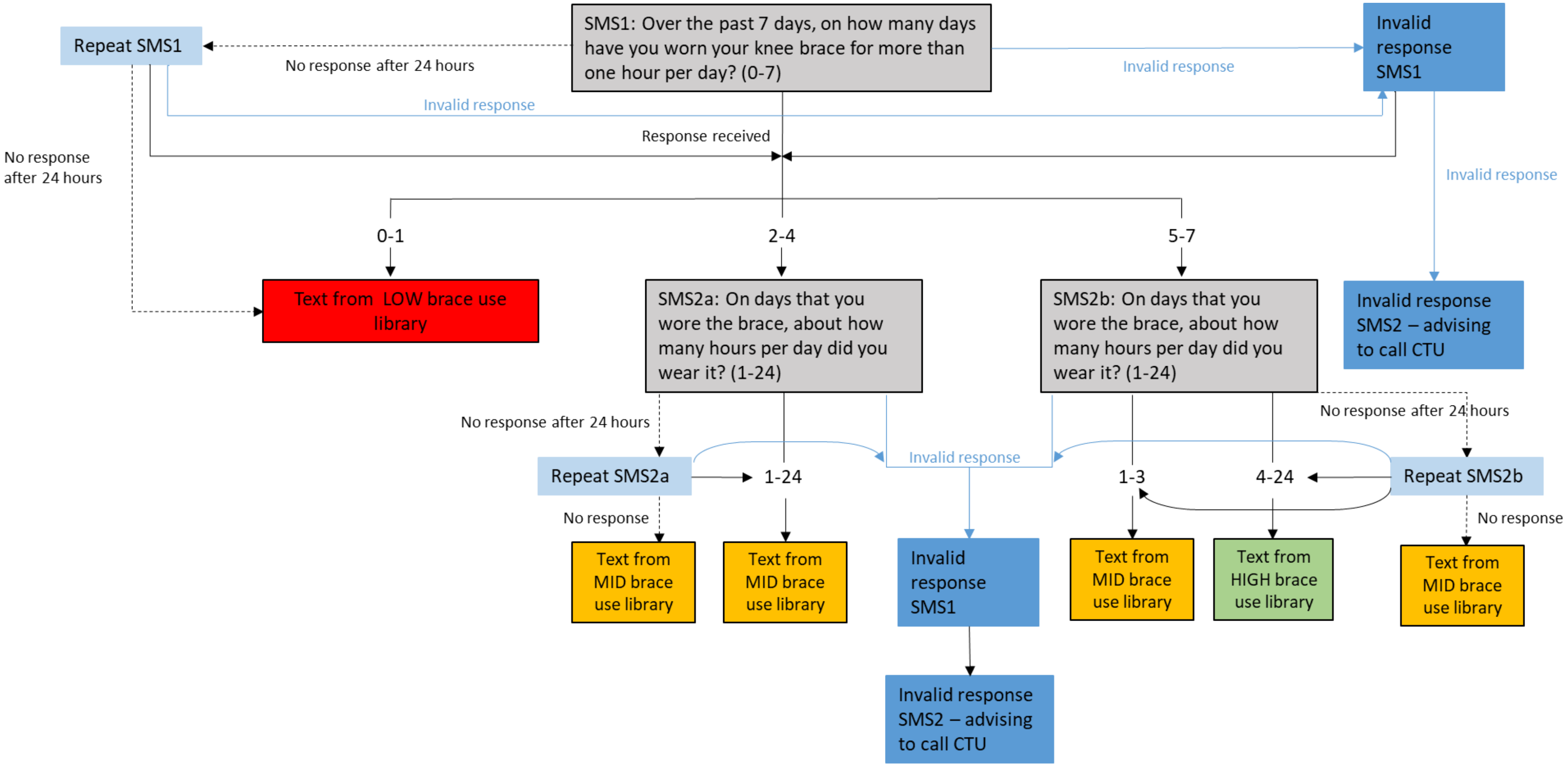

Low Adherence Text Library (sent if participants report wearing their knee brace on less than 2 days per week)

- From what you've told us, you are not wearing your knee brace much. Many people have trouble initially, but then they start wearing it and recognise the benefits.
- Thought for the day: what is the best result you can imagine if you wear your knee brace? What is the best result you can imagine if you don't wear your knee brace?
- More hours a day helps you on your way! Wearing your knee brace may help you go faster and further, with less pain.
- Your knee brace might help your pain, improve your mobility and make you feel better in yourself – give it a go!
- Why not try and wear your knee brace today? Try it for a few hours, each day.
- Your knee brace might help you to do more of the activities you enjoy. Think about what you would do if you could be more mobile.
- Forgotten how to put your knee brace on? Read the information sheet, watch the video. Wearing your brace might help!
- Worried that others may judge you for wearing a knee brace? You can wear your knee brace under clothes. Give it a go, it's worth it!
- Benefits of wearing a knee brace might build up over time. Try it for a month—you might see some progress!
- Set a goal: This week, try to wear your knee brace on two days, for at least 1 hour. Then increase it by one day and one hour each week! Reward yourself for progress.
- Try and build up how much you wear your knee brace over time - set a goal each week, such as wear it every day for one hour.
- Using your knee brace is important. Every hour may help to reduce the load going through your knee joint. This might help you to become more mobile and feel better.
- It's easy to say no but get up and go! Try your knee brace!
- Wearing your knee brace for at least 1 hour on 2 or more days a week may reduce your symptoms and help you get more active. Think about how that would feel!
- We notice you are not using your brace much. Try it, wearing your brace might help!
- Brace tip: if your brace is uncomfortable or slipping, read the information sheet and watch the video to make sure you have put it on correctly.
- .
- Thought for the day: How would your life get easier if you wore your knee brace more?
- Did you know that wearing your knee brace might help you to get more active? That not only helps your knee but has lots of other health benefits too!

Medium Adherence Text Library

- Thought for the day: What are some challenges that you have overcome in the past? What were your strengths that got you through? Harness those strengths to help you wear your knee brace more often.
- Deciding to wear your knee brace is a difficult decision, and with every difficult decision there are pros and cons. What's stopping you from wearing your knee brace more? What are the benefits of wearing your brace? Have a think about this.
- Thought for the day: How would your life get easier if you wore your knee brace more?
- Well done for wearing your knee brace, keep going! To get maximum benefit, next week increase the number of hours or days you wear it.
- Well done for wearing your knee brace! Wearing your knee brace more often may reduce pain, improve mobility and mood – give it a go!
- Knee braces are designed to support your knee so you can have less pain and be more active. What are you able to do with your knee brace that you couldn't do before? Have a think about it.
- Wearing a knee brace can be stressful, but many people say that NOT wearing the knee brace is even more stressful. Reward yourself for good efforts.
- Think about what you want to do in the next five years. How can wearing your knee brace more help you to achieve your goals?
- To help you decide whether or not to wear your knee brace more, write down the pros and cons of wearing it.

- Set a goal: This week, try to wear your knee brace for 1 hour more each day! Reward yourself for progress.
- It’s easy to say no, but get up and go! Keep trying to wear your knee brace for longer, every day.
- Brace tip: if your brace is uncomfortable or slipping, read the information sheet and watch the video to make sure you have put it on correctly.
- Benefits of wearing a knee brace might build up over time. Try it for a month—you might see some progress!
- Using your knee brace is important. Every hour may help to reduce the load going through your knee joint. This might help you to become more mobile and feel better.
- Did you know that wearing your knee brace might help you to get more active? That not only helps your knee but has lots of other health benefits too!

High Adherence Text Library (sent if participants report wearing their knee brace on 5-7 days per week, for between 4 and 24 hours each day)

- Well done for wearing your knee brace! Think of three benefits you are getting from wearing your knee brace to keep your motivation high!
- You’re doing a great job wearing your knee brace! Give yourself a little reward each day.
- You’ve been wearing your brace most days this week! You’ve overcome the challenges and hopefully you are noticing the benefits.
- You’re wearing your knee brace a lot – good for you! Think of three positive things that have come from wearing your knee brace.
- Great to see you are wearing your brace. Did you know that wearing your knee brace might help you to get more active? That not only helps your knee but has lots of other health benefits too!
- You’ve been doing a great job wearing your knee brace. However, some days you may feel like not wearing it. Write down 3 good things about wearing it and review this list during tough times.
- You’re doing a great job wearing your knee brace! Keep it up— make a list of activities you can now do if you wear your knee brace.
- Great job wearing your knee brace! Did you know that consistent brace use may reduce the need for medication?
- Well done for wearing your knee brace! There might still be days that you don’t feel like wearing your knee brace. This is natural. Try to overcome these feelings by thinking about the benefits that you might get from your knee brace.
- Great to see you are wearing your brace! Using your knee brace is important, so keep going.
- Great to see you are wearing your brace! It’s easy to say no but get up and go! Keep wearing your knee brace!

Patient and Public Involvement (PPI)

The overall aim of PPI for the PROP OA trial was to ensure that the voices and perspectives of people living with knee OA and long-term musculoskeletal pain conditions were heard and meaningfully influenced and improved the trial design, execution, monitoring, interpretation and dissemination.

All PPI activity followed NIHR guidelines [15], consistent with UK Standards for Public Involvement [16], and was supported by a User Support Worker and the trial Associate Investigator. We identified PPI representatives with lived experience of knee OA, some of whom also had experience of using a knee brace, from the Keele University Research User Group, which has over 180 members.

PPI inputted in all stages of the trial.

1. **Pre-funding**

- We held a meeting with 5 PPI representatives with lived experience of knee OA (some of whom also had experiences of knee braces for knee OA). At the meeting we discussed the proposed interventions and trial design. Discussions impacted on our:

1. Recruitment strategy. PPI representatives highlighted the importance of including a self-referral option, because *"people may put knee pain down to old age",* so may not be seeking health care.
2. Intervention content. PPI representatives felt that the braces should be adjustable, and there should be an element of choice and a holistic approach to brace allocation (for example also considering factors such as a participant's ability to apply the brace and their functional goals). They felt that a review at 2 weeks was important in facilitating adherence to brace use, and using text message prompts to support adherence was *"a very good idea."*
3. Choice of primary outcome: Whilst the funder had requested function as the primary outcome measure in their commissioned call for the RCT, PPI representatives told us that pain was equally as important as function: "*pain is what you start with so if pain improves then function improves;" "if my function is improved but my pain is still there I would think what has gone on?"* Our primary outcome measure was therefore a composite measure including both pain and function (KOOS-5).
4. Study team. We identified two members of the group who agreed to become co-applicants and members of the study team.

- To help inform the development of the PROP OA trial protocol we also formed, and convened two workshops with a Clinical Advisory Group consisting of multi-disciplinary clinicians involved in the provision of braces for patients with knee OA. Our PPI co-applicants actively participated in these workshops.

- Our two PPI co-applicants co-wrote the Plain Language Summary of the trial methods. Their input simplified and improved its readability, improving its Flesh Index (ease of reading score 0-100, higher = easier to read) by 8 points (available at: https://doi.org/10.21252/9tpn-8970).

- Comments about the overall trial design were obtained from the general public via a blogpost on the interactive VoiceNorth public discussion forum. Specific feedback on the summary trial plan was provided by nine VoiceNorth members.

At this stage, one of our PPI co-applicants dropped out of the trial due to other commitments. Our remaining PPI co-applicant continues to be an active and important member of the trial team.

2. **Study set up**

Our remaining PPI co-applicant (Carol Ingram) contributed to study set up in terms of:

- Helping to develop patient facing materials (e.g. participant information leaflet, wording on study website)
- Contributed to intervention development. Carol helped us to develop the algorithm for, and content of, motivational prompts that were sent to participants randomised to AIE+B, to encourage adherence to brace use. Examples of prompts that Carol developed include *"It's easy to say no but get up and go! Try your knee brace!", "More hours a day helps you on your way!"*
- Helped to train participating physiotherapists. Carol met the physiotherapists when we held our initial face-to-face training programme and told them about her experiences of wearing a knee brace. She also participated in training videos that were developed and provided to participating physiotherapists. One video was about taking informed consent, the other about knee joint examination.

3. Active study phase

Carol attended monthly trial management meetings for ongoing oversight. She contributed to ongoing trial oversight, interpretation of the internal pilot findings, and with problem solving and decision making. For example, when we had to adapt the trial in response to the COVID-19 pandemic, Carol co-wrote additional wording for our participant facing materials, played a key role in our decision to continue to offer face-to-face treatment (as able), and helped us to decide to transition from paper-based to electronic data collection.

4. Interpretation of results

We presented our trial findings to 7 PPI representatives with lived experience of knee OA (including Carol). Public contributors felt strongly that benefits of AIE+B compared to AIE that were categorised and labelled as 'small', based on accepted conventions for interpreting effect sizes, risked dismissing benefits that could be meaningful for the very large number of people with knee OA faced with relatively few safe, effective alternative treatments. There was concern that policymakers would fail to look beyond the word 'small' in their focus to reduce NHS costs. They felt, for example, that the difference between a 40% and a 50% chance of clinically important improvement, would be valued by many patients. They pointed out the importance of the larger effects on pain reduction – which public contributor had argued prior to the grant submission should be the primary outcome. Reducing knee buckling and the potentially serious consequences of this for falls and related hospital admissions was also felt to be important to them. Finally, they argued that intermittent use of braces – for example during an acute flare – would be legitimate and valuable from a patient perspective, but would fall below the definition of 'minimum brace use' used to describe adherence in the trial.

PPI representative interpretation of the results has informed key discussion points in the manuscript. Based on their feedback, we produced an extended Plain Language Summary to provide a fuller account of the project findings (available at https://doi.org/10.21252/9tpn-8970).

3. Dissemination and implementation

Carol, our PPI co-applicant, has already been involved in dissemination of our study findings. This includes:

- Contributing to an invited oral presentation that focused on the role of PPI in the PROP OA trial that we gave at the Patellofemoral Research Network (iPFRN) Research Retreat, Bologne, Italy in 2023.
- Contributing to writing a case study for Versus Arthritis, describing PPI in the PROP OA trial.
- Co-writing the Plain Language Summary of findings.
- Contributing to all trial publications and being recognised for her contribution by being a named co-author.

In 2023, Carol helped to present the trial at a Keele University Impact Accelerator Think Tank, where steps to support early implementation of the study findings were discussed.

Critical reflections

PPI representatives with lived experience of knee OA were involved at all stages of the trial, making important contributions to its design, execution, monitoring, interpretation and dissemination. The convening of a multidisciplinary Clinical Advisory Group with PPI contributors was particularly valuable as we moved from a detailed project description approved by the funder to a fully specified protocol with manualised procedures and working instructions. Continuity of involvement was achieved with a PPI co-applicant (Carol) who was involved in regular project management group meetings across the entire duration of the trial. Whilst Carol was supported by a User Support Worker, replacing the second PPI co-applicant could have reduced the burden on Carol, given the trial duration and intensity.

eTable 6: Number randomised by recruitment method

	Randomised N = 466
GP letter following retrospective electronic medical record review	229 (50%)
Advertisement	231 (50%)
Social media	58 (25%)
Advertisement on a website	19 (8%)
Radio	0 (0%)
Local poster/flyer	34 (15%)
Newspaper/magazine	59 (26%)
Word of mouth	67 (29%)

Figures are numbers (percentages). Participants could report multiple advertisement methods of recruitment.

eTable 7: Reasons for ineligibility

**Ineligibility reasons on the telephone screen*†**	N (%)
Aged < 45 years	1 (0%)
Not living in England or Wales	1 (0%)
Unable to read/write English	0 (0%)
No access to a mobile phone that can receive text messages	11 (4%)
No knee pain	6 (2%)
Pain severity in the last 7 days <4 (knee to be treated)	65 (26%)
Previous knee replacement	18 (7%)
Had cartilage implants in the last 12 months (knee to be treated)	1 (0%)
Major injury or trauma to the knee to be treated in the last 3 months	1 (0%)
Physiotherapy in the last 3 months (knee to be treated)	35 (14%)
Injection in the last 3 months (knee to be treated)	22 (9%)
Worn a knee brace in the last 3 months (knee to be treated)	17 (7%)
On the waiting list for a hip or knee replacement in next 6-months	5 (2%)
Under regular follow-up with a rheumatologist	17 (7%)
Has gout‡	10 (4%)
Taking relevant medication for inflammatory arthritis	5 (2%)
Has fibromyalgia	14 (6%)
Has Parkinson's disease	2 (1%)
Pregnant or breast feeding	0 (0%)
Family member already in the study	2 (1%)
Unwilling to wear a knee brace	4 (2%)
Unwilling to attend study appointments	3 (1%)
Didn’t give consent to take part	7 (3%)
Unable to take part as has dementia	1 (0%)
**Total**	**248**
**Ineligibility reasons at the clinical assessment**	N (%)
Has a red flag	3 (2%)
Has inflammatory/crystal arthritis	12 (9%)
Has a significant neurological disorder	2 (2%)
Vulnerable individual	3 (2%)
Fibromyalgia	0 (0%)
Previous major surgery to the knee to be treated	3 (2%)
Autologous cartilage implantation in last 12-months (knee to be treated)	0 (0%)
On waiting list for knee or hip replacement in the next 6-months	0 (0%)
Had physiotherapy in the last 3 months (knee to be treated)	5 (4%)
Had injection in the last 3 months (knee to be treated)	1 (1%)

Worn a brace in the last 3 months (knee to be treated)	10 (8%)
Has a contra-indication to having new knee x-rays	0 (0%)
Knee brace contraindicated (superficial wounds, psoriasis, eczema, poor circulation, arterial insufficiency, severe varicosities, history of thrombophlebitis in either leg)	37 (28%)
Symptoms not attributable to knee osteoarthritis	33 (25%)
Fixed flexion deformity that prevents fitting of brace	19 (15%)
Brace size unavailable for leg circumference	2 (2%)
**Total**	**130**

Figures are numbers (percentages in brackets). The first reason on the list above to apply is coded as the reason for ineligibility as multiple reasons for ineligibility can apply during eligibility screening.

* 12 participants were initially ineligible for the study for a time-dependent reason (e.g. knee injection in the last 3-months), but re-contacted the research centre after the time-restriction had passed for re-screening, and were then eligible for the study.

† 9 participants were initially eligible for the study, but were unable to attend the clinical assessment due to clinic closure during the COVID-19 pandemic. These participants were re-screened when the research clinics re-opened and were subsequently found to be ineligible.

‡ Gout was introduced to telephone screening, in addition to face-to-face screening, 14 months into the trial to further streamline recruitment.

eTable 8: Additional baseline characteristics: randomisation strata and knee problem characteristics

	All randomised participants N=466*	AIE N = 229*	AIE+B N = 237*
Clinic site			
Manchester	113 (24%)	55 (24%)	58 (24%)
Cheshire	155 (33%)	76 (33%)	79 (33%)
Staffordshire	99 (21%)	50 (22%)	49 (21%)
North Tyneside	99 (21%)	48 (21%)	51 (22%)
Predominant compartmental distribution of knee OA based on combination of clinical assessment and radiographic presentation			
Medial tibiofemoral	153 (33%)	77 (34%)	76 (32%)
Lateral tibiofemoral	21 (5%)	10 (4%)	11 (5%)
Patellofemoral	101 (22%)	48 (21%)	53 (22%)
No clear predominant compartmental involvement	191 (41%)	94 (41%)	97 (41%)
Instability (buckling): Knee buckled at least once in the last 3-months			
No/Not sure	228 (49%)	111 (49%)	117 (49%)
Yes	237 (51%)	117 (51%)	120 (51%)
Last month, pain aching or stiffness in the knee			
No days	1 (0%)	0 (0%)	1 (0%)
Few days	11 (2%)	4 (2%)	7 (3%)
Some days	57 (12%)	26 (11%)	31 (13%)
Most days	178 (38%)	90 (39%)	88 (37%)
All days	216 (47%)	108 (47%)	108 (46%)
	Percentage denominator: participants reporting knee buckling (N= 237)	Percentage denominator: participants reporting knee buckling (N= 117)	Percentage denominator: participants reporting knee buckling (N= 120)
Knee buckling frequency last 3-months			
1 time	7 (3%)	2 (2%)	5 (4%)
2-5 times	136 (57%)	74 (63%)	62 (52%)
6-10 times	54 (23%)	24 (21%)	30 (25%)
11-24 times	24 (10%)	12 (10%)	12 (10%)
More than 24 times	10 (4%)	4 (3%)	6 (5%)
Don’t know	6 (3%)	1 (1%)	5 (4%)
Fell and hit the floor/ground after knee buckling			
Yes	41 (17%)	23 (20%)	18 (15%)
No	196 (83%)	94 (80%)	102 (85%)
Don’t know	0 (0%)	0 (0%)	0 (0%)
Activity partaken when knee buckled			
Walking	160 (68%)	86 (74%)	74 (62%)
Going up or down stairs	97 (41%)	47 (40%)	50 (42%)
Twisting or turning	90 (38%)	45 (38%)	45 (38%)
Other	38† (16%)	15 (13%)	23 (19%)
Don’t know	2 (1%)	0 (0%)	2 (2%)

Figures are numbers (percentages in brackets). Knee problem characteristics completed in reference to the knee to be treated. AIE = Advice, written information, and exercise instruction; AIE+B = Advice, written information, and exercise instruction plus knee bracing. * Baseline questionnaire data is missing for one participant, so baseline questionnaire variables are based on 465 participants with data. † Other reasons included: during exercise or a specific activity (n = 13), during transfer e.g. sit to stand, getting in and out of car/bed (n = 20), standing (n = 3), head movement (n = 1) and no reason given (n = 1).

eTable 9: Key baseline characteristics by method of recruitment

	Recruited via GP letter following retrospective electronic medical record review<br>N=229*	Recruited via advertising campaign<br>N=231*
**Demographics**		
Age (years): Mean (SD)	65 (9)	64 (9)
Female sex	110 (48%)	102 (44%)
Ethnic group		
White	223 (97%)	220 (96%)
Black – Caribbean	0 (0%)	0 (0%)
Black – African	0 (0%)	1 (0%)
Black – Other	2 (1%)	0 (0%)
Indian	1 (0%)	3 (1%)
Pakistani	0 (0%)	2 (1%)
Bangladeshi	0 (0%)	0 (0%)
Chinese	1 (0%)	2 (1%)
Prefer not to say	1 (0%)	2 (1%)
Other – stated as North African	1 (0%)	0 (0%)
Left school to attend full-time education or university	82 (36%)	112 (49%)
Currently in paid employment (full or part-time)	89 (39%)	102 (45%)
Index of multiple deprivation (IMD) (1 - 32,844):		
Mean (SD)	20480 (8360)	18797 (9478)
Median [IQR]	21866 [14313, 27277]	20961 [10219, 26599]
IMD Quintile		
1: IMD 1 to 6568	17 (7%)	38 (16%)
2: IMD 6569 to 13137	34 (15%)	33 (14%)
3: IMD 13138 to 19706	46 (20%)	33 (14%)
4: IMD 19707 to 26275	64 (28%)	62 (27%)
5: IMD 26276 to 32844	68 (30%)	65 (28%)
**General Health and Wellbeing**		
Pain in the last 4 weeks lasting for a day or longer in any part of the body	181 (83%)	195 (89%)
Bilateral knee pain †	68 (30%)	91 (40%)
Upper limb pain †	88 (38%)	84 (37%)
Lower limb pain, excluding the knee †	144 (63%)	164 (71%)
Manchester definition of widespread pain †	34 (15%)	24 (10%)
Has a long-term (>12 months) physical or mental health condition, disability or illness	106 (47%)	101 (45%)
Blindness or partial sight	8 (3%)	7 (3%)
A breathing condition e.g. asthma or COPD	27 (12%)	27 (12%)
Cancer (diagnosis or treatment in the last 5 years)	7 (3%)	6 (3%)
Deafness or hearing loss	41 (18%)	22 (10%)
Diabetes	19 (8%)	18 (8%)
Heart condition e.g. angina or atrial fibrillation	21 (9%)	16 (7%)
High blood pressure	60 (26%)	69 (30%)
Kidney or liver disease	5 (2%)	5 (2%)
A mental health condition	16 (7%)	13 (6%)
A neurological condition e.g. epilepsy	2 (1%)	5 (2%)

A stroke (which affects day-to-day life)	3 (1%)	0 (0%)
Takes more than 5 medications on a regular basis	65 (29%)	63 (28%)
Body-mass index (BMI) (kg/m^2):		
Mean (SD)	29.3 (5.9)	28.6 (5.3)
Median [IQR]	28.5 [25.3, 32.0]	27.6 [24.8, 31.1]
Categorised BMI		
Underweight: BMI <18.5 kg/m^2	0 (0%)	0 (0%)
Normal weight: BMI>=18.5kg/m^2&<24.9 kg/m^2	50 (23%)	59 (27%)
Overweight: BMI >= 24.9 kg/m^2 & < 29.9 kg/m^2	81 (37%)	85 (38%)
Obese: BMI >= 29.9 kg/m^2	90 (41%)	78 (35%)
HADS: anxiety (0-21):		
Mean (SD)	5.5 (3.9)	5.4 (4.1)
Median [IQR]	5.0 [3.0, 7.0]	5.0 [2.0, 8.0]
HADS: depression (0-21):		
Mean (SD)	4.5 (3.0)	4.5 (3.1)
Median [IQR]	4.0 [2.0, 7.0]	4.0 [2.0, 7.0]
**Stratifying variables in the randomisation process**		
Clinic site		
Manchester	30 (13%)	82 (35%)
Cheshire	67 (29%)	86 (37%)
Staffordshire	63 (28%)	35 (15%)
North Tyneside	69 (30%)	28 (12%)
Predominant compartmental distribution of knee OA based on combination of clinical assessment and radiographic presentation		
Medial tibiofemoral	79 (34%)	74 (32%)
Lateral tibiofemoral	11 (5%)	9 (4%)
Patellofemoral	48 (21%)	52 (23%)
No predominant compartmental involvement	91 (40%)	96 (42%)
Instability (buckling): Knee buckled at least once in the last 3-months		
No/Not sure	117 (51%)	107 (47%)
Yes	112 (49%)	123 (53%)
**Trial outcome measures (where measured at baseline)**		
KOOS-5 (primary outcome) (0-100): Mean (SD)	45.5 (14.4)	45.1 (13.4)
KOOS: pain (0-100): Mean (SD)	54.5 (16.1)	53.8 (15.4)
KOOS: symptoms (0-100): Mean (SD)	45.7 (13.7)	44.3 (13.1)
KOOS: Activities of daily living (0-100):		
Mean (SD)	59.3 (19.0)	60.6 (18.7)
Median [IQR]	58.8 [45.6, 74.3]	61.8 [45.6, 75.0]
KOOS: Sport/recreation (0-100): Mean (SD)		
Mean (SD)	33.1 (25.8)	32.5 (21.4)
Median [IQR]	30.0 [15.0, 50.0]	30.0 [15.0, 45.0]
KOOS: Knee related quality of life (0-100): Mean (SD)	33.8 (17.8)	33.8 (16.6)
KOOS-4: (0-100): Mean (SD)	48.3 (13.8)	48.1 (13.0)
WOMAC		
Pain (0-20): Mean (SD)	8.1 (3.6)	8.1 (3.4)
Stiffness (0-8): Mean (SD)	4.0 (1.5)	4.0 (1.4)
Function (0-68):		
Mean (SD)	27.7 (12.9)	26.8 (12.7)

Median [IQR]	28.0 [17.5, 37.0]	26.0 [17.0, 37.0]
Last 7 days, knee pain during activity in the knee (0-10): Mean (SD)	6.3 (1.7)	6.4 (1.8)
Intermittent and constant pain (ICOAP)		
Constant pain subscale (0-100):		
Mean (SD)	36.4 (26.5)	37.3 (27.0)
Median [IQR]	35.0 [10.0, 60.0]	40.0 [15.0, 60.0]
Intermittent pain subscale (0-100): Mean (SD)	48.4 (21.5)	49.9 (19.3)
Total pain scale (0-100): Mean (SD)	43.0 (22.2)	44.1 (21.3)
Arthritis self-efficacy (1-10): Mean (SD)	5.3 (2.0)	5.4 (1.9)
Physical activity (IPAQ-E) (MET minutes per week; 0–19278):		
Mean (SD)	4253 (3256)	4306 (3242)
Median [IQR]	3492 [1733, 5838]	3786 [1586, 6146]
**Radiographic characteristics**		
Kellgren-Lawrence (KL) highest grade per knee		
0	9 (4%)	7 (3%)
1	4 (2%)	2 (1%)
2	63 (28%)	59 (26%)
3	100 (44%)	112 (48%)
4	53 (23%)	51 (22%)

Figures are numbers (percentages in brackets) unless otherwise stated. Median and interquartile range also stated for skewed variables. All outcome measures completed in reference to the knee to be treated. High score indicates least deprived for IMD, more active for IPAQ-E, worse outcome for HADS, WOMAC and knee pain on activity, ICOAP, KL score, and a better outcome for KOOS and Arthritis self-efficacy. X-rays were scored for Kellgren-Lawrence grade at the end of the trial and were the highest grade given to a compartment (or compartments when there was no predominant compartmental distribution of knee OA), so may not directly align with the clinical judgment of the x-ray that was used to guide brace allocation. Intra-rater reliability of the radiographic scoring was high: intraclass correlation coefficient (ICC) = 0.94 (95% confidence interval: 0.90, 0.96) as estimated from a 2-way random effects model with absolute agreement. IQR = interquartile range; GP = General Practitioner; SD = Standard deviation; HADS = Hospital Anxiety and Depression Scale; IPAQ-E = International Physical Activity Questionnaire – Elderly; ICOAP = Intermittent & Constant Osteoarthritis Pain; KOOS = Knee Osteoarthritis Outcomes Score; MET = Metabolic Equivalent of Task; WOMAC = Western Ontario and McMaster Universities Arthritis Index

* Baseline questionnaire data is missing for one participant; data on recruitment method is missing for 6 participants

† Defined using the pain regions of the Manchester definition of widespread pain [17].

eTable 10: Key baseline characteristics by loss to follow-up

	Returned 3-month follow-up*		Returned 6-month follow-up*		Returned 12-month follow-up*	
	Yes (N = 402)	No (N=64)	Yes (N = 396)	No (N=70)	Yes (N = 375)	No (N=91)
**Demographics**						
Age (years): Mean (SD)	64 (9)	65 (11)	64 (9)	64 (11)	65 (9)	63 (11)
Female sex	183 (46%)	30 (47%)	175 (44%)	38 (54%)	170 (45%)	43 (47%)
Ethnic group						
White	388 (97%)	61 (95%)	383 (97%)	66 (94%)	360 (96%)	89 (98%)
Black – Caribbean	0 (0%)	0 (0%)	0 (0%)	0 (0%)	0 (0%)	0 (0%)
Black – African	1 (0%)	0 (0%)	1 (0%)	0 (0%)	1 (0%)	0 (0%)
Black – Other	2 (0%)	0 (0%)	2 (0%)	0 (0%)	2 (0%)	0 (0%)
Indian	3 (1%)	1 (2%)	1 (0%)	3 (4%)	2 (0%)	2 (2%)
Pakistani	0 (0%)	2 (3%)	2 (0%)	0 (0%)	2 (0%)	0 (0%)
Bangladeshi	0 (0%)	0 (0%)	0 (0%)	0 (0%)	0 (0%)	0 (0%)
Chinese	3 (1%)	0 (0%)	3 (1%)	0 (0%)	3 (1%)	0 (0%)
Prefer not to say	3 (1%)	0 (0%)	2 (0%)	1 (1%)	3 (1%)	0 (0%)
Other – stated as North African	1 (0%)	0 (0%)	1 (0%)	0 (0%)	1 (0%)	0 (0%)
Left school to attend full-time education or university	169 (42%)	26 (41%)	170 (43%)	25 (36%)	164 (44%)	31 (35%)
Currently in paid employment (full or part-time)	169 (42%)	24 (39%)	167 (43%)	26 (37%)	155 (42%)	38 (42%)
Index of multiple deprivation (IMD) (1 - 32,844):						
Mean (SD)	19725 (8974)	19362 (9266)	19853 (8855)	18670 (9822)	19922 (8852)	18659 (9600)
Median [IQR]	21376 [12134, 27159]	21282 [12311, 26248]	21376 [13134, 27107]	21467 [9540, 27571]	21395 [13737, 27107]	21331 [9789, 27159]
IMD Quintile						
1: IMD 1 to 6568	48 (12%)	8 (12%)	45 (11%)	11 (16%)	42 (11%)	14 (15%)
2: IMD 6569 to 13137	57 (14%)	10 (16%)	54 (14%)	13 (19%)	50 (13%)	17 (19%)
3: IMD 13138 to 19706	71 (18%)	9 (14%)	72 (18%)	8 (11%)	72 (19%)	8 (9%)
4: IMD 19707 to 26275	105 (26%)	21 (33%)	108 (27%)	18 (26%)	99 (26%)	27 (30%)
5: IMD 26276 to 32844	121 (30%)	16 (25%)	117 (30%)	20 (29%)	112 (30%)	25 (27%)
**General Health and Wellbeing**						
Pain in the last 4 weeks lasting for a day or longer in any part of the body	327 (86%)	54 (89%)	325 (86%)	56 (86%)	311 (86%)	70 (85%)
Bilateral knee pain †	145 (36%)	16 (25%)	139 (35%)	22 (31%)	127 (34%)	34 (37%)
Upper limb pain †	153 (38%)	20 (31%)	151 (38%)	22 (31%)	141 (38%)	32 (35%)
Lower limb pain, excluding the knee †	271 (68%)	41 (64%)	264 (67%)	48 (69%)	251 (67%)	61 (67%)
Manchester definition of widespread pain †	51 (13%)	7 (11%)	52 (13%)	6 (9%)	47 (13%)	11 (12%)
Has a long-term (>12 months) physical or mental health condition, disability or illness	187 (47%)	23 (39%)	184 (47%)	26 (39%)	172 (47%)	38 (43%)
Blindness or partial sight	14 (3%)	1 (2%)	14 (4%)	1 (1%)	14 (4%)	1 (1%)
A breathing condition e.g. asthma or COPD	48 (12%)	6 (9%)	46 (12%)	8 (11%)	44 (12%)	10 (11%)
Cancer (diagnosis or treatment in the last 5 years)	13 (3%)	1 (2%)	13 (3%)	1 (1%)	13 (3%)	1 (1%)
Deafness or hearing loss	55 (14%)	9 (14%)	57 (14%)	7 (10%)	55 (15%)	9 (10%)
Diabetes	29 (7%)	8 (12%)	27 (7%)	10 (14%)	24 (6%)	13 (14%)
Heart condition e.g. angina or atrial fibrillation	36 (9%)	1 (2%)	32 (8%)	5 (7%)	29 (8%)	8 (9%)
High blood pressure	118 (29%)	14 (22%)	114 (29%)	18 (26%)	107 (29%)	25 (27%)
Kidney or liver disease	8 (2%)	2 (3%)	10 (3%)	0 (0%)	8 (2%)	2 (2%)
A mental health condition	27 (7%)	2 (3%)	25 (6%)	4 (6%)	23 (6%)	6 (7%)

A neurological condition e.g. epilepsy	5 (1%)	2 (3%)	6 (2%)	1 (1%)	6 (2%)	1 (1%)
A stroke (which affects day-to-day life)	3 (1%)	0 (0%)	3 (1%)	0 (0%)	3 (1%)	0 (0%)
Takes more than 5 medications on a regular basis	112 (28%)	18 (29%)	113 (29%)	17 (25%)	102 (27%)	28 (31%)
Body-mass index (BMI) (kg/m^2):						
Mean (SD)	29.0 (5.6)	28.9 (5.9)	28.8 (5.4)	29.8 (6.8)	28.7 (5.3)	30.0 (6.7)
Median [IQR]	28.0 [24.9, 31.7]	28.2 [25.0, 31.2]	27.9 [24.9, 31.4]	28.3 [24.6, 32.9]	27.8 [24.9, 31.3]	28.3 [25.2, 32.9]
Categorised BMI						
Underweight: BMI <18.5 kg/m^2	0 (0%)	0 (0%)	0 (0%)	0 (0%)	0 (0%)	0 (0%)
Normal weight: BMI>=18.5kg/m^2&<24.9 kg/m^2	98 (25%)	14 (23%)	94 (25%)	18 (27%)	93 (26%)	19 (22%)
Overweight: BMI >= 24.9 kg/m^2 & < 29.9 kg/m^2	141 (36%)	27 (44%)	146 (38%)	22 (33%)	135 (37%)	33 (38%)
Obese: BMI >= 29.9 kg/m^2	148 (38%)	21 (34%)	142 (37%)	27 (40%)	134 (37%)	35 (40%)
HADS: anxiety (0-21):						
Mean (SD)	5.3 (3.9)	5.8 (4.3)	5.3 (4.0)	6.3 (3.9)	5.3 (4.0)	6.1 (3.9)
Median [IQR]	5.0 [2.0, 8.0]	5.5 [2.0, 8.5]	5.0 [2.0, 7.0]	6.0 [3.0, 9.0]	5.0 [2.0, 7.0]	5.0 [3.0, 8.0]
HADS: depression (0-21):						
Mean (SD)	4.5 (3.1)	4.7 (2.9)	4.4 (3.1)	5.3 (3.1)	4.4 (3.1)	5.1 (2.8)
Median [IQR]	4.0 [2.0, 6.0]	4.0 [2.0, 7.0]	4.0 [2.0, 6.0]	5.0 [3.0, 7.0]	4.0 [2.0, 7.0]	5.0 [3.0, 7.0]
**Stratifying variables in the randomisation process**						
Clinic site						
Manchester	97 (24%)	16 (25%)	101 (26%)	12 (17%)	94 (25%)	19 (21%)
Cheshire	133 (33%)	22 (34%)	127 (32%)	28 (40%)	120 (32%)	35 (38%)
Staffordshire	83 (21%)	16 (25%)	80 (20%)	19 (27%)	83 (22%)	16 (18%)
North Tyneside	89 (22%)	10 (16%)	88 (22%)	11 (16%)	78 (21%)	21 (23%)
Predominant compartmental distribution of knee OA based on combination of clinical assessment and radiographic presentation						
Medial tibiofemoral	131 (33%)	22 (34%)	122 (31%)	31 (44%)	124 (33%)	29 (32%)
Lateral tibiofemoral	16 (4%)	5 (8%)	17 (4%)	4 (6%)	14 (4%)	7 (8%)
Patellofemoral	90 (22%)	11 (17%)	89 (22%)	12 (17%)	87 (23%)	14 (15%)
No clear predominant compartmental involvement	165 (41%)	26 (41%)	168 (42%)	23 (33%)	150 (40%)	41 (45%)
Instability (buckling): Knee buckled at least once in the last 3-months						
No/Not sure	204 (51%)	24 (38%)	192 (49%)	36 (51%)	179 (48%)	49 (54%)
Yes	197 (49%)	40 (62%)	203 (51%)	34 (49%)	195 (52%)	42 (46%)
**Trial outcome measures (where measured at baseline)**						
KOOS-5 (primary outcome) (0-100): Mean (SD)	45.5 (13.7)	44.0 (14.6)	45.6 (13.8)	43.8 (14.4)	46.0 (13.6)	42.4 (14.6)
KOOS: pain (0-100): Mean (SD)	54.5 (15.7)	52.3 (15.9)	54.8 (15.7)	50.8 (15.6)	55.3 (15.4)	49.8 (16.4)
KOOS: symptoms (0-100): Mean (SD)	45.9 (13.3)	39.6 (12.8)	45.5 (13.1)	42.3 (14.8)	45.6 (12.8)	42.6 (15.6)
KOOS: Activities of daily living (0-100):						
Mean (SD)	60.4 (18.8)	57.2 (18.9)	60.6 (18.8)	56.6 (18.9)	61.2 (18.3)	54.8 (20.2)
Median [IQR]	61.0 [45.6, 75.0]	56.6 [45.6, 66.9]	61.8 [45.6, 75.0]	58.8 [45.6, 66.2]	61.8 [47.1, 75.0]	53.0 [39.7, 69.1]
KOOS: Sport/recreation (0-100): Mean (SD)						
Mean (SD)	32.4 (23.1)	35.4 (26.2)	32.6 (23.0)	34.1 (26.5)	33.1 (23.0)	31.4 (25.8)
Median [IQR]	30.0 [15.0, 45.0]	30.0 [15.0, 50.0]	30.0 [15.0, 45.0]	30.0 [15.0, 50.0]	30.0 [15.0, 50.0]	30.0 [10.0, 50.0]
KOOS: Knee related quality of life (0-100): Mean (SD)	34.0 (17.1)	33.2 (17.8)	34.5 (16.9)	30.3 (18.4)	35.0 (16.9)	29.0 (17.7)
KOOS-4: (0-100): Mean (SD)	48.7 (13.2)	45.6 (13.9)	48.8 (13.1)	45.0 (14.4)	49.3 (12.8)	44.1 (14.7)
WOMAC						
Pain (0-20): Mean (SD)	8.0 (3.5)	8.6 (3.5)	7.9 (3.5)	8.8 (3.5)	7.8 (3.4)	9.0 (3.8)
Stiffness (0-8): Mean (SD)	3.9 (1.5)	4.2 (1.4)	4.0 (1.4)	4.0 (1.7)	3.9 (1.4)	4.1 (1.6)
Function (0-68):						

Mean (SD)	26.9 (12.8)	29.1 (12.9)	26.8 (12.8)	29.5 (12.8)	26.4 (12.4)	30.7 (13.7)
Median [IQR]	26.5 [17.0, 37.0]	29.5 [22.5, 37.0]	26.0 [17.0, 37.0]	28.0 [23.0, 37.0]	26.0 [17.0, 36.0]	31.9 [21.0, 41.0]
Last 7 days, knee pain during activity in the knee (0-10): Mean (SD)	6.3 (1.8)	6.4 (1.7)	6.3 (1.8)	6.5 (1.7)	6.3 (1.7)	6.5 (1.8)
Intermittent and constant pain (ICOAP)						
Constant pain subscale (0-100):						
Mean (SD)	36.1 (26.9)	40.1 (26.0)	35.8 (26.6)	41.3 (27.8)	35.4 (26.6)	41.8 (27.2)
Median [IQR]	35.0 [10.0, 60.0]	40.0 [20.0, 60.0]	35.0 [10.0, 60.0]	45.0 [20.0, 65.0]	35.0 [10.0, 55.0]	45.0 [15.0, 65.0]
Intermittent pain subscale (0-100): Mean (SD)	48.7 (20.2)	51.6 (21.4)	48.1 (20.4)	54.7 (19.8)	48.1 (20.5)	53.2 (19.8)
Total pain scale (0-100): Mean (SD)	42.9 (21.6)	46.4 (22.3)	42.4 (21.6)	48.6 (22.0)	42.2 (21.5)	48.0 (22.1)
Arthritis self-efficacy (1-10): Mean (SD)	5.3 (1.9)	5.5 (2.2)	5.4 (1.9)	5.0 (2.1)	5.3 (1.9)	5.2 (2.1)
Physical activity (IPAQ-E) (MET minutes per week; 0–19278):						
Mean (SD)	4188 (3258)	4776 (3272)	4274 (3265)	4207 (3267)	4264 (3253)	4267 (3321)
Median [IQR]	3479 [1611, 5964]	4343 [2322, 6709]	3564 [1644, 5865]	4053 [1106, 6426]	3588 [1644, 5865]	3816 [1386, 6426]
**Radiographic characteristics**						
Kellgren-Lawrence (KL) highest grade per knee						
0	14 (3%)	2 (3%)	13 (3%)	3 (4%)	13 (3%)	3 (3%)
1	4 (1%)	2 (3%)	5 (1%)	1 (1%)	5 (1%)	1 (1%)
2	109 (27%)	13 (20%)	106 (27%)	16 (23%)	99 (26%)	23 (25%)
3	182 (45%)	33 (52%)	179 (45%)	36 (51%)	169 (45%)	46 (51%)
4	93 (23%)	14 (22%)	93 (23%)	14 (20%)	89 (24%)	18 (20%)

Figures are numbers (percentages in brackets) unless otherwise stated. Median and interquartile range also stated for skewed variables. All outcome measures completed in reference to the knee to be treated. High score indicates least deprived for IMD, more active for IPAQ-E, worse outcome for HADS, WOMAC and knee pain on activity, ICOAP, KL score, and a better outcome for KOOS and Arthritis self-efficacy. X-rays were scored for Kellgren-Lawrence grade at the end of the trial and were the highest grade given to a compartment (or compartments when there was no predominant compartmental distribution of knee OA), so may not directly align with the clinical judgment of the x-ray that was used to guide brace allocation. Intra-rater reliability of the radiographic scoring was high: intraclass correlation coefficient (ICC) = 0.94 (95% confidence interval: 0.90, 0.96) as estimated from a 2-way random effects model with absolute agreement. IQR = interquartile range; SD = Standard deviation; HADS = Hospital Anxiety and Depression Scale; IPAQ-E = International Physical Activity Questionnaire – Elderly; ICOAP = Intermittent & Constant Osteoarthritis Pain; KOOS = Knee Osteoarthritis Outcomes Score; MET = Metabolic Equivalent of Task; WOMAC = Western Ontario and McMaster Universities Arthritis Index.

* Baseline questionnaire data is missing for one participant, so baseline questionnaire variables are based on 465 participants with data.

† Defined using the pain regions of the Manchester definition of widespread pain [17].

eTable 11: Characteristics of participants at each stage of trial recruitment

	Eligible on the Telephone screen (N =1030)	Attended Clinical Assessment (N=615)	Eligible at Clinical Assessment (N=485)	Attended initial treatment visit (N=468)	Randomised (N=466)
Age: Mean (SD)	65 (10)	65 (9)	64 (9)	64 (9)	64 (9)
Female sex	467 (45%)	274 (45%)	222 (46%)	213 (46%)	213 (46%)
Index of multiple deprivation (IMD) (1 - 32,844):					
Mean (SD	19469 (9206)	19654 (9036)	19572 (9052)	19726 (9020)	19676 (9006)
Median (IQR)	21356 (11604, 27277)	21603 (12134, 27107)	21331 (12134, 27107)	21499 (12235, 27164)	21376 (12178, 27107)
IMD Quintile					
1: IMD 1 to 6568	119 (12%)	72 (12%)	60 (12%)	56 (12%)	56 (12%)
2: IMD 6569 to 13137	173 (17%)	92 (15%)	70 (14%)	67 (14%)	67 (14%)
3: IMD 13138 to 19706	164 (16%)	104 (17%)	82 (17%)	80 (17%)	80 (17%)
4: IMD 19707 to 26275	273 (27%)	172 (28%)	133 (27%)	126 (27%)	126 (27%)
5: IMD 26276 to 32844	296 (29%)	175 (28%)	140 (29%)	139 (30%)	137 (29%)
**Clinical judgement** on the predominant compartmental distribution of knee OA					
Medial TIB-FEM joint	*	†	167 (35%)	162 (35%)	162 (35%)
Lateral TIB-FEM joint	*	†	31 (6%)	29 (6%)	29 (6%)
Patellofemoral joint	*	†	122 (25%)	117 (25%)	116 (25%)
No predominant compartment	*	†	164 (34%)	159 (34%)	158 (34%)
Knee to be treated					
Left	*	*	*	215 (46%)	214 (46%)
Right	*	*	*	253 (54%)	252 (54%)
**Clinical judgement and radiographic presentation** on the predominant compartmental distribution of knee OA					
Medial TIB-FEM joint	*	*	*	153 (33%)	153 (33%)
Lateral TIB-FEM joint	*	*	*	21 (4%)	21 (5%)
Patellofemoral joint	*	*	*	102 (22%)	101 (22%)
No predominant compartment	*	*	*	191 (41%)	191 (41%)

Figures are numbers (percentages in brackets) unless otherwise stated. High score indicates least deprived for IMD. SD = Standard deviation. IQR = Interquartile range.

* Data not collected/applicable.

† Data collected only for eligible participants, rather than all who attended the clinical assessment, hence data not available for the entire subgroup of interest.

eTable 12: Missing data rates for the primary outcome (KOOS-5) and associated subscales

	N (%) of missing data			
	Baseline<br>N=466	3-months<br>N=402	6-months<br>N=396	12-months<br>N=375
KOOS-5 (primary outcome) (0-100)	16 (3%)	19 (5%)	15 (4%)	19 (5%)
KOOS: pain (0-100)	1 (0%)	1 (0%)	0 (0%)	2 (1%)
KOOS: symptoms (0-100)	1 (0%)	1 (0%)	0 (0%)	2 (1%)
KOOS: Activities of daily living (0-100)	2 (0%)	2 (1%)	1 (0%)	4 (1%)
KOOS: Sport/recreation (0-100)	16 (3%)	19 (5%)	15 (4%)	18 (5%)
KOOS: Knee related quality of life (0-100)	2 (0%)	1 (0%)	1 (0%)	4 (1%)

Figures are numbers (percentages in brackets). Percentages are calculated using a denominator of the number of participants returning a questionnaire at the time-point of interest; follow-up missing data rates would be higher if calculated using a denominator of all randomised participants. Missing data rates for all other secondary outcomes are less than 3% at all follow-up time-points except for the IPAQ-E physical activity measure (missing data rates for this measure ranged from 4% to 8%).

eTable 13: Missing data patterns for the primary outcome (KOOS-5)

Baseline	3-months	6-months	12-months	N (%)
Yes	Yes	Yes	Yes	306 (66%)
Yes	No	No	No	34 (7%)
Yes	Yes	Yes	No	33 (7%)
Yes	No	Yes	Yes	24 (5%)
Yes	Yes	No	No	21 (5%)
Yes	Yes	No	Yes	15 (3%)
Yes	No	Yes	No	11 (2%)
No	No	No	No	6 (1%)
Yes	No	No	Yes	6 (1%)
No	Yes	Yes	Yes	4 (1%)
No	No	Yes	No	2 (0%)
No	Yes	No	No	2 (0%)
No	Yes	No	Yes	1 (0%)
No	Yes	Yes	No	1 (0%)

The percentage of participants with 0, 1, 2, 3, and 4 KOOS-5 measures are: n=6 (1%), n=38 (8%), n=40 (9%), n=76 (16%), n=306 (66%) respectively.

eTable 14: Protocol deviations that could potentially impact on the primary and secondary outcome measures

Deviation	How many participants affected	Deviation occurred due to COVID pandemic	Deviation potentially impacts on primary and secondary data collected at the time-point			Treatment Arm
			3-months	6-months	12-months	
**Intervention delivery**						
**AIE**						
AIE treatment not delivered per a priori per protocol criteria*: participant withdrew from the trial prior to treatment delivery due to not being allocated a brace (n=1); participant already undertaking an exercise program at the gym (n=2); participant was not prescribed an exercise program (n=1); information on treatment delivery not recorded on the case report form (n=3)	7	N	Y	Y	Y	AIE
Participant issued heel raises at the initial treatment visit due to previous achilles tear and poor management.	1	N	Y	Y	Y	AIE
Participant booked into and attended a 2-week follow-up appointment despite being randomised to AIE	1	N	Y	Y	Y	AIE
**AIE+B**						
AIE+B treatment not delivered per a priori per protocol criteria†: brace not fitted due to broken skin/psoriasis on the knee (n=1); at least one Brief Motivational Interviewing technique was not used (n=1); did not attend the follow-up treatment visit (n= 10)	12	N	Y	Y	Y	AIE +B
Participants attended additional visit at clinic due to issues with their brace/ additional advice on brace fitting/ unable to receive a brace during the initial treatment session due to delayed brace supply or expected brace size not fitting	8	N	Y	Y	Y	AIE+B
SMS motivational prompt missed/ not sent at correct time/ sent from incorrect library (e.g. low adherence and not medium or high adherence library)	59‡	N	Y	Y	Y	AIE+B

AIE = Advice, written information, and exercise instruction; AIE+B = Advice, written information, and exercise instruction plus knee bracing.
* Delivered according to protocol if participants were given verbal advice and education (about OA or about things to try at home to help with symptoms), were provided written information about OA (the OA guidebook) and prescribed a knee exercise programme.
† delivered according to protocol if participants received AIE as described in * , a knee brace, at least one Brief Motivational Interviewing technique, at least one Short Message Service (SMS) motivational prompt and a follow-up treatment session (either remotely or face to face).
‡ 52 participants were not sent an SMS message between the 4th and 16th of December due to a system error

eTable 15: Clinical assessment and radiographic presentation of the predominant compartmental distribution of knee OA in the knee to be treated

	All randomised participants N=466
**Clinical Assessment**	
Medial tibiofemoral joint	162 (35%)
Lateral tibiofemoral joint	29 (6%)
Patellofemoral joint	116 (25%)
No predominant compartment	158 (34%)
**Radiographic presentation**	
No/minimal radiographic OA	31 (7%)
Medial tibiofemoral joint	170 (36%)
Lateral tibiofemoral joint	22 (5%)
Patellofemoral joint	55 (12%)
No predominant compartment	188 (40%)
**Combined judgement: clinical & radiographic presentation**	
Medial tibiofemoral joint	153 (33%)
Lateral tibiofemoral joint	21 (5%)
Patellofemoral joint	101 (22%)
No predominant compartment	191 (41%)

Figures are numbers (percentages in brackets).

eTable 16: Clinical judgement on predominant compartmental distribution of knee OA in the knee to be treated: comparing clinical judgement alone, with clinical judgement and radiographic presentation combined

	Clinical judgement combined with radiographic presentation				
**Clinical judgement alone**	Medial tibiofemoral joint	Lateral tibiofemoral joint	Patellofemoral joint	No predominant compartment	**Total**
Medial tibiofemoral joint	142	1	4	15	162
Lateral tibiofemoral joint	0	19	0	10	29
Patellofemoral joint	2	0	93	21	116
No predominant compartment	9	1	4	144	158
**Total**	153	21	101	190	465
Agreement (%)	86%				
Kappa* (95% confidence interval)	0.79 (0.74, 0.84)				

Figures are numbers, unless otherwise stated.
* Kappa statistic is unweighted and unadjusted for prevalence and bias.

eTable 17: Comparing brace allocation based on clinical judgement alone with clinical judgement combined with radiographic presentation

	Clinical judgement and radiographic presentation				
**Clinical judgment alone**	Medial unloader	Lateral unloader	Patellofemoral brace	Neutral stabilising brace	Total
Medial unloader	141	1	3	11	156
Lateral unloader	0	18	1	5	24
Patellofemoral brace	2	0	91	20	113
Neutral stabilising brace	8	2	4	145	159
Medial & Neutral	3	0	0	2	5
Medial & Patellofemoral	0	0	1	0	1
Lateral & Neutral	0	0	0	1	1
Patellofemoral & Neutral	0	0	2	4	6
Medial, Patellofemoral & Neutral	0	0	0	1	1
Total	154	21	102	189	466
**Based on data where a single brace type was selected at both time points**					
Agreement (%)			(87%)		
Kappa* (95% confidence interval)			0.82 (0.77, 0.86)		
**Based on all data: agreement is where at least one brace type was selected at both time points**					
Agreement (%)			(88%)		
Kappa* (95% confidence interval)			0.82 (0.78, 0.86)		

Figures are numbers, unless otherwise stated.
* Kappa statistic is unweighted and unadjusted for prevalence and bias.

eTable 18: Physiotherapists' confidence in judging the predominant compartmental distribution of knee OA in the knee to be treated

	All randomised participants N=466
**Clinical assessment alone**	
Not at all confident	0 (0%)
Somewhat confident	30 (7%)
Moderately confident	189 (41%)
Very confident	197 (43%)
Extremely confident	45 (10%)
**Radiographic presentation alone**	
Not at all confident	0 (0%)
Somewhat confident	8 (2%)
Moderately confident	102 (22%)
Very confident	236 (51%)
Extremely confident	120 (26%)
**Combined clinical assessment and radiographic presentation**	
Not at all confident	0 (0%)
Somewhat confident	14 (3%)
Moderately confident	104 (23%)
Very confident	241 (52%)
Extremely confident	103 (22%)

Figures are numbers (percentages in brackets).

eTable 19: Treatment delivery – AIE

Treatment delivered	Participants randomised to AIE N=226*
Provided verbal advice and education about osteoarthritis	225 (100%)
Provided verbal advice about things to try at home to help with symptoms	225 (100%)
Provided the osteoarthritis guidebook	225 (100%)
Prescribed a knee exercise programme	222 (98%)
Run through/demonstrate the exercise programme	222 (98%)
Provide the written exercise programme	223 (99%)
Other	
Provided with one or more element of motivational interviewing	91 (40%)
Explanation of radiographic findings	22 (10%)

Figures are numbers (percentages in brackets). AIE = Advice, written information, and exercise instruction.
* Data on the treatment delivery of AIE was missing for 3 participants.

eTable 20: Treatment delivery – AIE+B: initial treatment session

	Participants randomised to AIE+B N = 237 (unless otherwise stated)
**Delivery of AIE**	
Provided verbal advice and education about osteoarthritis	237 (100%)
Provided verbal advice about things to try at home to help with symptoms	237 (100%)
Provided the osteoarthritis guidebook	237 (100%)
Prescribed a knee exercise programme	237 (100%)
Run through/demonstrate the exercise programme	234 (99%)
Provide the written exercise programme	237 (100%)
Other	
Explanation of radiographic findings	7 (3%)
**Brace provision (N=235*)**	
Ossur unloader one (medial)†	76 (32%)
Ossur unloader one (lateral)‡	10 (4%)
Bioskin Q§	55 (23%)
Ossur Formfit knee hinged	94 (40%)
When the brace was issued did you:	
Contour brace hinges	12 (13%)
(denominator = those allocated a Ossur formfit knee hinged brace: N = 94)	
Cut brace straps	76 (32%)
Adjust brace 'force'	73 (85%)
(denominator = those allocated an unloader brace only: N = 86)	
Practice walking with the brace on	230 (98%)
Practice stairs with the brace on	164 (70%)
Get the participant to demonstrate taking the brace on and off	229 (97%)
Provide verbal advice on how the brace works and how to care for it	235 (100%)
Provide verbal advice on how often to wear the brace initially and how to build up use over time	235 (100%)
Provide the written brace information leaflet	234 (100%)
Address specific problems/concerns raised by participants	196 (99%¶)
On first trying the brace on in clinic, did the participant report:	
Marked reduction in knee pain	72 (31%)
Marked increase in knee pain	1 (0%)
No marked change in knee pain	162 (69%)
How satisfied were you with your brace fitting for the participant	
Not at all satisfied	0 (0%)
Somewhat satisfied	6 (3%)
Moderately satisfied	43 (18%)
Very satisfied	139 (59%)
Extremely satisfied	46 (20%)
**Motivational Interviewing (N=235*)**	
How many affirmations did you employ	
None	5 (2%)
1-2	75 (32%)
3-4	111 (48%)
5 or more	40 (17%)
How often did you employ reflective listening	

None of the time	1 (0%)
Reflected every statement made by patient	44 (19%)
For every question I asked, I gave 1-2 reflections	80 (34%)
Reflected occasionally when I felt it was necessary	109 (47%)
How often did you ask a patient an open-ended question	
None of the time	1 (0%)
For every one open ended question I asked one closed question	80 (34%)
For every two open ended questions I asked one closed question	123 (53%)
For every three open ended questions I asked one closed question	30 (13%)
How often did you elicit change talk from patients	
I did not hear any change talk	3 (1%)
I heard change talk infrequently	30 (13%)
I heard change talk sometimes	96 (41%)
I heard change talk often	105 (45%)
How often did you use summaries	
I did not use summaries	2 (1%)
I used summaries only at the beginning and end of the session	84 (36%)
I used summaries only when transitioning to another topic and at the beginning and end of the session	84 (36%)
I used summaries only when transitioning to another topic, at the beginning and end of the session, and at times when I wanted to ensure that I was understanding things correctly	64 (27%)
Did you provide a knee brace diary	230 (100%)

Figures are numbers (percentages in brackets). AIE = Advice, written information, and exercise instruction; AIE+B = Advice, written information, and exercise instruction plus knee bracing.

* Two participants were not given a brace at the initial treatment session due to broken skin/psoriasis on the knee and difficulties fitting the brace (one subsequently received the brace at the two-week follow-up appointment).

† Includes: Medial Unloader One (n=67); Medial Unloader One Short (n=3); Medial Unloader One - type not recorded (n=6).

‡ Includes: Lateral Unloader One (n=7); Lateral Unloader One Short (n=1); Lateral Unloader One - type not recorded (n=2).

§ Includes: Bioskin Q Sleeve (n=26); Bioskin Q front closing (n=25); Bioskin Q - type not recorded (n=4).

¶ 38 participants did not raise any specific problems or concerns to be addressed.

eTable 21: Treatment delivery – AIE+B: follow-up treatment session

	Participants allocated a knee brace with a follow-up visit<br>N=226<br>(unless otherwise stated)
**Brace provision**	
Brace size changed due to fit*	11 (5%)
Brace type changed due to fit†	3 (1%)
How often, and for how long, the participant had worn a knee brace was discussed	226 (100%)
Knee brace diary was reviewed	198 (90%)
(denominator = participants given a brace diary at the initial treatment visit and who attended the follow-up treatment visit: N = 221)	
Knee brace diary was completed…	
(denominator = participants whose brace diary was reviewed: N = 198))	
Not at all	5 (3%)
On a few occasions	7 (4%)
Partially	9 (5%)
Very well (omissions on a few occasions only)	175 (89%)
Did you do any of the following:	
Adjust brace fit	94 (42%)
Practice walking with the brace on	181 (80%)
Practice stairs with the brace on	119 (53%)
Get the participant to demonstrate taking the brace on and off	195 (87%)
Provide verbal advice on how the brace works and how to care for it	204 (91%)
Provide verbal advice on how often to wear the brace	213 (95%)
Provide the written brace information leaflet	122 (54%)
Address specific problems/concerns raised by participants	202 (90%)
Other – reinforced AIE	14 (6%)
**Motivational Interviewing**	
How many affirmations did you employ	
None	4 (2%)
1-2	48 (22%)
3-4	117 (53%)
5 or more	53 (24%)
How often did you employ reflective listening	
None of the time	1 (0%)
Reflected every statement made by patient	40 (18%)
For every question I asked, I gave 1-2 reflections	94 (42%)
Reflected occasionally when I felt it was necessary	87 (39%)
How often did you ask a patient an open-ended question	
None of the time	1 (0%)
For every one open ended question I asked one closed question	81 (36%)
For every two open ended questions I asked one closed question	110 (50%)
For every three open ended questions I asked one closed question	30 (14%)
How often did you elicit change talk from patients	
I did not hear any change talk	2 (1%)
I heard change talk infrequently	18 (8%)

I heard change talk sometimes	75 (33%)
I heard change talk often	129 (58%)
How often did you use summaries	
I did not use summaries	1 (0%)
I used summaries only at the beginning and end of the session	59 (26%)
I used summaries only when transitioning to another topic and at the beginning and end of the session	101 (45%)
I used summaries only when transitioning to another topic, at the beginning and end of the session, and at times when I wanted to ensure that I was understanding things correctly	63 (28%)

Figures are numbers (percentages in brackets). AIE = Advice, written information, and exercise instruction; AIE+B = Advice, written information, and exercise instruction plus knee bracing.

* Brace type remained the same, only the brace size was changed.

† Brace type changed: Bioskin Q front closing to Bioskin Q sleeve (n=1); Bioskin Q sleeve to Bioskin Q front closing (n=2).

eTable 22: Adherence to the trial treatment (AIE or AIE+B)

	3-months		6-months		12-months	
	AIE (N=229)	AIE+B (N=236)	AIE (N=229)	AIE+B (N=235)	AIE (N=226)	AIE+B (N=233)
Last X-months, followed advice and treatment from physiotherapist as often as advised						
Never	6 (3%)	3 (1%)	21 (9%)	17 (7%)	25 (11%)	10 (4%)
Rarely	10 (4%)	10 (4%)	18 (8%)	11 (5%)	13 (6%)	26 (11%)
Sometimes	49 (21%)	44 (19%)	45 (20%)	63 (27%)	55 (24%)	75 (32%)
Often	100 (44%)	116 (49%)	94 (41%)	98 (42%)	91 (40%)	79 (34%)
All of the time	62 (27%)	56 (24%)	48 (21%)	43 (18%)	39 (17%)	36 (16%)
Don't know	3 (1%)	8 (3%)	3 (1%)	2 (1%)	3 (2%)	7 (3%)

X = 3 for the 3-month and 6-month follow-up; X= 6 for the 12-month follow-up. Data based on imputed data. Variables included in the imputation model: KOOS (separate subscales), Intermittent and constant pain (ICOAP), Arthritis self-efficacy, knee pain during activity and Physical activity (IPAQ-E) (baseline, 3-, 6-, and 12-month follow-up); knee buckling, anxiety, depression (baseline only), adherence (last X months followed advice and treatment from physiotherapist) (3-, 6- and 12-month follow-up), along with age, sex, PROP-OA clinic site, and predominant compartmental distribution based on clinical and radiographic presentation. Imputed data post knee replacement excluded from analysis. AIE = Advice, written information, and exercise instruction; AIE+B = Advice, written information, and exercise instruction plus knee bracing.

eTable 23: Brace use in the AIE+B arm only

	**3-months** (N=236*)	**6-months** (N=235*)	**12-months** (N=233*)
Past 7 days, number of days worn a knee brace for more than one hour			
0	40 (17%)	60 (25%)	97 (42%)
1	5 (2%)	3 (1%)	13 (5%)
2	10 (4%)	21 (9%)	19 (8%)
3	20 (9%)	22 (9%)	15 (6%)
4	19 (8%)	16 (7%)	16 (7%)
5	37 (15%)	31 (13%)	23 (10%)
6	34 (14%)	18 (8%)	15 (6%)
7	71 (30%)	65 (28%)	35 (15%)
Mean (SD)	4.4 (2.5)	3.8 (2.7)	2.6 (2.7)
Median [IQR]	5.0 [2.9, 7.0]	4.2 [0.5, 7.0]	2.0 [0.0, 5.0]
Past 7 days, number of hours per day worn a knee brace			
Mean (SD)	4.9 (3.5)	4.5 (3.8)	3.4 (4.0)
Median [IQR]	5.0 [2.1, 7.7]	4.0 [0.4, 7.2]	2.0 [0.0, 6.0]
Past 7 days, total time spent wearing a knee brace			
Mean (SD)	28.0 (24.2)	24.1 (25.2)	16.8 (24.2)
Median [IQR]	23.1 [6.7, 42.0]	16.9 [0.0, 39.9]	3.4 [0.0, 27.6]
	N = 205†	N = 185†	N =185†
Reasons for non-brace wear			
Problems with brace fit	25 (12%)	19 (10%)	33 (18%)
Brace look and feeling self-conscious	9 (4%)	13 (7%)	9 (5%)
Can't wear some types of clothing	48 (23%)	45 (24%)	47 (25%)
Don't know how to put it on	0 (0%)	0 (0%)	1 (0%)
Knee symptoms improved	31 (15%)	31 (17%)	49 (26%)
Don't think it's doing any good	13 (6%)	21 (11%)	27 (15%)
Increased pain or other symptoms	29 (14%)	33 (18%)	28 (15%)
Too much of a hassle putting it on/off	9 (4%)	4 (2%)	15 (8%)
Uncomfortable to wear	24 (12%)	28 (15%)	33 (18%)
Lost/mislaid the brace	1 (0%)	0 (0%)	1 (0%)
Brace is damaged or worn	1 (0%)	2 (1%)	10 (5%)
Too busy/not enough time	4 (2%)	1 (0%)	4 (2%)
No longer doing the activities that require the brace	9 (4%)	3 (2%)	8 (4%)
Don't want to become reliant on it	18 (9%)	16 (9%)	19 (10%)
Forget to wear it	27 (13%)	14 (8%)	20 (11%)
Wearing it has not become a habit	22 (11%)	12 (6%)	20 (11%)
Other‡			
COVID-19 pandemic	5 (2%)	1 (0%)	2 (1%)
Lack of opportunity	2 (1%)	3 (2%)	1 (0%)
Work	3 (1%)	0 (0%)	0 (0%)
Other health conditions	3 (1%)	10 (5%)	7 (4%)
Restriction in movement	2 (1%)	3 (2%)	2 (1%)
Weather	6 (3%)	3 (2%)	1 (0%)
Change of routine	1 (0%)	1 (0%)	0 (0%)

Figures are numbers (percentages in brackets) unless otherwise stated. AIE+B = Advice, written information, and exercise instruction plus knee bracing; SD = standard deviation; IQR = interquartile range; COVID = coronavirus.
* Based on data after multiple imputation of missing data has been applied, and after imputed data collected post knee replacement is deleted. Variables included in the imputation model: Number of days and hours wearing the brace (collected by self-report questionnaires at 3-, 6- and 12-month follow-up and by text messages at 1, 2, 3, 4, 6, 8, 10, 12, 16, 20, 26 and 52-weeks post randomisation), treatment adherence (followed advice and treatment from physiotherapist) (3-, 6- and 12-month follow-up), arthritis self-efficacy, knee pain during activity (baseline, 3-, 6- and 12-months), anxiety, depression (baseline), along with age, sex, PROP-OA clinic site, and predominant compartmental distribution based on clinical and radiographic presentation and the physiotherapist that treated the participant.
† Based on responders to the question at the time-point of interest and after data collected post knee replacement is deleted. The denominator at 6-month follow-up relates to full questionnaires returned as reasons for non-brace wear were not collected on the minimum data collection form.
‡ coded from text responses that were given when participants were asked for other reasons for not wearing the brace.

eFigure 1: Graphs of total time spent wearing the knee brace in the last 7 days for each occasion of SMS text data collection

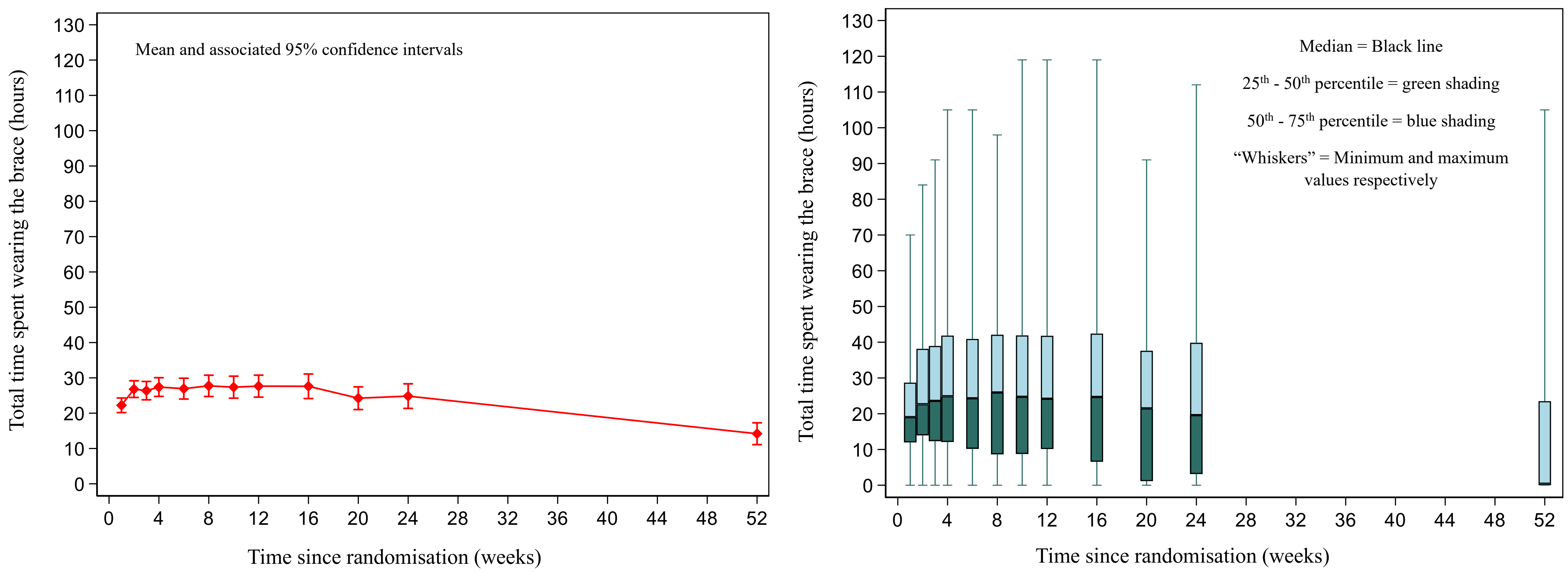

Mean (95% confidence interval), average time spent wearing the brace over 52 weeks = 25.3 (23.3, 27.3)

Median (interquartile range), average time spent wearing the brace over 52 weeks = 22.7 (13.3, 33.5)

eFigure 2: Graph of the proportion (and associated 95% confidence intervals) of those reporting they had worn the knee brace for the minimal time* for each occasion of SMS text data collection

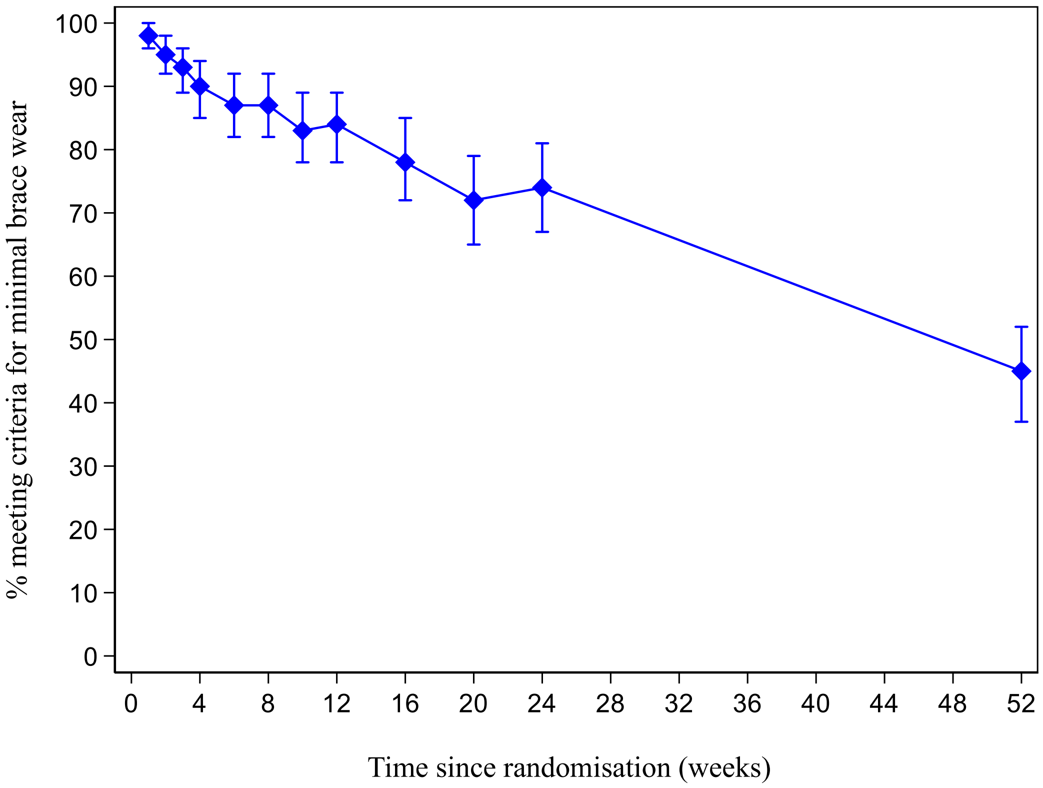

* minimal brace wear defined as if the brace is worn for 1 hour or more on two or more days of the week.

Mean (95% confidence interval), percentage of time points where criteria for minimal brace wear was met over 52 weeks = 82% (79%, 85%).

eTable 24: effect size calculations: sensitivity analyses using a range of standard deviation estimates

Outcome measure	Adjusted mean difference[*]			Standard deviation[†]				Effect size[‡]		
	3 months	6 months	12-months	Baseline	3-months	6-months	12-months	3-months	6-months	12-months
KOOS-5 (0-100)	3.67	3.39	2.67	13.87	15.43	17.17	17.97	**0.26**	**0.24**	**0.19**
								0.24	*0.20*	*0.15*
								0.20	0.19	0.15
KOOS Pain (0-100)	4.30	6.13	4.76	15.72	17.37	18.60	19.51	**0.27**	**0.39**	**0.30**
								0.25	*0.33*	*0.24*
								0.22	0.31	0.24
KOOS Symptoms (0-100)	2.97	2.15	2.01	13.39	13.27	14.17	13.52	**0.22**	**0.16**	**0.15**
								0.22	*0.15*	*0.15*
								0.21	0.15	0.14
KOOS Activities of Daily Living (0-100)	4.12	5.24	3.60	18.86	19.18	20.33	20.69	**0.22**	**0.28**	**0.19**
								0.21	*0.26*	*0.17*
								0.20	0.25	0.17
KOOS Sports/Recreation (0-100)	3.32	1.09	0.30	23.54	25.23	27.43	28.55	**0.14**	**0.05**	**0.01**
								0.13	*0.04*	*0.01*
								0.12	0.04	0.01
KOOS Quality of Life (0-100)	3.86	3.16	2.61	17.21	19.01	20.29	21.37	**0.22**	**0.18**	**0.15**
								0.20	*0.16*	*0.12*
								0.18	0.15	0.12
Knee pain on weight-bearing activity (0-10)	-0.97	-0.80	-0.72	1.76	2.03	2.17	2.39	**0.55**	**0.46**	**0.41**
								0.48	*0.37*	*0.30*
								0.41	0.34	0.30
Constant pain (ICOAP) (0-100)	-3.82	-6.53	-4.83	26.80	25.46	26.10	25.92	**0.14**	**0.24**	**0.18**
								0.15	*0.25*	*0.19*
								0.15	0.25	0.19
Intermittent pain (ICOAP) (0-100)	-5.41	-5.89	-5.44	20.42	22.29	22.83	23.84	**0.26**	**0.29**	**0.27**
								0.24	*0.26*	*0.23*
								0.23	0.25	0.23
Intermittent & Constant Pain (ICOAP) (0-100)	-4.68	-6.21	-5.25	21.75	22.27	23.28	23.77	**0.22**	**0.29**	**0.24**
								0.21	0.27	*0.22*
								0.20	0.26	0.22
Physical activity (IPAQ-E) (MET minutes per week; 0 - 19278)	601.00	760.00	264.00	3256.90	3640.00	3387.90	3432.60	**0.18**	**0.23**	**0.08**
								0.17	*0.22*	*0.08*
								0.17	0.21	0.07
Arthritis Self-Efficacy (1-10)	0.39	0.53	0.37	1.92	2.05	2.17	2.33	**0.20**	**0.28**	**0.19**
								0.19	*0.24*	*0.16*
								0.17	0.23	0.16
KOOS-4 (0-100)	3.80	4.17	3.29	13.36	14.66	15.96	16.71	**0.28**	**0.31**	**0.25**
								0.26	*0.26*	*0.20*
								0.23	0.25	0.20
WOMAC Pain (0-20)	-0.89	-1.18	-0.96	3.49	3.62	3.91	3.95	**0.25**	**0.34**	**0.27**
								0.25	*0.30*	*0.24*

								0.23	0.30	0.24
WOMAC Stiffness (0-8)	-0.14	-0.33	-0.38	1.47	1.54	1.59	1.65	**0.10**	**0.22**	**0.26**
								0.09	*0.21*	*0.23*
								0.08	0.20	0.23
WOMAC Function (0-68)	-2.80	-3.56	-2.44	12.83	13.05	13.82	14.07	**0.22**	**0.28**	**0.19**
								0.21	*0.26*	*0.17*
								0.20	0.25	0.17

High score indicates more active for IPAQ-E, worse outcome for WOMAC, knee pain on activity, ICOAP, and a better outcome for KOOS and Arthritis self-efficacy. AIE = Advice, written information, and exercise instruction; AIE+B = Advice, written information, and exercise instruction plus knee bracing; IPAQ-E = International Physical Activity Questionnaire – Elderly; ICOAP = Intermittent & Constant Osteoarthritis Pain; KOOS = Knee Osteoarthritis Outcomes Score; MET = Metabolic Equivalent of Task; WOMAC = Western Ontario and McMaster Universities Arthritis Index. Bold font = effect size calculated using the outcome standard deviation estimated at baseline; Italic font = effect size calculated using the outcome standard deviation estimated at the follow-up time-point of interest; Standard font = effect size calculated using the outcome standard deviation estimated as the largest standard deviation for the outcome across the follow-up time-points [18].

* Estimated using linear mixed models, adjusted for PROP-OA clinic site, predominant compartmental distribution, presence/absence of instability (buckling), age, sex, baseline anxiety, baseline depression, and baseline in the outcome of interest.

† Estimated as the root mean square error from an ANOVA model where the outcome is measured at the time-point of interest and predicted using a constant term and randomised treatment arm.

‡ Calculated as Cohen's effect size: Adjusted mean difference (AIE vs AIE+B)/standard deviation. Effect size estimates presented in the trial paper are calculated using the baseline standard deviation, except for pain on activity, which is (conservatively) estimated using the maximum standard deviation across the follow-up time-points. This is because, for this measure, the baseline standard deviation may be artificially attenuated given that pain on activity formed part of the eligibility criteria for the trial. [18]

eTable25: Median and interquartile range for outcome measures with a skewed distribution at follow-up

Outcome measure	3-months N = 401	6-months N=394	12-months N=370
KOOS: activities of daily living (0-100)			
AIE: Median (IQR)	63.2 (48.5, 79.4)	66.2 (48.5, 83.8)	67.6 (48.5, 85.3)
AIE+B: Median (IQR)	70.6 (54.4, 83.8)	72.1 (55.9, 86.0)	72.1 (57.4, 88.2)
KOOS: sport/recreation (0-100)			
AIE: Median (IQR)	35.0 (25.0, 55.0)	38.7 (25.0, 65.0)	40.0 (25.0, 65.0)
AIE+B: Median (IQR)	40.0 (25.0, 60.0)	40.0 (25.0, 66.7)	45.0 (25.0, 70.0)
Intermittent and constant pain (ICOAP): constant pain subscale: (0-100)			
AIE: Median (IQR)	30.0 (0.0, 50.0)	30.0 (0.0, 50.0)	25.0 (0.0, 50.0)
AIE+B: Median (IQR)	25.0 (0.0, 45.0)	25.0 (0.0, 40.0)	20.0 (0.0, 40.0)
Intermittent and constant pain (ICOAP): Intermittent pain subscale: (0-100)			
AIE: Median (IQR)	45.8 (25.0, 58.3)	37.5 (25.0, 58.3)	41.7 (18.8, 58.3)
AIE+B: Median (IQR)	35.4 (20.8, 54.2)	33.3 (20.8, 50.0)	33.3 (16.7, 50.0)
Intermittent and constant pain (ICOAP): total pain scale: (0-100)			
AIE: Median (IQR)	34.1 (15.9, 54.5)	31.8 (15.9, 54.5)	30.7 (11.4, 52.3)
AIE+B: Median (IQR)	27.3 (13.6, 47.7)	27.3 (13.6, 44.3)	25.0 (13.6, 42.6)
Physical activity (IPAQ-E): (MET minutes per week; 0-19278):			
AIE: Median (IQR)	3912 (1478, 6432)	3604 (1428, 5555)	3453 (1517, 6020)
AIE+B: Median (IQR)	4132 (1782, 6612)	3885 (1373, 6901)	3623 (1392, 6186)
WOMAC pain: (0-20)			
AIE: Median (IQR)	7.0 (4.0, 10.0)	7.0 (4.0, 10.0)	6.0 (3.0, 10.0)
AIE+B: Median (IQR)	6.0 (3.0, 9.0)	6.0 (3.0, 8.0)	5.0 (3.0, 8.0)
WOMAC function: (0-68)			
AIE: Median (IQR)	25.0 (14.0, 35.0)	23.0 (11.0, 35.0)	22.0 (10.0, 35.0)
AIE+B: Median (IQR)	20.0 (11.0, 31.0)	19.0 (9.5, 30.0)	19.0 (8.0, 29.0)

High score indicates more active for IPAQ-E, worse outcome for WOMAC and ICOAP, and a better outcome for KOOS. AIE = Advice, written information, and exercise instruction; AIE+B = Advice, written information, and exercise instruction plus knee bracing; IPAQ-E = International Physical Activity Questionnaire – Elderly; ICOAP = Intermittent & Constant Osteoarthritis Pain; IQR = Interquartile range; KOOS = Knee Osteoarthritis Outcomes Score; MET = Metabolic Equivalent of Task; WOMAC = Western Ontario and McMaster Universities Arthritis Index.

eTable 26: Intervention acceptability at 3-month follow-up

	AIE (Randomised N = 229)	AIE+B (Randomised N=237)
Acceptability of advice and treatment from the physiotherapist		
Completely unacceptable	1 (0%)	1 (0%)
Unacceptable	2 (1%)	3 (1%)
No opinion	17 (7%)	4 (2%)
Acceptable	79 (34%)	63 (27%)
Completely acceptable	96 (42%)	128 (54%)
Missing	34 (15%)	38 (16%)
Like or dislike the advice and treatment received from the physiotherapist		
Strongly dislike	0 (0%)	0 (0%)
Dislike	3 (1%)	2 (1%)
No opinion	35 (15%)	13 (5%)
Like	98 (43%)	89 (38%)
Strongly like	60 (26%)	96 (41%)
Missing	33 (14%)	37 (16%)
Effort to engage with treatment		
No effort at all	28 (12%)	26 (11%)
A little effort	77 (34%)	95 (40%)
No opinion	16 (7%)	15 (6%)
A lot of effort	61 (27%)	54 (23%)
Huge effort	11 (5%)	10 (4%)
Missing	36 (16%)	37 (16%)
There are moral or ethical consequences to engaging with the treatment and putting into practice the physiotherapists' advice		
Strongly disagree	81 (35%)	66 (28%)
Disagree	31 (14%)	44 (19%)
No opinion	58 (25%)	58 (24%)
Agree	17 (7%)	22 (9%)
Strongly agree	8 (3%)	11 (5%)
Missing	34 (15%)	36 (15%)
How fair (to all patients) is a system where all patients are offered the advice and treatment you received from the physiotherapist		
Very unfair	5 (2%)	1 (0%)
Unfair	4 (2%)	1 (0%)
No opinion	26 (11%)	26 (11%)
Fair	67 (29%)	66 (28%)
Very fair	92 (40%)	106 (45%)
Missing	35 (15%)	37 (16%)
Advice and treatment from the physiotherapist is likely to improve my knee problems in the long-term		
Strongly disagree	6 (3%)	3 (1%)
Disagree	32 (14%)	17 (7%)
No opinion	44 (19%)	31 (13%)

Agree	79 (34%)	99 (42%)
Strongly agree	32 (14%)	51 (22%)
Missing	36 (16%)	36 (15%)
It is clear how the advice and treatment from the physiotherapist would help me manage my knee problem in the long-term		
Strongly disagree	5 (2%)	1 (0%)
Disagree	21 (9%)	8 (3%)
No opinion	35 (15%)	29 (12%)
Agree	98 (43%)	103 (43%)
Strongly agree	37 (16%)	59 (25%)
Missing	33 (14%)	37 (16%)
Confidence to engage with the treatment and put into practice the advice from the physiotherapist in the long-term		
Very unconfident	2 (1%)	0 (0%)
Unconfident	29 (13%)	18 (8%)
No opinion	26 (11%)	23 (10%)
Confident	90 (39%)	90 (38%)
Very confident	48 (21%)	69 (29%)
Missing	34 (15%)	37 (16%)
Engaging with the treatment and putting into practice the physiotherapists' advice would interfere with my other priorities		
Strongly disagree	48 (21%)	45 (19%)
Disagree	87 (38%)	89 (38%)
No opinion	38 (17%)	35 (15%)
Agree	20 (9%)	28 (12%)
Strongly agree	1 (0%)	3 (1%)
Missing	35 (15%)	37 (16%)

Figures are numbers (percentages in brackets). Individual treatment acceptability questions are based on the theoretical framework of acceptability (TFA).[19] AIE = Advice, written information, and exercise instruction; AIE+B = Advice, written information, and exercise instruction plus knee bracing.

eTable 27: Complier Average Causal Effect (CACE) models for the primary outcome at 6-month follow-up (KOOS-5)

We originally planned to use structural equation modelling (SEM) and the STATA code in Troncoso et al. [20] to produce our CACE estimates but found our CACE estimates to be sensitive to inclusion of baseline predictors of outcome and adherence (see table below, particularly for adherence definition 2, where the rate of adherence was lower than definition 1). We therefore used instrumental variables (IV) analysis [21] as an alternative method to calculate our CACE estimates for comparison, along with simplifying the SEM model by constraining the model that predicted the outcome to have parameter estimates that were equal across treatment arms. This improved model fit slightly (Bayesian information Criterion (BIC) = 3263 vs 3197; 3352 vs 3292 for adherence definitions 1 and 2 respectively) and created a model that more closely resembled the model fitted using an IV approach. The estimates from the IV analysis were more stable and less sensitive to the inclusion of baseline predictors of outcome and adherence, hence are reported in the main body of the paper for the model with the lowest BIC. They are reported with caution, however, given the unstable results from the SEM approach, that this analysis deviated from the *a priori* analysis plan, and that it is challenging to define a group of participants that adhered to using the brace (yes/no) when adherence may change over time.

	Structural Equation Model		Instrumental variables	
	CACE (95% CI)	Bayesian Information Criterion (BIC)	CACE (95% CI)	Bayesian Information Criterion (BIC)
Adherence definition 1*: n (%) adherent in AIE+B: 180 (90%)				
No baseline predictors of outcome	3.50 (-0.34, 7.33)	3386	3.51 (-0.37, 7.39)	3136
Baseline predictors of outcome†	3.94 (1.16, 6.72)	3226	3.74 (0.93, 6.55)	2915
Baseline predictors of adherence‡	3.85 (-0.14, 7.84)	3419	3.19 (-0.70, 7.08)	3120
Baseline predictors of outcome† and adherence‡	3.75 (0.91, 6.58)	3263	3.87 (1.06, 6.69)	2906
Baseline predictors of outcome† and adherence‡: regression coefficients in the outcome prediction model constrained to be equal in both arms of the trial	3.82 (1.02, 6.61)	3197	N/A	N/A
Adherence definition 2§ : n (%) adherent in AIE+B: 120 (66%)				
No baseline predictors of outcome	4.66 (-0.57, 9.88)	3490	5.36 (0.18, 10.55)	3061
Baseline predictors of outcome†	3.53 (-0.09, 7.15)	3324	5.01 (1.28, 8.73)	2848
Baseline predictors of adherence‡	7.24 (1.69, 12.79)	3510	4.23 (-0.89, 9.34)	3045
Baseline predictors of outcome† and adherence‡	2.80 (-0.67, 6.26)	3352	5.21 (1.48, 8.94)	2838
Baseline predictors of outcome† and adherence‡: regression coefficients in the outcome prediction model constrained to be equal in both arms of the trial	4.18 (0.42, 7.95)	3292	N/A	N/A

AIE+B = Advice, written information, and exercise instruction plus knee bracing; CACE = Complier Average Causal Effect; CI = Confidence interval; N/A = not applicable.

* Participants defined as being adherent to treatment if they report they wore the brace for minimal time (1 hour or more on two or more days of the week) at 3-months **or** 6-months (as defined using data on the self-reported questionnaires at these time points).

† PROP-OA clinic site, predominant compartmental distribution of knee OA based on clinical and radiographic presentation, presence/absence of instability (buckling), age, sex, baseline anxiety, baseline depression, baseline KOOS-5 and baseline knee pain on weight-bearing activity.

‡ Age, sex, baseline knee pain on weight-bearing activity, predominant compartmental distribution based on clinical and radiographic presentation, anxiety, depression and self-efficacy.

§ Participants defined as being adherent to treatment if they report they wore the brace for minimal time (1 hour or more on two or more days of the week) at 3-months **and** 6-months (as defined using data on the self-reported questionnaires at these time points).

eTable 28: Exploratory subgroup analyses for the KOOS-5 primary outcome at 6-month follow-up

	Mean (SD) of KOOS-5 at 6-months	Interaction (95% CI) †
**Categorical variables**		
**Predominant knee compartment based on clinical and radiographic presentation**		
Medial tibiofemoral joint: N = 153		
AIE	46.8 (15.1)	0
AIE+B	52.1 (16.4)	
Lateral tibiofemoral joint: N = 21		
AIE	43.7 (20.2)	-2.32 (-13.77, 9.13)
AIE+B	49.9 (18.0)	
Patellofemoral joint: N = 101		
AIE	58.3 (15.6)	-4.72 (-10.43, 0.99)
AIE+B	57.9 (15.2)	
No predominant Compartment: N = 191		
AIE	53.7 (18.2)	-2.95 (-7.87, 1.98)
AIE+B	56.8 (18.1)	
**Knee buckling at baseline**		
No/not sure: N = 228		
AIE	56.3 (16.7)	0
AIE+B	59.5 (16.8)	
Yes: N = 237		
AIE	48.4 (17.1)	0.21 (-4.09, 4.50)
AIE+B	51.6 (16.5)	
**Level of adherence at 6-month follow-up***		
Never: N = 26		
AIE	53.8 (19.9)	0
AIE+B	53.7 (21.0)	
Rarely: N = 21		
AIE	58.5 (12.7)	2.97 (-13.00, 18.95)
AIE+B	64.8 (17.3)	
Sometimes: N = 83		
AIE	48.5 (17.3)	0.46 (-12.64, 13.55)
AIE+B	55.0 (14.8)	
Often: N = 150		
AIE	54.5 (16.1)	3.50 (-9.38, 16.39)
AIE+B	55.5 (17.3)	
All of the time: N = 65		
AIE	46.1 (17.8)	6.99 (-5.96, 19.95)
AIE+B	56.5 (16.5)	
Don't know: N = 2		
AIE	37.7 (‡)	‡
AIE+B	53.1 (‡)	
**Sex**		
Male: N= 253		

AIE	52.0 (16.3)	0
AIE+B	57.2 (16.0)	
Female: N=213		
AIE	52.5 (18.4)	-2.90 (-7.38, 1.58)
AIE+B	52.6 (18.3)	
**Continuous variables at baseline**		
HADS anxiety score	N/A	0.04 (-0.56, 0.64)
HADS depression score	N/A	-0.36 (-1.09, 0.37)
KOOS-5	N/A	-0.12 (-0.28, 0.03)
**Post-hoc analysis: category merging due to small N**		
**Predominant knee compartment based on clinical and radiographic presentation**		
Medial/lateral tibiofemoral joint: N = 174		
AIE	46.4 (15.7)	0
AIE+B	51.8 (16.5)	
Patellofemoral joint: N = 101		
AIE	58.3 (15.6)	-4.44 (-10.06, 1.18)
AIE+B	57.9 (15.2)	
No predominant Compartment: N = 191		
AIE	53.7 (18.2)	-2.67 (-7.52, 2.18)
AIE+B	56.8 (18.1)	
**Level of adherence at 6-month follow-up***		
Never/Rarely: N = 47		
AIE	56.0 (16.8)	0
AIE+B	57.8 (20.0)	
Sometimes: N = 83		
AIE	48.5 (17.3)	-0.36 (-10.49, 9.77)
AIE+B	55.0 (14.8)	
Often: N = 150		
AIE	54.5 (16.1)	2.69 (-7.04, 12.43)
AIE+B	55.5 (17.3)	
All of the time: N = 65		
AIE	46.1 (17.8)	6.17 (-3.74, 16.08)
AIE+B	56.5 (16.5)	
Don't know: N = 2		
AIE	37.7 (‡)	‡
AIE+B	53.1 (‡)	

High score indicates greater depression, or anxiety, for the HADS and a better outcome for KOOS-5. AIE = Advice, written information, and exercise instruction; AIE+B = Advice, written information, and exercise instruction plus knee bracing; SD = standard deviation; CI = confidence interval; N/A = not applicable; HADS = Hospital Anxiety and Depression Scale.

* Defined at 6-month follow-up using the question: In the last 3-months, have you been following the advice and treatment you received from the physiotherapist as often as you were advised to?

† Fitted using linear mixed models and adjusted for PROP-OA clinic site, predominant compartmental distribution based on clinical and radiographic presentation, presence/absence of instability (buckling), age, sex, baseline anxiety, baseline depression, baseline KOOS-5 score; the model for treatment adherence was additionally adjusted for the main effect of treatment adherence at 6-months. Ethnicity subgroup analysis not reported due to an insufficient number of non-white participants in our data sample.

‡ Unable to calculate due to only one participant in the category of interest or not calculated due to small numbers in the response category.

Appendix references

1. National Institute for Health and Care Excellence. Osteoarthritis in over-16s: diagnosis and management. NICE Guideline NG226. Royal College of Physicians, London, 2022.
2. Papalia R, Del Buono A, Osti L, Denaro V, Maffulli N. Meniscectomy as a risk factor for knee osteoarthritis: a systematic review. Br Med Bull. 2011;99:89-106. doi: 10.1093/bmb/ldq043. Epub 2011 Jan 19. PMID: 21247936.
3. van Meer BL, Meuffels DE, van Eijsden WA, Verhaar JA, Bierma-Zeinstra SM, Reijman M. Which determinants predict tibiofemoral and patellofemoral osteoarthritis after anterior cruciate ligament injury? A systematic review. Br J Sports Med. 2015 Aug;49(15):975-83. doi: 10.1136/bjsports-2013-093258. Epub 2015 Mar 30. PMID: 25824447.
4. Jones MH, Spindler KP. Risk factors for radiographic joint space narrowing and patient reported outcomes of post-traumatic osteoarthritis after ACL reconstruction: Data from the MOON cohort. J Orthop Res. 2017 Jul;35(7):1366-1374. doi: 10.1002/jor.23557. Epub 2017 Apr 28. PMID: 28383764; PMCID: PMC5497496.
5. Parsons C, Fuggle NR, Edwards MH, Goulston L, Litwic AE, Jagannath D, van der Pas S, Cooper C, Dennison EM; EPOSA Research Group. Concordance between clinical and radiographic evaluations of knee osteoarthritis. Aging Clin Exp Res. 2018 Jan;30(1):17-25. doi: 10.1007/s40520-017-0847-z. Epub 2017 Nov 3. PMID: 29101670; PMCID: PMC5785611.
6. Hinman RS, Crossley KM. Patellofemoral joint osteoarthritis: an important subgroup of knee osteoarthritis. Rheumatology (Oxford). 2007 Jul;46(7):1057-62. doi: 10.1093/rheumatology/kem114. Epub 2007 May 11. PMID: 17500072.
7. Crossley KM, Vicenzino B, Pandy MG, Schache AG, Hinman RS. Targeted physiotherapy for patellofemoral joint osteoarthritis: a protocol for a randomised, single-blind controlled trial. BMC Musculoskelet Disord. 2008 Sep 16;9:122. doi: 10.1186/1471-2474-9-122. PMID: 18793446; PMCID: PMC2556332.
8. Collins NJ, Hinman RS, Menz HB, Crossley KM. Immediate effects of foot orthoses on pain during functional tasks in people with patellofemoral osteoarthritis: A cross-over, proof-of-concept study. Knee. 2017 Jan;24(1):76-81. doi: 10.1016/j.knee.2016.09.016. Epub 2016 Nov 4. PMID: 27825592.
9. Wyndow N, Crossley KM, Vicenzino B, Tucker K, Collins NJ. A single-blinded, randomized, parallel group superiority trial investigating the effects of footwear and custom foot orthoses versus footwear alone in individuals with patellofemoral joint osteoarthritis: a phase II pilot trial protocol. J Foot Ankle Res. 2017 Apr 26;10:19. doi: 10.1186/s13047-017-0200-y. PMID: 28450898; PMCID: PMC5405497.
10. van Middelkoop M, Bennell KL, Callaghan MJ, Collins NJ, Conaghan PG, Crossley KM, Eijkenboom JJFA, van der Heijden RA, Hinman RS, Hunter DJ, Meuffels DE, Mills K, Oei EHG, Runhaar J, Schiphof D, Stefanik JJ, Bierma-Zeinstra SMA. International patellofemoral osteoarthritis consortium: Consensus statement on the diagnosis, burden, outcome measures, prognosis, risk factors and treatment. Semin Arthritis Rheum. 2018 Apr;47(5):666-675. doi: 10.1016/j.semarthrit.2017.09.009. Epub 2017 Sep 23. PMID: 29056348.
11. Stefanik JJ, Duncan R, Felson DT, Peat G. Use of Diagnostic Performance of Clinical Examination Measures and Pain Presentation to Identify Patellofemoral Joint Osteoarthritis. Arthritis Care Res (Hoboken). 2018 Jan;70(1):157-161. doi: 10.1002/acr.23238. PMID: 28320074.
12. Peat G, Duncan RC, Wood LR, Thomas E, Muller S. Clinical features of symptomatic patellofemoral joint osteoarthritis. Arthritis Res Ther. 2012 Mar 14;14(2):R63. doi: 10.1186/ar3779. PMID: 22417687; PMCID: PMC3446431.
13. Chang A, Hayes K, Dunlop D, Hurwitz D, Song J, Cahue S, Genge R, Sharma L. Thrust during ambulation and the progression of knee osteoarthritis. Arthritis Rheum. 2004 Dec;50(12):3897-903. doi: 10.1002/art.20657. PMID: 15593195.
14. Sharma L, Chang AH, Jackson RD, Nevitt M, Moisio KC, Hochberg M, Eaton C, Kwoh CK, Almagor O, Cauley J, Chmiel JS. Varus Thrust and Incident and Progressive Knee Osteoarthritis. Arthritis Rheumatol. 2017 Nov;69(11):2136-2143. doi: 10.1002/art.40224. PMID: 28772066; PMCID: PMC5659924.
15. https://www.nihr.ac.uk/research-funding/application-support/working-with-people-and-communities (accessed 10 March 2025)
16. https://sites.google.com/nihr.ac.uk/pi-standards/standards (accessed 10 March 2025)
17. MacFarlane GJ, Croft PR, Schollum J, Silman AJ. Widespread pain: is an improved classification possible? J Rheumatol. 1996 Sep;23(9):1628-32. PMID: 8877936.

18. Schnitzer TJ, Conaghan PG, Berenbaum F, Abraham L, Cappelleri JC, Bushmakin AG, Viktrup L, Yang R, Brown MT. Effect size varies based on calculation method and may affect interpretation of treatment effect: an illustration using randomised clinical trials in osteoarthritis. Adv Rheumatol. 2024 Apr 22;64(1):31. doi: 10.1186/s42358-024-00358-y. PMID: 38650049.
19. Sekhon M, Cartwright M, Francis JJ. Development of a theory-informed questionnaire to assess the acceptability of healthcare interventions. BMC Health Serv Res. 2022 Mar 1;22(1):279. doi: 10.1186/s12913-022-07577-3. PMID: 35232455; PMCID: PMC8887649.
20. Troncoso P, Ana Morales-Gómez. Estimating the complier average causal effect via a latent class approach using gsem. The Stata Journal. 2022;22(2):404-415. https://doi.org/10.1177/1536867X221106416. doi: 10.1177/1536867X221106416.
21. Baiocchi M, Cheng J, Small DS. Instrumental variable methods for causal inference. Stat Med. 2014 Jun 15;33(13):2297-340. doi: 10.1002/sim.6128. Epub 2014 Mar 6. Erratum in: Stat Med. 2014 Nov 30;33(27):4859-60. Erratum in: Stat Med. 2019 Sep 10;38(20):3960. doi: 10.1002/sim.8211. Erratum in: Stat Med. 2020 Sep 10;39(20):2693. doi: 10.1002/sim.8567. PMID: 24599889; PMCID: PMC4201653.
