# Supplementary material for: Provision of knee bracing for knee osteoarthritis (PROP OA): multicentre, parallel group, superiority, statistician blinded, randomised controlled trial
Source: BMJ. 2026 Jan 26;392:e086005. doi: 10.1136/bmj-2025-086005 (PMC12829467; doi:10.1136/bmj-2025-086005)
Supplement: Supplementary file 2 — Web appendix 2: Statistical analysis plan [file holm086005.ww2.pdf]

Data Analysis Plan (DAP)

PROvision of braces for Patients with knee OsteoArthritis trial (PROP OA):

A multi-centre, primary care, randomised, parallel-group, superiority trial (with internal pilot) to evaluate the effectiveness of bracing in the management of symptomatic knee osteoarthritis: the PROP OA trial.

Version 1.1

Date: 3rd March 2025

ISRCTN/Clinical trials.gov Number: ISRCTN28555470

This document has been written based on version 2.4. of the study protocol, dated the 8th of December 2021 (https://www.fundingawards.nihr.ac.uk/award/16/160/03). Our study protocol has also been published in BMJ Open (Holden et al. 2021)

Data Analysis Plan (DAP) revision history

Protocol version	Updated DAP version	Section number changed	Description of and reason for the change	Date changed
2.4	1.1	1.11	Social participation (via PROMIS) was an intended secondary outcome but was erroneously not included in any follow-up questionnaires. A sentence has been added to section 1.11 to explain that results for this outcome cannot be reported	03/03/2025
2.4	1.1	5.4.1	The list of variables considered as candidate predictors of the KOOS-5 at 6-month follow-up in the Complier Average Causal Effect (CACE) models should have only included measures collected pre-randomisation rather than post-randomisation. This has now been corrected by removing any variables collected post-randomisation from the list of candidate predictors	03/03/2025
2.4	1.1	Table 12.3.3	Data on maximum Kellgren-Lawrence (KL) grade by predominant compartment deleted from the table shell, as, on reflection, it is not clear what this information adds to our understanding of knee osteoarthritis in this trial, nor how to interpret the data presented.	03/03/2025
2.4	1.1	5.1.1 and 5.3	Information added on how effect sizes would be calculated for the continuous outcomes in the trial	03/03/2025

Roles and responsibilities

The undersigned have written the data analysis plan for the PROP-OA trial and agree its content:

Name	Role	Signature	Date
Dr Elaine Nicholls	Statistician	[redacted]	13/09/23
Professor Sue Jowett	Senior Health Economist	[redacted]	13/09/23
Professor Melanie Holden	Associate Investigator	[redacted]	13/09/23
Professor George Peat	Chief Investigator	[redacted]	13/09/23

The undersigned have approved the content of the analysis plan:

Name	Role	Signature	Date
Professor David Beard*	TSC Chair (on behalf of the TSC)		
Professor Catherine Hewitt*	DMC Chair (on behalf of the DMC)		

*Signatures may be in the form of an e-mail of endorsement from the TSC/DMC chair that will be printed and stored in the study master file

Elaine Nicholls and Jesse Kigozi/Zainab Abdali will undertake data cleaning and analysis of the clinical effectiveness, cost-effectiveness data respectively. Analysis of the primary clinical endpoint will be performed independently by a second statistician, who will be employed by Keele Clinical Trials Unit but not part of the study team. This will ensure the accuracy and integrity of the main study findings.

Contents

1 Introduction 8
  1.1 Background and rationale 8
  1.2 Objectives 8
  1.3 Estimands for the primary outcome at the primary end-point 9
  1.4 Context 9
  1.5 Trial design 10
  1.6 Randomisation 10
  1.7 Sample size 10
  1.8 Framework 11
  1.9 Interim analyses and stopping rules 11
  1.10 Timing of analysis 11
  1.11 Timing of outcome assessments 11
2 Statistical Principles 12
  2.1 Confidence intervals and p-values 12
  2.2 Adherence 12
  2.3 Protocol deviations 13
  2.4 Analysis populations 13
3 Trial Population 17
4 Outcome definitions 17
  4.1 Derivation rules 17
5 Analysis methods (1): clinical effectiveness analysis of AIE compared to AIE+B 26
  5.1 Primary analysis 26
    5.1.1 Statistical model 26
    5.1.2 Adjusting covariates 26
    5.1.3 Checking model assumptions 26

5.1.4 Missing data ... 27

5.1.5 Checking the imputation model ... 28

5.2 Sensitivity analyses for the primary analysis ... 29

5.2.1 Treatment effect at 6-months estimated after variation between treating therapists has been accounted for in the ANOVA model. ... 29

5.2.2 Treatment effect at 6-months estimated when data are assumed to be missing not at random. ... 29

5.2.3 Treatment effect at 6-months estimated from a longitudinal mixed model fitted directly to the data with no imputation of missing data. ... 30

5.3 Secondary analysis ... 31

5.4 Supplementary/exploratory analysis ... 32

5.4.1 Treatment effect (AIE vs AIE+B) for the primary outcome (KOOS-5) at 6-month follow-up for participants who adhered to the bracing component of AIE+B. ... 32

5.4.2 Treatment effect (AIE vs AIE+B) for the primary outcome (KOOS-5) at 6-month follow-up for participants whose clinical treatment was delivered, and data were collected, according to the study protocol. ... 33

5.4.3 Treatment effect (AIE vs AIE+B) for the primary outcome (KOOS-5) at 6-month follow-up for participants with data collected during a time-period when life-restrictions from the COVID-19 pandemic were easing ... 33

5.4.4 Treatment effect (AIE vs AIE+B) for the primary outcome (KOOS-5) at 6-month follow-up within key participant subgroups of interest ... 34

5.4.5 Longitudinal analysis of the primary outcome (KOOS-5) over time ... 36

6 Analysis methods (2): cost-effectiveness of AIE compared to AIE+B. ... 36

6.1 Health economic analysis overview ... 36

6.2 Health Outcomes ... 36

6.3 Resource use and cost collection ... 37

6.3.1 Costing of PROP-OA interventions (AIE+B and AIE) ... 37

6.3.2 Other resource utilisation ... 37

6.4 Health economic analysis ... 38

6.5 Model-based health economic analysis ... 39

7 Analysis methods (3): patients' and physiotherapists' experience of AIE and AIE+B ... 39

7.1 Safety 39
7.1.1 Serious and unexpected adverse events 39
7.1.2 Expected adverse events. 40
7.2 Acceptability and experiences of trial procedures and interventions to participants and physiotherapists 40
7.2.1 Treatment acceptability to participants 40
7.2.2 Participant adherence to trial treatment (AIE or AIE+B) 40
7.2.3 Participant barriers to use of a brace 41
7.2.4 Intervention delivery 41
7.2.5 Knee x-rays and brace allocation as delivered during the trial 41
8 Further research questions of interest 42
9 Software 42
10 Data management plan 42
11 References 42
12 Outline tables and figures 48
12.1 Clinical effectiveness paper – primary figures 48
12.2 Clinical effectiveness paper – primary tables 51
12.3 Clinical effectiveness paper – supplementary tables (part 1: clinical effectiveness analysis of AIE compared to AIE+B) 58
12.4 Tables and figures for health economics paper 67
12.5 Clinical effectiveness paper – supplementary tables (part 2: patients' and physiotherapists' experience of AIE and AIE+B) 73
13 Appendices 87
13.1 Data coding rules applied prior to analyses 87
13.2 Pre-planned adaptations to the imputation strategy 87
13.2.1 Perfect prediction 87
13.2.2 Ordinal variables 87
13.2.3 Number of nearest neighbours (k) in the predictive mean matching (PMM) models 88

13.2.4 Collinearity 88
13.2.5 Re-coding of the “Patient global rating of change” question 88
13.2.6 Dropping variables from the imputation model 88
13.2.7 Adapting the imputation model 89

1 Introduction

1.1 Background and rationale

Symptomatic knee osteoarthritis (OA) affects an estimated 10% of adults aged over 55 years and has significant impact on population health, healthcare demand and societal costs. Recommendations by the National Institute for Health and Care Excellence (NICE, 2014) suggested that people with OA, who have biomechanical joint pain, should be considered for a knee brace, however evidence was lacking on their effectiveness. The PROP-OA trial was therefore designed in response to a commissioned call from the NIHR Health Technology Assessment programme (16/160) to conduct a pragmatic randomised controlled trial (RCT), with an internal pilot phase, to investigate in primary care, the clinical and cost-effectiveness of knee braces in the management of knee osteoarthritis (OA).

1.2 Objectives

Primary objective

To determine, in adults with symptomatic knee OA, if **Advice, Written information and Exercise instruction plus Bracing with Adherence Enhancing Component (AIE+B)** is superior to **Advice, Written information and Exercise instruction (AIE)**[1] for the composite score of participant-reported pain, other symptoms, activities of daily living, function in sport and recreation and knee-related quality of life (KOOS-5) at 6 months.

Secondary objectives

1. To determine, in adults with symptomatic knee OA, if AIE+B is superior to AIE for KOOS-5 at 3 and 12 months.
2. To determine, in adults with symptomatic knee OA, if AIE+B is superior to AIE for the separate components of the KOOS-5 (patient reported pain, other symptoms, activities of daily living, function in sport and recreation and knee-related quality of life) and pain on weight-bearing activity at 3, 6 and 12 months.
3. To determine, in adults with symptomatic knee OA, the cost-effectiveness of AIE+B compared to AIE.
4. To determine, in adults with symptomatic knee OA, if AIE+B is superior to AIE for: self-reported pain; instability (buckling); treatment response; physical activity; social participation; arthritis self-efficacy.
5. To determine, in adults with symptomatic knee OA, the safety of knee bracing in adults with symptomatic knee OA ((serious) adverse events).
6. To understand the acceptability and experiences of the trial procedures and interventions (AIE and AIE+B) to participants and physiotherapists receiving and delivering the trial interventions.
7. To explore adherence to the interventions, including the barriers and enablers of adherence to brace use in participants allocated to AIE+B.
8. To determine how often clinician's judgement on the appropriate brace type is changed by plain X-ray findings.

[1] This was originally labelled 'Best Primary Care' but has been amended in light of feedback from stakeholders

9. To explore whether the effectiveness of AIE+B vs AIE depends on: (a) predominant knee OA compartmental involvement, (b) presence/absence of knee buckling, (c) level of adherence to the advice and treatment received from the physiotherapist, (d) anxiety/depression.

1.3 Estimands for the primary outcome at the primary end-point

Table 1.3.1: Estimands for the primary outcome at the primary endpoint based on the ICH E9 statistical principles for clinical trials.

**Attribute**	
Treatment	**Advice, Written information and Exercise instruction (AIE)** compared to **Advice, Written information and Exercise instruction plus Bracing with Adherence Enhancing Component (AIE+B)**, in the context of treatment delivery in the UK health service, at a time when some participants may have data collected/trial treatment during lockdown periods (March 2020 to March 2022), and where participants could freely access any health care available to them. Further details of the interventions are described in the study protocol (Holden et al. 2021).
Population	Participants defined by the inclusion and exclusion criteria of the trial as listed in Table 12.3.1 and described in the trial protocol (Holden et al. 2021)
Outcome	KOOS-5 score at 6-months follow-up
Population-level summary	Mean Difference (covariate adjusted)
Intercurrent events	Analysis population 1, predominantly focussed on a Treatment Policy approach, except for outcome data collected following knee replacement, and the event of death, which are treated using a “while with knee” and “while alive” strategy (see section 2.4 for further details)

1.4 Context

The PROP-OA trial included an internal pilot phase, with progression criteria on recruitment, intervention fidelity, adherence, and follow-up that were successfully met in September 2021 (equivalent to 9 full months of recruitment), thus the pilot phase proceeded to the full trial. We therefore aim to describe only analyses that will be completed on the full trial dataset in this analysis plan. A qualitative study was planned as part of the main trial but due to the COVID-19 pandemic, was unable to be completed. A qualitative study was completed as part of the internal pilot study but results from this will be reported in a separate publication. The PROP-OA trial recruited and followed up participants during the COVID-19 pandemic of 2020, so the impact of this will be considered in the analysis plan, and follow international guidance on reporting of trials that were impacted by the COVID pandemic (Orkin et al, 2021).

1.5 Trial design

The PROP-OA trial is a multi-centre, primary care, randomised, parallel group 2-arm superiority trial (with internal pilot). The trial is designed to test for superiority of AIE+B over AIE, hence, when treatment effectiveness is tested, the null hypothesis is of no treatment difference. The alternative hypothesis is that there is a difference in outcomes between treatment arms, hence all statistical tests are conducted 2-sided, with a 5% significance level. All results will be presented using 95% confidence intervals.

1.6 Randomisation

Participants are randomised to AIE or AIE+B with a 1:1 treatment allocation. The randomisation process is described in the study protocol, but briefly, randomisation is conducted using random permuted blocks and stratified by:

1. PROP-OA community knee pain clinic site: Staffordshire, Manchester, Cheshire and Northumbria
2. Predominant compartmental distribution of knee OA based on x-ray and clinical judgement: Medial tibiofemoral joint, Lateral tibiofemoral joint, Patellofemoral joint, No predominant compartment
3. Instability (buckling): yes, no/not sure[2]

Specification of the block sizes used, and the resulting randomisation schedule, are stored in a password protected file in the study master file, which can only be accessed by the study database developer. The randomisation schedule is determined prior to the trial commencing and according to Keele University's standard operating procedures (SOPs). When randomisation for the trial is complete, the block sizes used in the randomisation will be reported.

1.7 Sample size

Bracing trials show standardised effect sizes (ES) for short-term improvements in knee pain and function of 0.33-0.56 and 0.22-0.48 respectively for tibiofemoral unloading braces (Moyer et al. 2015) and 0.61 and 0.39 respectively for soft neoprene sleeve braces (Cudejko et al. 2018), with effect sizes varying depending on whether the control group did, or did not, use an orthosis. Our trial is powered to detect a between-group ES of 0.35 (small-to-medium effect) in primary outcome at 6 months with 2-sided 5% significance and 90% power, which, assuming a standard deviation of 23 as estimated from BEEP trial data (Foster et al. 2023), equates to a minimum clinically important difference (MCID) of 8-points on the KOOS-5; an MCID value that aligns with published evidence for the tool (Roos et al 2003). We will randomise 434 participants to allow for 20% loss to follow-up at 6 months (Hay et al. 2006, Foster et al. 2007, Foster et al. 2023), (target n at 6 months = 346; 173/arm). We have not inflated our sample size for therapist effects as each physiotherapist will be trained to deliver both interventions, however, the therapist will be included as a covariate in a sensitivity analysis of the treatment models to increase model power (Kahan et al. 2013).

[2] The knee buckling question is coded as a binary variable (yes) versus (no or not sure) as it was assumed that if a participant's knee had buckled, this would have been very noticeable, to the point that the patient would be sure it had happened.

1.8 Framework

All tests of clinical effectiveness will be based on a hypothesis of superiority. No tests of equivalence or non-inferiority will be performed.

1.9 Interim analyses and stopping rules

No interim analysis of treatment effectiveness is planned before the end of the trial.

1.10 Timing of analysis

Treatment effectiveness analyses will only be conducted after data from the last-person's 12-month questionnaire has been entered onto the study database, and after all data queries relating to the effectiveness analysis have been resolved. After verification of the primary analysis of the primary outcome by an external statistician, the data will be unblinded[3]. This will allow data that are only collected in one arm of the trial (e.g., data on brace adherence) to be fully analysed by the study statistician as even the presence of these data gives a clear indication of treatment arm allocation.

There are no plans to publish findings from the 6-month primary endpoint prior to the 12-month data being available for analysis. Consent has been sought from participants to link their trial data to Hospital Episode Statistics, National Joint Registry and medical record review for information about the receipt of knee arthroscopy and knee joint replacement. However, given that long-term follow-up is required for these data (in excess of 3-years) and that this analysis is not currently funded within the trial timeline, we do not plan to include these data in the initial results paper(s) from the trial.

1.11 Timing of outcome assessments

Primary and secondary outcomes for the clinical effectiveness analysis are in Table 1.11.1 alongside their time-points of data collection. Although social participation (via PROMIS) was an intended secondary outcome it was erroneously not included in any follow-up questionnaires, hence will not be reported in the final publication for the trial.

Table 1.11.1: Outcome measures to assess clinical effectiveness.

	Baseline	3-months	6-months	12-months
**Primary outcome**				
KOOS-5	x	x	x	x
**Key secondary outcomes**				
KOOS Activities of Daily Living	x	x	x	x
KOOS Pain	x	x	x	x
KOOS Symptoms	x	x	x	x

[3] Note that the verified analysis will not be the final analysis. This is because brace adherence data are included in the final missing data imputation model, which cannot be a blinded analysis as brace adherence data are only available in one arm of the trial. We will use the verified analysis as a sensitivity analysis, however, to explore the impact of having more auxiliary variables in the AIE+B arm of the trial, compared to the AIE arm, in the final imputed analysis.

KOOS Sports/Recreation	x	x	x	x
KOOS Quality of Life	x	x	x	x
Knee pain on weight-bearing activity (0-100 NRS)	x	x	x	x
**Additional secondary outcomes**				
KOOS-4	x	x	x	x
Intermittent & Constant Pain (ICOAP)	x	x	x	x
Knee buckling	x	x	x	x
Physical activity (IPAQ-E)	x	x	x	x
Arthritis Self-Efficacy	x	x	x	x
PROMIS Social participation	x	x	x	x
Patient global rating of change$^{\alpha}$		x	x	x
OMERACT-OARSI responder criteria		x	x	x
WOMAC	x	x	x	x

α Measure used only to classify OMERACT-OARSI responder. IPAQ-E International Physical Activity Questionnaire - Elderly; ICOAP Intermittent & Constant Osteoarthritis Pain; KOOS Knee Osteoarthritis Outcomes Score; NRS Numerical Rating Scale; OMERACT-OARSI Outcome Measures in Rheumatoid Arthritis Clinical Trials - Osteoarthritis Research Society International; WOMAC Western Ontario and McMaster Universities Arthritis Index

References for each of the outcome measures are given in the protocol. We note, however, that in the published protocol we refer to the OARSI-OMERACT responder criteria in error. This has been corrected in the analysis plan to now read OMERACT-OARSI responder criteria. Further details on how each outcome measure is scored for use in the trial is given in section 4.

2 Statistical Principles

2.1 Confidence intervals and p-values

All statistical tests will be 2-sided and tested with 5% significance i.e. presented with 95% confidence intervals. We do not plan to adjust the significance level of our pre-planned analyses to account for multiple testing as we have stated our outcomes and research hypotheses *a priori*. We note that the KOOS-4 outcome measure contains a subset of outcomes used to score the KOOS-5, but we remain that we will not adjust these analyses for multiple testing, as such analyses are clearly stated, *a priori,* in our analysis plan.

2.2 Adherence

Treatment adherence will be assessed using data from self-reported questionnaires (all participants responding at 3-, 6- and 12-month follow-up) and SMS text messaging data (brace arm only, those responding to SMS messages sent at 1, 2, 3, 4, 6, 8, 10, 12, 16, 20, 26 and 52-weeks post randomisation). Minimal brace use will be defined if the participant has worn the brace for 1 hour or

more on two or more days of the week – a definition of 'minimum regular brace use' used in a previous study (Squyer et al. 2013) and as guidance in the participant information leaflet given to participants allocated to the brace intervention.

2.3 Protocol deviations

Treatment will be deemed to have been delivered according to protocol if there is evidence of the following for each participant:

AIE

1. Provided verbal advice and education (about OA or about things to try at home to help with symptoms)
2. Provided written information about OA (the OA guidebook)
3. Prescribed a knee exercise programme

AIE+B

1. AIE provided (as described above)
2. Knee Brace provided
3. At least one Brief Motivational Interviewing technique used
4. At least one Short Message Service (SMS) motivational prompt delivered
5. The participant received a follow-up treatment session (either remotely or face to face)

2.4 Analysis populations

Our analysis populations are defined by how intercurrent events are handled in the analysis and described using the four strategies from the ICH E9 (R1) addendum on estimands: (Treatment policy, Hypothetical, While-on-treatment, and Principal stratum) (Clark et al. 2022). Details of how each strategy will be applied to our data are given in Table 2.4.1. Our analysis populations are described in Table 2.4.2.

Table 2.4.1: Strategies for handling intercurrent events and how they will be implemented in the trial data.

Strategy	Implementation in the data
Treatment policy	The value for the variable of interest will be used in the analysis regardless of whether the intercurrent event occurs
Hypothetical	Any data collected after the intercurrent event, that are affected by the intercurrent event, will be deleted in the data. Multiple imputation will then be used to impute the missing data arising from applying a hypothetical strategy
While-on-treatment	Any data collected after the intercurrent event will be deleted in the data. However, the data will remain as missing in the analysis and not imputed

Principal stratum	Participants meeting the definition for the “principal stratum” will be analysed using a complier average causal effect (CACE) analysis

Table 2.4.2: Analysis populations defined by strategy to handle intercurrent events.

Post-randomisation intercurrent events	Analysis population 1	Analysis population 2	Analysis population 3	Analysis population 4
**Protocol deviations in Table 12.3.9 that impact primary and secondary outcome data collection**				
Un-related to COVID-19 disruption	Treatment policy	Hypothetical	Treatment policy	Treatment policy
Related to COVID-19 disruption	Treatment policy	Hypothetical	Hypothetical	Treatment policy
**Data collection impacted by COVID-19 lockdown restrictions**<br>*We anticipate that all data points for the primary and secondary outcomes, the adjusting covariates of anxiety and depression, measures of adherence, and quality of life, collected between the 23rd of March 2020 (i.e. the date of the first COVID-19 lockdown) and the 23rd of March 2022 could be impacted by COVID-19 lockdown restrictions. We hypothesise this, as lockdown restrictions could have influenced participants ability to be physically active, which in turn, could influence treatment effectiveness. The latter date of the 23rd of March 2022 is arbitrary, but was chosen to mirror a time-point when restrictions were lifting and where we still had a considerable number of participants randomised after this time point to make the analysis plausible*	Treatment policy	Treatment policy	Hypothetical	Treatment policy

**Brace Adherence** *i.e. participant did not wear their brace for a sufficient length of time (see Section 5.4.1 for a definition of a sufficient length of time) in the AIE+B treatment arm for any reason, be it related, or unrelated, to the actual experience of wearing a brace*	Treatment policy	Treatment policy	Treatment policy	Principal Stratum
**Use of non-trial treatments**				
Any treatment excluding knee replacement	Treatment policy	Treatment policy	Treatment policy	Treatment policy
Knee replacement	While with natural knee joint in place in the treated (index) knee	While with natural knee joint in place in the treated (index) knee	While with natural knee joint in place in the treated (index) knee	While with natural knee joint in place in the treated (index) knee
**Adverse events**	Treatment policy	Treatment policy	Treatment policy	Treatment policy
**Death**$^{\alpha}$	While alive	While alive	While alive	While alive

We have limited data on the timing of patient's and/or physiotherapy staff being infected or treated for COVID-19, so do not use this to define our analysis populations; it is likely that such an event will happen equally in both treatment arms of the trial limiting bias in our effectiveness findings. $^{\alpha}$ We chose a 'while alive' strategy for death to avoid applying the unrealistic assumption of an immortal cohort (Wen L et al. 2017)

3 Trial Population

A CONSORT flow diagram (Schulz et al. 2010) will document the flow of participants through the study (FigureFigure 12.1.1) and will include information on the number of participants recruited and followed up, along with reasons for ineligibility or withdrawal (if given). It will also specify the timing of the withdrawal and whether the withdrawal was from treatment only, or from the trial overall. Information on the route of initial recruitment will be provided (Table 12.3.2).

Baseline characteristics of participants will be described overall (using numbers and percentages for categorical data, means and standard deviations for normally distributed continuous data and median and inter-quartile range for skewed continuous data) and by treatment arm (Table 12.2.1 and Table 12.3.3).

Table 12.2.1 will also be stratified by:

1. Method of recruitment (i.e. identification via screen of physiotherapy referrals, general practice consulters, self-referral from the community) (Table 12.3.4)
2. Data present for the primary outcome versus lost to follow-up at 3-months (Table 12.3.5)
3. Data present for the primary outcome versus lost to follow-up at 6-months (Table 12.3.5)
4. Data present for the primary outcome versus lost to follow-up at 12-months (Table 12.3.5)

The characteristics of participants included at each recruitment stage will be described (Table 12.3.4 and Table 12.3.6). For all analyses in section 3, no statistical tests will be performed to compare participant characteristics by group. Instead, the magnitude of any differences between groups will be considered and evaluated for clinical importance.

4 Outcome definitions

4.1 Derivation rules

Derivation rules used to generate the study variables are shown in Table 4.1.1. Prior to implementation of the scoring procedures, the data will be processed using the data coding rules described in our internal Standard Operating Procedure (SOP) 16 – Data Analysis – Version 5.0, which provides guidance on how to process multiple responses to a single questionnaire item and what to do if multiple questionnaires are returned for a single participant – a situation that could arise because of our reminder mailing process. We will follow this guidance and document the decision-making process for any participant as it is required using Table 13.1.1. We will store this table in the Statistical Analysis folder of the Trial Master File along with the computer syntax used to derive each of the variables to ensure we can clearly trace how the raw data are converted into the data that are analysed.

Table 4.1.1: Description of the derivation of study outcome measures and other derived measures used in the trial analysis.

Outcome measure	Scoring rule	Missing data considerations	Score interpretation	Scoring reference website (if applicable)
**Clinical Effectiveness measures**				
KOOS subscales	Subscales coded using the instructions in the scoring reference.	Missing data handled using the instructions in the scoring reference.	Range 0 – 100<br>Lower score: greater problems	KOOS User's Guide 1.1 Updated August 2012<br>http://www.koos.nu/
KOOS-5 (Primary outcome)	Average of five KOOS subscales (Pain, Symptoms, Activities of daily living, Sport/Recreation and Quality of Life)	All subscales need to be present for a score to be calculated	Range 0 – 100<br>Lower score: greater problems	KOOS User's Guide 1.1 Updated August 2012<br>http://www.koos.nu/
KOOS-4	Average of four KOOS subscales (Pain, Symptoms, Activities of daily living, and Quality of Life)	All subscales need to be present for a score to be calculated	Range 0 – 100<br>Lower score: greater problems	KOOS User's Guide 1.1 Updated August 2012<br>http://www.koos.nu/
WOMAC	Subscales calculated using the instructions in the scoring reference (converting KOOS scores to WOMAC scores)	Missing data handled using the instructions in the scoring reference.	Pain: Range 0 – 20<br>Stiffness: Range 0 – 8	KOOS User's Guide 1.1 Updated August 2012

			Function: Range 0 – 68<br><br>Lower score: lesser problems	http://www.koos.nu/
Knee pain on weight-bearing activity	Single item question so no scoring required.	All responses used for analysis	Range 0 – 10<br><br>Lower score: less pain	Not applicable
Intermittent & Constant Pain (ICOAP)	Subscales and total score coded using the instructions in the scoring reference.	Missing data handled using the instructions in the scoring reference.	Range 0 – 100<br><br>Lower score: less pain	ICOAP User's Guide Version 6: July 7, 2010.<br><br>https://oarsi.org/sites/default/files/docs/2013/icoap_users_guide_07072010.pdf
Knee buckling	Scored as "yes" vs "no or not sure"	All responses used for analysis	Binary outcome: yes/no or not sure	Not applicable
Physical activity (IPAQ-E)	Measure coded using the instructions for the IPAQ-short, however an additional scoring rule was applied to convert the IPAQ to the IPAQ-E (the IPAQ-E is the version of the IPAQ that is relevant to the elderly). This additional scoring rule was that if the question on vigorous activity was missing, it was assumed that no vigorous activity had taken place rather than this being considered as missing data (Hurtig-Wennlof et al 2010)	Missing data handled using the instructions in the scoring reference.	MET-minutes per week.<br><br>Lower score: less physically active	Guidelines for Data Processing and Analysis of the International Physical<br><br>Activity Questionnaire (IPAQ)<br><br>– Short and Long Forms.<br><br>November 2005

				www.ipaq.ki.se
Arthritis Self-Efficacy (AES-8)	Score coded using the instructions in the scoring reference.	Missing data handled using the instructions in the scoring reference.	Range 1 – 10<br><br>Lower score: less confidence to self-manage condition	Brady TJ. 2011 and<br><br>https://selfmanagementresource.com/wp-content/uploads/2022/06/English_-_self-efficacy_arthritis_8.pdf
PROMIS Social participation	Score using the T-score method described in the scoring reference	Missing data handled using the instructions in the scoring reference.	Range 27.5 – 64.2<br><br>Lower score: less ability to participate	PROMIS scoring manual: 3rd June 2022<br><br>https://www.healthmeasures.net/index.php?option=com_instruments&view=measure&id=192&Itemid=992
OMERACT-OARSI responder criteria	We will use the algorithm in the scoring reference to calculate the OMERACT-OARSI responder criteria. Our measure of pain will be the KOOS pain subscale; our measure of function will be the KOOS Activities of Daily Living subscale. The "global rating of change"	Complete data on the components of the OMERACT-OARSI responder criteria are required for the criteria to be applied (it is possible to apply the OMERACT-OARSI responder criteria to partial data, but by doing so,	Binary outcome: responder/non-responder	Pham T et al. 2004; Figure 4.

	criteria will be defined as being met if participants report that they are "Better", "Much Better" or "Completely recovered" on the global rating of change question. We will rescale the KOOS measures prior to applying the criteria so that a lower score is a better outcome. This is for ease of applying the cut-off criteria around absolute change (defined as (baseline—follow-up score) and relative change (defined as (absolute change/baseline score)).	participants with partial data are more likely to be coded as a responder than a non-responder, which could potentially introduce a bias in the data)		
**Health Economics Outcome**				
EQ-5D-5L	Scored using the Cross-walk value set (van Hout B et al 2012).	All five EQ-5D items are required to be present for an EQ-5D score to be calculated	Range -0.594 – 1<br><br>Lower score: worse quality of life	EQ-5D-5L User Guide. Version 3.0. September 2019<br><br>https://euroqol.org
**Descriptive variables**				
Age	Our aim, as far as this is possible, is to calculate "Age" for all participants who have completed the initial telephone eligibility screen, as this will represent the largest number of participants in the study and will enable us to fully explore the generalisability of the study findings.	**Date of birth**<br><br>"Date of birth" will be taken from the date of birth on Part 1 of the initial telephone eligibility assessment. However, if this information is missing, we will use other sources of data in the trial, where date of birth is recorded, to determine, where possible, what the	Range 45 years and over	Not applicable

	We will calculate "Age" as the number of years between "Date of Birth" and "Date of Part 1 of the initial telephone eligibility assessment".<br><br>For participants whose initial telephone eligibility assessment was repeated due to COVID-19 disruption, we will use the date of the repeated initial telephone assessment as the date from which to calculate "Age".<br><br>We note that when preparing the data for external data release, we will only include "Age" in the dataset, rather than "Date of Birth", to preserve participant anonymity	missing "Date of Birth" should be (e.g. the clinical eligibility assessment, the initial treatment visit and the baseline questionnaire). We will also use this process to check consistency of the recording of "Date of birth" across the different data sources. Where a lack of consistency is apparent, we will use the date of birth that is most likely to be the true date of birth across the full range of date of birth responses we have received.<br><br>**Date of Part 1 of the initial telephone eligibility assessment**<br><br>If the date of the initial telephone eligibility assessment is missing, we will impute an approximation to the true date, by considering the dates of the initial telephone eligibility forms for participants with Study identification numbers like the participant where the data are missing (the Study identification numbers are allocated sequentially so it is likely that the forms would be completed at a similar point in time)		
Sex	"Sex" will be taken from Part 1 of the Initial telephone eligibility assessment.	If information is missing for sex, we will use other sources of data in the trial, where sex is recorded, to determine,	3-level variable: Male/ Female/Other	Not applicable

		where possible, what the missing "Sex" should be (e.g. the clinical eligibility assessment, the initial treatment visit and the baseline questionnaire). We also use this process to check consistency of the recording of "Sex" across the different data sources. Where a lack of consistency is apparent, we will use the sex that is most likely to be the true sex across the full range of sex responses we have received.		
Index of multiple deprivation (IMD) 2019.	Derived from participant postcode data	All responses used for analysis	Range 1 – 32844<br><br>Lower score most deprived<br><br>Also categorised into quintiles of deprivation<br><br>1: IMD 1 to 6568<br>2: IMD 6569 to 13137<br>3: IMD 13138 to 19706<br>4: IMD 19707 to 26275<br>5: IMD 26276 to 32844<br><br>This corresponds with combining deciles of IMD into pairs i.e. deciles (1,2); (3,4); (5,6); (7,8); (9,10).	**Research report for 2019 coding:** https://assets.publishing.service.gov.uk/government/uploads/system/uploads/attachment_data/file/833947/IoD2019_Research_Report.pdf<br><br>**Scoring calculator (2019 version)** https://imd-by-postcode.opendatacommunities.org/imd/2019

Pain manikin	Coded into four binary variables: (1) bilateral knee pain, (2) upper limb pain, (3) lower limb pain, excluding the knee, (4) widespread pain using the regions defined in the Manchester definition of widespread pain	Missing data are not specifically identified on the pain manikin so does not need to be accounted for in the scoring.	Binary variables: yes/no	Macfarlane et al. 1996
HADS: Anxiety	Score coded using the instructions in the scoring reference.	No guide on how to handle missing data is provided in the tool. We will therefore use guidance in SOP16 version 5.0 that for scales with between 5 and 10 items a scale score is calculated if >= 80% of the items are present. We will calculate a HADS Anxiety Score if 6 out of the 7 items are present.	Range 0 to 21<br><br>Lower score: less anxious	Snaith RP. 2003
HADS: Depression	Score coded using the instructions in the scoring reference.	No guide on how to handle missing data is provided in the tool. We will therefore use guidance in our internal SOP16 (Data Analysis) version 5.0, that for scales with between 5 and 10 items a scale score is calculated if >= 80% of the items are present. We will calculate a HADS Depression Score if 6 out of the 7 items are present.	Range 0 to 21<br><br>Lower score: less depressed	Snaith RP. 2003
Body mass index	Calculated from weight and height on the baseline questionnaire as (Weight in kgs)/(height in meters)^2	Complete data for weight and height required	kg/m^2	Weir et al. 2022

			Also categorised as underweight/normal weight/overweight/obese according to the cut-points in the scoring reference	
**Adherence**				
Time spent wearing the brace	For each occasion where adherence data are collected (either by questionnaires or by SMS text data) calculate the total time spent wearing the brace in the last 7 days as: (number of days spent wearing a brace)*(number of hours spent wearing a brace)	Complete data on days and hours required.	Hours<br><br>Lower score: less time spent wearing the brace	Not applicable
Minimal brace wear	For each occasion where adherence data are collected (either by questionnaires or by SMS text data) define minimal brace wear if the brace is worn for 1 hour or more on two or more days of the week	Initially calculated for participants with complete data on the days and hours variables. Missing data will then be considered to explore if imputation by logic can be implemented i.e. if a participant reports that they have worn the brace for less than 2 days a week, but the time they spent wearing the brace is missing, by logic, automatically code this participant as not meeting the criteria for minimal brace wear	Binary variables: yes/no	Not applicable

Footnote: Where guidance has been given in the published tool as to how missing data should be handled this has been followed, otherwise guidance given in SOP 16 version 5.0 (Data analysis) has been used to determine the maximum number of missing items to allow in the score calculation

5 Analysis methods (1): clinical effectiveness analysis of AIE compared to AIE+B

5.1 Primary analysis

5.1.1 Statistical model

Analysis of covariance (ANCOVA) will be used to model the KOOS-5 outcome at 6-month follow-up after multiple imputation has been used to account for missing data, the data principles for analysis population 1 have been applied, and after checking that the model assumptions hold. Predictor variables in the ANOVA model will include: the baseline measure for the KOOS-5, the adjusting covariates listed in section 5.1.2, and a binary term for treatment arm.

Model results will be presented as a mean difference in outcome between treatment arms, along with associated 95% confidence intervals (p-values will only be reported at the request of journal editors when the paper is peer reviewed). We will also convert the results from the model into an effect size by dividing the mean difference (as estimated by the model) by the pooled standard deviation at baseline. The pooled standard deviation will be estimated as the root mean square error from an ANOVA model of KOOS-5 at baseline predicted using a constant term and randomised treatment arm (Schnitzer et al. 2024). To give context to the models, descriptive data (i.e. means and standard deviations) will also be reported for the KOOS-5 at 6-months by treatment arm. The results will be presented using estimates combined across the multiply imputed datasets using Rubin's rules (Table 12.2.2)

5.1.2 Adjusting covariates

Adjusting covariates will be included in the model as fixed effects. They will include the stratification variables used in the generation of the randomisation schedule i.e. PROP-OA community knee pain clinic site (Staffordshire, Cheshire, Greater Manchester and Northumbria), predominant knee OA compartmental involvement (patellofemoral, medial tibiofemoral, lateral tibiofemoral, no clear predominant compartmental involvement), presence/absence of instability (buckling) (yes versus no or not sure[4]), along with age (years), sex (male, female, other), and anxiety & depression (as measured by the HADS anxiety and depression score) (Kingsbury et al. 2016). The model that includes the adjusting covariates will be considered the primary analysis as recommended by Morris et al. 2022.

5.1.3 Checking model assumptions

Model assumptions for the ANOVA model will be checked by:

1. Plotting the residuals from the model to explore whether they follow a normal distribution.
2. Plotting the residuals against a) each independent variable in the model and b) the predicted values from the model, to ensure no pattern is observed in these plots, and that any variability in the residuals is consistent across the range of values for the independent variables.
3. Plotting the dependent variable against each predictor in the model to ensure a linear relationship is observed.

[4] The knee buckling question is coded as a binary variable (yes) versus (no or not sure) as this is the form of the variable that was used in the stratification of the randomisation.

Plot 3 will also be used to check if any outliers remain in the data after the *a priori* rules for handling implausible values detailed in the data management plan have been applied. If outliers are found, the data will be checked to ensure that data entry is accurate and then included in the analysis to reflect the pragmatic nature of the trial.

The process for checking model assumptions will be conducted just prior to final database lock so if any of the model assumptions do not hold, a change to the analysis plan can be implemented and the revised approach adopted. For example, a change to the analysis plan may be needed if the residuals from the model do not follow a normal distribution and a transformation of the dependent variable is necessary (e.g. log, square root) or if more complex generalised linear models need to be considered (e.g. those that assume a Gamma or Log-Normal distribution).

The models fitted prior to database lock will not include a term for "treatment" to ensure that the magnitude of the treatment effect is not used to guide the decision on the choice of model. Hence, if the model assumptions do not hold when the treatment term is included in the analysis, an alternative model will be used, but this will be clearly stated in any publication of the results that the model was chosen outside the *a priori* analysis plan.

5.1.4 Missing data

5.1.4.1 Descriptive statistics

The percentage of missing data will be calculated for the primary and secondary outcomes at all time-points for analysis population 1.

Missing data rates will be reported in tabular format for the KOOS-5 (primary outcome), and the subscale scores used to create it, with the latter information included to understand more fully the composition of the KOOS-5 measure (Table 12.3.7). Missing data rates for the secondary outcomes will be summarised by inclusion of an overarching sentence in the paper e.g. "Missing data rates for the secondary outcomes was less than x% at all follow-up time-points".

Patterns of missing data will be explored over time for the primary outcome (KOOS-5) with the number and percentage of participants with each pattern of missing data reported, e.g. the number of participants with the primary outcome at all follow-up time-points, at 2 out of the 3 follow-up time points etc (Table 12.3.8). Baseline characteristics of participants lost to follow-up for the primary outcome will be described at each study time point, as defined in section 3.

5.1.4.2 Multiple imputation

Multiple imputation will be used to impute missing data in the primary analysis data set.

The imputation model will include: the primary and secondary outcomes of interest at all time-points where data are collected (Table 1.11.1)[5], the adjusting variables in the regression model (Section 5.1.2), the treating therapist (to enable a sensitivity analysis to be completed), ethnicity (to enable a subgroup analysis to be completed), treatment adherence (included as a key predictor of missing data) and the EQ-5D at all time-points where data are collected (for the health economics analysis). Treatment adherence will be included in the model in multiple ways:

[5] We chose to impute the data at the level of the scale score (where applicable) rather than at the individual item level. Differences between the two approaches have been shown to be small for large sample sizes, and, by not imputing at the individual item level, we reduce the risk of convergence issues that are likely when imputing many categorical items (Rombach et al. 2018).

1. By inclusion of the question: “In the last 3-, or 6- months (deleted as appropriate depending on time point) have you been following the advice and treatment you received from the physiotherapist as often as you were advised to?” at 3-, 6- and 12-month follow-up.
2. By inclusion of questionnaire and SMS text data on “hours spent wearing a brace in the last 7 days” and whether “minimal brace wear” (as defined in Table 4.1.1) was satisfied at each time point for participants in the AIE+B arm.

As the data in (2) are only measured for participants in the AIE+B arm, we will only impute this type of adherence data for those in the AIE+B arm only. To enable treatment interactions to be included in our analysis models we will fit the imputation model separately for each arm of the trial (AIE and AIE+B) (White et al. 2011, Cro et al. 2020). Our aim is to ensure that all variables in our analysis models on imputed data are included in the imputation model (Austin et al. 2021).

The imputation model will be fitted using Multiple Imputation by chained equations (MICE), assume the data are missing at random, and will include X imputed datasets. The value of X will be defined initially to equal the percentage of participants with missing data on at least one variable in the primary regression model of interest. The resulting models for the primary and secondary outcomes at the primary endpoint will then be checked to ensure that the Monte Carlo error (MCE) estimates for all parameter estimates are <= 10% of their respective standard errors, that the MCEs for the test statistics are <=0.1 and that the MCEs for the p-values are <= 0.01. If this is not satisfied, then the number of imputations will be increased until this is achieved, and a satisfactory level of reproducibility shown (White et al. 2011). We chose to use MICE as our imputation method, rather than Multi-Variate Normal Imputation (MVNI), as MICE offers greater flexibility to form imputation models outside any known standard multivariate density function (van Buuren et al. 2007)

The imputation model will include continuous outcome measures, modelled using predictive mean matching (nearest neighbours = 10 (Morris et al. 2014)); binary outcomes, modelled using logistic regression; nominal variables, modelled as multinomial logistic regression and ordinal outcomes, modelled using ordinal regression. Predictive mean matching will be used for continuous measures as this method is suitable for the imputation of both normally distributed and skewed outcomes and produces imputed values restricted to the range of values that the measure requiring imputation can take (Morris et al. 2014).

The imputation model will be fitted to the data, however, given the complexity of the model, it may arise that the imputation model will breakdown, so it may not be possible for it to be fitted to the data. If this occurs, then the techniques described in section 13.2.7 will be explored to see how the imputation model can be adapted to ensure it can be fitted to the data. If adaptations need to be made to the imputation model, this will be explained in the results publication for the trial. If a successful imputation model can be developed, analysis models will then be fitted, and Rubin’s rules (Rubin and Schenker, 1991, Austin et al. 2021) used to combine the treatment effects and their associated standard errors across the imputed data sets. This will provide a single estimate of the treatment effect for each analysis outcome. If, however, after all other options have been considered, multiple imputation in this trial is not possible, the primary analysis will be based on a mixed-models framework.

5.1.5 Checking the imputation model

Descriptive graphs (histograms, box plots) and statistics (means, standard deviations, ranges) will be used to check that the imputed data for each variable appear theoretically plausible from what is

known about the (clinical) range of the scales in the observed data. We will also produce a boxplot of the primary outcome in the observed data and compare this to the equivalent box plot in each imputed dataset to explore whether the distribution of the primary outcome in each imputed dataset is similar, or otherwise, to the observed data (we plan to do this to increase our understanding of the impact that multiple imputation has on our dataset as if the data are missing not at random then it may not be of concern if the imputed data differ from the observed data).

5.2 Sensitivity analyses for the primary analysis

A series of sensitivity analyses will be conducted on the treatment effect (AIE versus AIE+B) for the KOOS-5 outcome measure and the results compared to the primary analysis[6]. Results of the sensitivity analyses will be presented using outline Table 12.2.2.

5.2.1 Treatment effect at 6-months estimated after variation between treating therapists has been accounted for in the ANOVA model.

The model for the primary analysis (defined in section 5.1) will be re-run but will include an additional random effect variable representing the physiotherapist who delivered the intervention. The treatment effect estimate from this revised model will be presented as a mean difference between AIE and AIE+B with the associated 95% confidence interval.

The treating physiotherapist will be defined as the physiotherapist who saw the participant at the initial treatment session, which implicitly assumes that the same physiotherapist treated the participant at the first and second treatment if randomised to the AIE+B arm (AIE consisted of one treatment session only). The number and percentage of patients that were seen by the same physiotherapist at treatment visits 1 and 2 will therefore be reported, so validity of this assumption can be determined.

For this model, there is the potential that the model will not converge if the treating physiotherapist is completely nested within "Treating Centre". If this is the case then a model dropping the term for "Treating Centre" will also be run, and the results of this latter model reported as an alternative model. To protect the identity of the therapists they will be analysed as "therapist 1", "therapist 2" etc; their identity will not be revealed in any dataset used for data sharing purposes.

5.2.2 Treatment effect at 6-months estimated when data are assumed to be missing not at random.

Our primary analysis assumes that data are "missing at random" (MAR), however, it may be that this assumption does not hold in our data set. We will therefore test how sensitive our treatment effect estimate is to this assumption using controlled imputation (Cro et al. 2020). We will use the delta method of controlled imputation applied to the primary analysis in section 5.1 (with the value of delta applied to the KOOS-5 at 6-month follow-up only)[7]. It is unlikely that we will have rich information on

---

[6] We do not plan to run sensitivity analyses to estimate the treatment effect on complete data, nor on a treatment model without any adjusting covariates. We have followed guidance in Parpia S et al. 2022 that highlight that sensitivity analyses should only be conducted when there is no evidence that one analysis method should be preferred over the other. In this instance, both analyses are inferior to the primary analysis method, so are not considered as sensitivity analyses.

[7] We chose to use the delta method to explore the impact of the MAR assumption on the treatment effect, rather than reference-based imputation, as we did not have a strong hypothesis around the symptom trajectory that participants would take after they withdrew from the trial. Also, as our primary analysis is based on multiply

the reason for withdrawal, specifically around participants who report that their reason for withdrawing from the trial is due to their knee symptoms being "much better" or "much worse", so for this reason, we will use the same value of delta irrespective of the reason for withdrawal.

We plan to use trial data to define a range of delta values to test in the data and derive this without any knowledge of treatment arm allocation. We will calculate the mean change in KOOS-5 scores between baseline and the 6-month follow-up and define a range of delta values as: 25%, 50% ,75% and 100% of the mean change as calculated. We will then review these values against our knowledge of the clinical area and our outcome of interest to see whether they represent a plausible change that could occur in a real-life setting. If they do not, e.g., if 100% of the mean change is unlikely to happen, then we will highlight this as a limitation of the analysis. We will consider both scenarios, that participants who withdraw from the trial could have better, or worse outcomes than predicted under a MAR assumption, by changing the sign of the delta coefficient in each analysis from positive to negative.

A modification of the analysis above will also be completed whereby we will assume that delta is 0 for the subgroup of participants that have at least one KOOS-5 subscale present (i.e. non-missing) in the data. Our reasoning for this is that it is likely that the KOOS-5 subscale scores will be highly correlated, hence prediction of the KOOS-5 from the other subscale scores is likely to be a good approximation to the true value of the missing data, hence a MAR assumption for this type of data may be plausible, and hence, not require a delta adjustment.

5.2.3 Treatment effect at 6-months estimated from a longitudinal mixed model fitted directly to the data with no imputation of missing data.

We chose to base our primary analysis on data after missing data had been imputed via multiple imputation, to enable a range of analysis questions to be addressed in the presence of missing data. An alternative analysis method could have been to estimate the treatment effect of interest using a longitudinal mixed model. Cro et al. (2020) highlight that these two approaches (multiple imputation and mixed models) are likely to give very similar results when the variables in the imputation model are the same as the variables in the analysis models. As we have included several auxiliary variables in our imputation procedure, there is the potential for the two analysis approaches to give differing results. We therefore plan to run a sensitivity analysis whereby the treatment effect at 6-months is estimated from a mixed model applied to non-imputed data.

A mixed model will be fitted to non-imputed KOOS-5 scores at 3-, 6- and 12-months and will include: fixed effects terms for treatment (AIE vs AIE+B), time (3-, 6- or 12-months), the interaction between treatment and time, baseline KOOS-5 score, and the adjusting covariates listed in section 5.1.2; and a random effect term for the intercept to reflect the lack of independence in the data (Twisk J et al. 2018). Time will be represented in the model as a 3-level categorical variable[8], coded as 1 = 3-months, 0 = 6-months, 2 = 12-months to enable the regression coefficient for the treatment term to be interpreted as the treatment effect at the 6-month follow-up. Separate residual terms will be fitted for

imputed data, this offered us the opportunity to test the MAR assumption by making small adaptations to that dataset, providing a controlled setting whereby the only thing that was changing between the primary analysis and the sensitivity analysis was the assumption around missing data.

[8] "Time" is represented in the model as a categorical variable as our focus is on exploring treatment effect magnitude, rather than modelling individual trajectories per-se. No *a priori* assumption is therefore be made about the shape of the trajectory over time e.g. whether it follows a linear or quadratic trajectory over time.

each follow-up time-point, however if this model fails to converge, the model will be simplified to assume a common residual across all time-points.

The mixed models will be fitted using full information maximum likelihood (FIML) estimation if the KOOS-5 score follows a normal distribution, or FIML with robust standard errors if this assumption is not met (as evaluated by visual inspection of a histogram of the KOOS-5 overall, at each time point, and stratified by treatment arm). Assumptions for the mixed model will be explored as below, and if not met, this will be reported (Singer 2003):

1. A histogram of model residuals and random intercepts (estimated using empirical Bayes estimation/best unbiased linear predictors (BLUPs)) will be produced to ensure they are normally distributed.
2. Plots of the model residuals and random intercepts against study identification number will be generated to ensure no relationship exists and to identify any specific participants with large residuals or random intercepts (i.e., to check for outliers) (the model will not be re-run excluding outliers as this is a pragmatic trial, but if large, the number of outliers will be reported).
3. Plots of the random intercepts against time-invariant predictors in the model (i.e. baseline KOOS-5, the model adjusting covariates listed in section 5.1.2 and treatment arm) and the residuals by time. No relationship should exist in these plots; they will also be used to check whether the assumption of homogeneity of variance holds for each variable in the model.
4. The covariance between the residuals and the random intercepts in the model will be inspected to ensure it is close to 0 (such covariance is assumed to be 0 in the models as fitted).

The 6-month treatment effect estimate will be presented alongside an associated 95% confidence interval (i.e. significance is tested at the 5% level) (Table 12.2.2). It is noted, however, that although the aim of this analysis was a sensitivity analysis for the treatment effect at the 6-month follow-up, with a small re-parameterisation of time in the model, treatment effects (and their 95% confidence intervals) at the 3-, and 12-month, follow-up can also be reported. Therefore, for completeness, we will report treatment effects at the 3- and 12-month follow-ups by: (1) recoding time as: 0 = 3-months; 1 = 6-months; 2 = 12-months for the 3-month treatment effect to be estimated; and (2) recoding time as: 2 = 3-months; 1 = 6-months; 0 = 12-months for the 12-month treatment effect to be estimated (Table 12.2.3).

5.3 Secondary analysis

Treatment effectiveness for the KOOS-5 at 3- and 12-months, the separate subscales of the KOOS-5 and the other secondary outcome measures at all follow-up time points will use the same overall method of analysis as for the primary outcome (Section 5.1), however, when reporting the effect size for pain on activity, we will use the maximum standard deviation across the follow-up time-points (the baseline standard deviation for this measure may be artificially attenuated given that pain on activity formed part of the eligibility criteria for the trial (Schnitzer et al. 2024)). In addition, knee buckling and the OMERACT-OARSI responder criteria are measured on a binary scale so will be modelled using logistic regression rather than ANOVA, with results presented as numbers, percentages, and odds ratios with 95% confidence intervals[9]. All models will be adjusted for the baseline measure that corresponds to the outcome of interest (e.g. if the KOOS Pain score is being modelled at 3-months, the

[9] The patient global rating of change is only used to calculate the OMERACT-OARSI responder criteria so this will not be included in the list of secondary outcomes.

model will be adjusted for the baseline KOOS Pain score). The exception to this is the OMERACT-OARSI responder criteria, as, due to its derivation, a baseline measure for this outcome cannot be calculated.

Model assumptions for the logistic regression models will explore if there is a linear relationship between the log odds and each independent variable in the model. A plot of the residuals will also be used to identify any outliers in the data, but such outliers will remain included in the analysis to reflect the pragmatic nature of the trial. Results of the secondary analysis will be presented using outline Table 12.2.3.

5.4 Supplementary/exploratory analysis

5.4.1 Treatment effect (AIE vs AIE+B) for the primary outcome (KOOS-5) at 6-month follow-up for participants who adhered to the bracing component of AIE+B.

A principal stratum approach will be used for this analysis based on analysis population 4.

Complier average causal effect (CACE) models will be fitted to the data to estimate the difference in KOOS-5 scores at 6-months between participants who adhered to the brace use component of AIE+B intervention, and those participants who would have adhered to the brace use component of AIE+B intervention if they had been randomised to AIE+B[10].

CACE models will be fitted to the data based on two different approaches to defining those participants randomised to AIE+B that have adhered to treatment (with approaches varying by how stringent the definition of adherence is):

1. Participant defined as being adherent to treatment if they report they wore the brace for minimal time at 3-months **or** 6-months (as defined using data on the self-reported questionnaires at these time points[11])
2. Participant defined as being adherent to treatment if they report they wore the brace for minimal time at 3-months **and** 6-months (as defined using data on the self-reported questionnaires at these time points)

The CACE models will be fitted using the gsem procedure in STATA (Troncoso et al. 2022) and will initially be fitted with no predictors of the outcome of interest, and no predictors of adherence. We will then explore whether model fit (as measured using Akaike's information criterion (AIC) and the Bayesian information criterion (BIC)) improves when such predictors are added to the model:

Candidate predictors of the KOOS-5 at 6-month follow-up: baseline KOOS-5, baseline knee pain on weight-bearing activity, and the adjusting covariates listed in section 5.1.2.

Candidate predictors of adherence: Current evidence on predictors of adherence in osteoarthritis is limited and contradictory (Duong et al. 2022). We will therefore select a small number of potential demographic, socioeconomic, psychosocial, condition-specific, or treatment-specific based on expert opinion.

[10] We chose a CACE model for this analysis as information on the use of the brace is only available in one arm on the trial.
[11] We have based our definition of adherence on the questionnaire data as we know from our internal pilot study that the questionnaire data on brace use has less missing data than the SMS text message data.

Currently, we have found little guidance in the literature on how to fit gsem models to data after multiple imputation has been applied, hence, our analysis will be applied to data prior to multiple imputation being performed to impute the missing data.

We will report the results from the models with the lowest AIC and BIC values to represent the models that are the best fit to the data (Table 12.2.2). When reporting our findings from this model we will be clear to highlight a key limitation of this analysis; that being the challenge of defining a group of participants that adhered to using the brace (yes/no) when adherence may change over time. We have attempted to define this in our dataset but acknowledge that other definitions could lead to different results and conclusions, therefore the findings from this analysis will remain exploratory.

5.4.2 Treatment effect (AIE vs AIE+B) for the primary outcome (KOOS-5) at 6-month follow-up for participants whose clinical treatment was delivered, and data were collected, according to the study protocol.

A list of all protocol deviations will be produced by arm, and each will be judged according to: (1) whether they are a major or minor deviation, (2) whether the deviation was related or unrelated to disruption caused by the COVID-19 pandemic and (3) whether the deviation is likely to affect responses given to the primary outcome (Table 12.3.9). Within this table, the number, and percentage of participants that meet our *a priori* definition of being treated per protocol will be reported (section 2.3)[12].

A hypothetical estimand will be constructed to estimate the KOOS-5 treatment effect at 6-month follow-up for participants that have received treatment and whose data were collected according to the trial protocol. Analysis methods described in section 5.1 will be applied to analysis population 2 and the KOOS-5 outcome data collected at the 6-month follow-up. Treatment effect estimates derived at each time-point will be reported, alongside their associated 95% confidence intervals (Table 12.2.2).

5.4.3 Treatment effect (AIE vs AIE+B) for the primary outcome (KOOS-5) at 6-month follow-up for participants with data collected during a time-period when life-restrictions from the COVID-19 pandemic were easing

We have recruited participants to the trial before, during, and after the COVID-19 pandemic, hence, our treatment effect of interest is in the context of a world where a COVID-19 outbreak started during the trial and where participants can suffer from COVID-19 infections (Van Lancker K et al. 2023, Cro S et al. 2020). We hypothesise, however, that in our clinical context, due to randomisation, the impact of the COVID-19 pandemic is likely to have affected both arms of the trial in equal measure e.g. there is little reason to suggest that administrative changes due to COVID-19, changes in treatment delivery, restricted access to services, the number of participants suffering from COVID-19 infections and participants' experience of lockdown restrictions on physical activity, social participation and social and physical mental well-being, would be different between the trial arms. Therefore, we hypothesise that our treatment effect of interest i.e. the difference in outcome between the trial arms will be less impacted (biased) by the COVID-19 pandemic than for other trial contexts where there is clear evidence that the COVID-19 pandemic disproportionately affects one arm of the trial more than the other.

---

[12] Note that no formal statistical testing will be undertaken to test whether the proportion of participants treated per protocol differs by treatment arm.

We do, however, acknowledge that a large proportion of our participants will have been recruited and followed up during the COVID-19 pandemic, hence we aim to develop a hypothetical estimand to estimate the treatment effect under the hypothetical scenario that all participant data is collected in a world where COVID-19 naturally exists, but where life restrictions are much reduced from what they were at the height of the pandemic, and where individuals can suffer from COVID-19 infections (Van Lancker K et al. 2023) (see analysis population 3 in Table 2.4.2). We are particularly interested in this estimand, as although the impact of COVID-19 is likely to have affected both arms of the trial equally (and hence have less impact when applying a hypothesis test to test for a difference in outcome between the trial arms), we do not know how the COVID-19 pandemic has affected our estimate of the magnitude of the change in outcome between baseline and follow-up (i.e. the estimate of the effect of treatment).

We do not aim to try to estimate the treatment effect in a world where COVID-19 does not exist (Van Lancker K et al. 2023), as this is unlikely to be a true scenario going forward in the world, and this analysis would also be limited by the small number of participants we have in the trial with outcome data collected prior to the start of the pandemic.

We will therefore use analysis population 3 to define our hypothetical estimand, and will re-run the analysis in section 5.1 at the 6-month follow-up time-point on this additional imputed data set, whereby treatment effect estimates and associated 95% confidence intervals will be reported (Table 12.2.2). We anticipate, however, that there may be challenges with this analysis, that the model for multiple imputation may not converge, as, potentially, a large proportion of randomised participants, will provide minimal data for analysis (e.g. there may be several participants in this analysis with no baseline or follow-up data for any of the primary and secondary outcomes, whose imputed values will be based solely on limited information known not be affected by the COVID-19 pandemic e.g. the participant's age, sex, treatment arm, clinic site or treating therapist). We have, however, included this analysis in our analysis plan as it has been shown that multiple imputation is possible in data sets with a large proportion of missing data (Madley-Dowd P et al. 2019).

5.4.4 Treatment effect (AIE vs AIE+B) for the primary outcome (KOOS-5) at 6-month follow-up within key participant subgroups of interest

Exploratory subgroup analyses will be performed for the primary outcome (KOOS-5) at the 6-month follow-up to test whether the magnitude of the treatment effect depends on the subgrouping variable of interest. The sub-grouping variables proposed in our published protocol are below:

1. Predominant knee OA compartmental involvement based on x-ray and clinical judgement: Medial tibiofemoral joint, Lateral tibiofemoral joint, Patellofemoral joint, No predominant compartment
2. Baseline knee instability (buckling): yes, no/not sure[13]
3. Baseline level of anxiety (as measured by the HADS anxiety score)
4. Baseline level of depression (as measured by the HADS depression score)
5. Level of adherence collected at 6-months: defined using the question: In the last 3-months, have you been following the advice and treatment you received from the

[13] The knee buckling question is coded as a binary variable (yes) versus (no or not sure) to be consistent with the format used in the stratification of the randomisation. This format also has clinical interpretability as it is unlikely that true knee buckling has occurred if the participant is not sure whether it has happened.

physiotherapist as often as you were advised to?: Never, Rarely, Sometimes, Often, All of the time, Don't know

Although we hypothesise that all our listed subgrouping variables could influence the magnitude of the treatment effect, for some of the subgrouping variables, we do not have a clear prediction on the exact direction of the interaction effect given the exploratory nature of the analysis i.e., there could be a reasonable justification to explain either way why the treatment effect is larger in one group compared to the other. We do, however, hypothesise *a priori* that the treatment effect will be larger in those with lower levels of depression compared to those with higher levels of depression, and larger in participants with higher levels of intervention adherence than those with lower levels of intervention adherence. We also hypothesise that the strength of the interaction effect will be greater for depression than it would be for anxiety.

In addition to the variables above, we will also consider three further subgrouping variables:

1. Baseline level of knee symptom severity (as measured by the KOOS-5 score)
2. Sex
3. Ethnicity

These further subgroup analyses have been specified after our study protocol has been published, but before any analysis has been completed on the final trial dataset. They have been identified as potentially important variables to consider following discussion with our study collaborators and by heeding the encouragement from journals, such as the Lancet, to report data by sex and ethnicity to facilitate pooling of data for participant subgroups across studies, albeit, we note, that we are unlikely to be able to include a subgroup analysis based on ethnicity due to limited ethnic diversity in our trial sample. We hypothesise *"a priori"* that the treatment effect will be larger in participants with more knee symptoms compared to those with fewer knee symptoms. We do not have a strong hypothesis for the direction of any interaction effect for sex, but we hypothesise that the treatment effect would be lower in Black, Asian, or other minority groups compared to White ethnic group.

The primary analysis model, described in section 5.1, will be re-fitted to the data but will include the subgrouping variable (if not already in the model) and an interaction term between treatment and each of our subgrouping variables of interest to test whether the magnitude of the treatment effect depends on the subgrouping variable (separate models for each subgrouping variable).

All models will be fitted, however response prevalence to each categorical variable will be reported; if any one category contains only a small number of participants, merging of categories will be considered; this will be reported and highlighted as a limitation of the analysis. Model results will be presented as parameter estimates for the interaction terms, along with associated 95% confidence intervals (Table 12.3.10).

A graph showing the form of the interaction will be presented only if the overall significance of the interaction term is $p<0.1$ (the overall significance of the interaction terms will be calculated by comparing the log likelihood of a model with and without the interaction in it (or if the data are skewed, this comparison will be made using the Satorra-Bentler Scaled Chi-square Test (SBSCT)).

Despite all our hypotheses for our subgroup analyses, we remain clear that all subgroup analyses are exploratory given that our study sample size is not powered to detect them.

5.4.5 Longitudinal analysis of the primary outcome (KOOS-5) over time

A descriptive plot will be produced of the mean and 95% confidence interval of the KOOS-5 scores at each follow-up time-point, by treatment arm, and used to visualise how the mean KOOS-5 score changes over time (Figure 12.1.1). The models described in section 5.2.3 will also be used to test the null hypothesis that the magnitude of the treatment effect is the same at each follow-up time point by reporting the magnitude of the interaction term between treatment arm and time (as parameter estimates and 95% confidence intervals). The overall significance of the interaction term will also be reported using the method described in section 5.4.4.

6 Analysis methods (2): cost-effectiveness of AIE compared to AIE+B.

6.1 Health economic analysis overview

An economic evaluation will be conducted as part of the trial design. The aim of the economic evaluation is to address the question ***"what is the cost-effectiveness of Advice, written Information and Exercise instruction plus Bracing with adherence enhancing component (AIE+B) versus Advice, written Information and Exercise instruction (AIE) alone in the management of knee OA."***

The within-trial economic analysis will be performed using individual patient level data and will take the form of a cost-utility analysis, using quality-adjusted life years (QALYs) as the measure of outcomes. Costs and consequences of each group will be compared over the 12 months after randomisation with no extrapolation beyond the study period. Incremental cost-utility ratios will be calculated by taking a ratio of the difference in the mean costs and mean quality-adjusted life years (QALYs) between treatment arms.

The trial is conducted in the UK which has a National Health Service (NHS), providing publicly funded healthcare, primarily free of charge at the point of use. Therefore, the base-case economic analysis will be from the NHS perspective. Additional analysis will consider a healthcare and societal perspective to include private healthcare, out of pocket costs and productivity loses.

The analysis will be undertaken using Stata software. Results will be reported according with the Consolidated Health Economic Evaluation Reporting Standards (CHEERS) (Husereau et al 2022).

6.2 Health Outcomes

The outcome measure for the cost-utility analysis is Quality-adjusted life-years (QALYs) over 12 months. A QALY is a measure of the health of a person in which length of life is adjusted to reflect the quality of life. Length of life is measured in years, whilst quality of life is measured on a 'utility' scale anchored on 1 (perfect health) and zero (death). One year of life in perfect health is equal to 1 QALY (i.e. one multiplied by one). QALYs are preferred by health economists and decision makers as they are a generic measure of health gain allowing comparison across disease areas.

Health-related quality of life assessments will be completed by paticipants at baseline, 3, 6 and 12 months using the EQ-5D-5L, a generic measure of health related quality of life (Herdman et al 2011; Brooks, 1996) in order that QALYs over the 12-month time period can be calculated for each participant. In line with current NICE guidelines, responses for the EQ-5D-5L will be converted into index scores using the interim crosswalk value set for mapping from the EQ-5D-5L to the EQ-5D-3L following NICE's recommendations (NICE, 2019, Van Hout et al 2012). QALYs will be calculated for

individual patients and generated using the area under the baseline-adjusted utility curve, assuming linear interpolation between follow-up time points. To avoid bias, adjustment for differences between the two arms in baseline EQ-5D utility scores (as well as baseline covariates specified in the SAP) will be undertaken using regression-based adjustment (Manca et al 2005). EQ-5D values and QALYs over 12 months will be reported by treatment group and presented as means and standard deviations.

6.3 Resource use and cost collection

6.3.1 Costing of PROP-OA interventions (AIE+B and AIE)

Resources required to provide AIE+B include a knee brace per patient, x-rays required to ascertain brace type, the initial appointment (1 hour) and follow up appointment (30 minutes) with a physiotherapist, all written materials and SMS prompts for motivation. The cost of training a physiotherapist to deliver the intervention will also be calculated taking into account staff time for training, the expected number of patients seen in a year and any follow-up training required beyond the first year. Patient-level data on the brand and type of knee brace used will allow individual costing of braces. Unit costs of each brace will be sought from the NHS and/or the companies providing the braces. The base-case analysis will consider these individual-level costs and sensitivity analysis will use a range of brace costs to explore the impact on the economic evaluation results. In the base-case analysis resources required for AIE will be a 40 minute physiotherapy visit and written materials. Scenarios which explore a range of consultation durations and different staff grades will be undertaken in sensitivity anlayses. Unit costs of staff will be extracted from PSSRU 2022 (Jones et al, 2023) and costs of all written materials will be sought from trial staff.

6.3.2 Other resource utilisation

Resource use and costs will be based on the standard approach used in economic evaluations following the three-stage process: identification of resource use, measurement and valuation. Patient-level data will be collected on knee OA-related healthcare resource use at 6 and 12 months via participant questionnaires. Questions will request information regarding frequency of primary care visits (e.g. GP, nurse), visits to other health care professionals including primary or secondary physiotherapy, prescribed medications, tests and investigations, treatment (e.g. injections), secondary care consultations, inpatient stays and surgery. Information on both NHS and private health care visits will be collected. The recall period in each questionnaire will be the previous 6 months.

Health resource use information obtained from the self-reported questionnaires will be valued with unit cost data from standard sources, including the NHS reference costs (NHS England 2022) and Unit Costs of Health and Social Care (Jones, 2023). Unit costs of medications will be obtained from the British National Formulary (BNF, 2022). Unit cost for tests, investigations, inpatient hospital admissions and day care procedures will be estimated using from NHS reference costs using Healthcare Resource Group codes. Due to the lack of nationally representative unit cost estimates for private healthcare, this care will be costed as the NHS equivalent. The price year used in the analysis will be dependent on the most recent unit costs available.

Data on broader costs will also be collected, related to both out of pocket costs (e.g. over the counter medications) and time off work to calculate productivity losses. Information on occupation, further details of typical work activities and the nature of their employment (full time or part time) will be requested. The average wage for each respondent will be identified using UK Standard Occupational

Classification coding and annual earnings data for each job type (ONS, 2022). The analysis will use the human capital approach (Krol and Brouwer, 2014) and the self-reported days of absence will be multiplied by the respondent-specific wage rate. The human capital approach assumes that the value of lost work is equal to the amount of resources an individual would have been paid to do that work, and values productivity losses as a result of morbidity (or mortality) by measuring time lost from work and multiplying this with the gross wage of the person. Whilst there is no standard tool for capturing the costs of presenteeism, we will use the Single-Item Presenteeism Question (SIPQ) contained within the Work Productivity and Activity Impairment Questionnaire (WPAI) (Reilly, 1993). Our previous work has demonstrated this question to be both valid and responsive in patients with MSK pain (Kigozi *et al* 2014). This estimation of perceived percentage loss in productivity can be applied to person-specific wage rates using the human capital approach. Given the many uncertainties in the measurement of costs due to presenteeism, this will be presented as part of a secondary analysis.

6.4 Health economic analysis

Costs for the AIE+B and AIE arms will be presented for each broad cost category (NHS costs, private healthcare costs, patient-incurred costs, productivity costs) and disaggregated within each of these cost categories. Descriptive statistics (i.e. mean, SD, maximum and minimum values) will be reported for all relevant continuous variables. Binary and categorical variables will be presented in terms of percentages. The data for costs are likely to have a skewed distribution therefore a non-parametric comparison of means (e.g. bootstrapping) will be undertaken to estimate confidence intervals around costs. An incremental cost-utility analysis will be undertaken from a base-case NHS perspective to estimate the cost per quality adjusted life year (QALY) over 12 months follow-up, using patient level data on costs and trial outcomes. There will be no discounting of costs and QALYs as the time frame is not greater than one year.

The statistical analysis will be conducted on an intention to treat basis and will be based on imputed data. We will assess the extent of missing data in the patient-level costs and health outcomes collected during the 12-months follow-up and will apply a multiple imputation approach with predictive mean matching for imputing missing values of baseline utilities, EQ-5D-5L values and costs. We will assess the level and patterns of missingness in EQ-5D-5L dimensions. If there is a pattern of missing data in item non-response, we will impute missing dimensions (Simons (2015). The imputation model will include 25 imputed datasets and Rubin's rule will be used to combine the imputed datasets into one final imputed variable (Rubin and Schenker, 1991). The imputation model will include the cost and EQ-5D-5L outcomes of interest for the economic analysis and with adjustment for baseline covariates focussing on the same variables as outlined for the primary statistical analysis.

Uncertainty will be examined by estimating 95% confidence intervals (CIs) and cost-effectiveness acceptability curves (CEACs) (Fenwick and Byford, 2005). CEACs will be estimated using a net monetary benefit (NMB) approach with NMB be defined as:

λ x (Δ effect$_i$) - Δ cost$_i$,

where Δ effect$_i$ is the incremental person-level outcome associated with the AIE+B intervention, and (Δ cost$_i$), the additional costs due to the intervention, and λ=willingness to pay per unit of outcome gain. Using the output of the analysis, we will plot CEACs, showing the likelihood that the AIE+B intervention is cost effective given different assumptions about willingness to pay for QALYs. The NICE cost-effectiveness thresholds of £20,000 and £30,000 per additional QALY will be used to identify which treatment strategy represents best value for money (NICE, 2022).

Sensitivity analyses will be performed to:

1. estimate cost-utility analysis using a complete-case analysis.
2. estimate the cost-effectiveness from a health-care perspective, including private and patient related costs
3. consider a broader societal perspective, including patient specific productivity costs in addition to health-care costs
4. explore the impact on results of a range of costs for the knee brace, to account for regional differences in costs of braces to the NHS
5. exploring the impact on results of alternative scenarios for the duration of an AIE appointment, grade and type of health care professional undertaking AIE and AIE+B appointments
6. explore the impact of replacement braces and linked review appointments during the 12-month follow up period. Several scenarios will be explored including varying the percentage of patients needing a replacement, the number of extra review appointments required and if the brace is funded by the NHS or though patient out of pocket costs.
7. explore the impact of including costs of adverse events should they arise within the trial

Additional post-hoc sensitivity analyses will also be undertaken dependent on the emerging results, for example taking into account large resource use/cost outliers. This allows a full range of results to be presented for use by decision makers.

6.5 Model-based health economic analysis

Consent has been sought from participants to link their trial data to Hospital Episode Statistics, the National Joint Register and medical record review to measure receipt of knee arthroscopy and knee joint replacement. This data can then be used to extend the economic evaluation beyond the 12 month outcomes using decision modelling. A Markov model is proposed to extrapolate costs and QALYs over a lifetime time horizon However, long-term follow up (greater than 3 years) is needed and further funding will be required to undertake this work in the future. Therefore, full details of the proposed modelling methods are not yet developed and will not be presented in this analysis plan.

7 Analysis methods (3): patients' and physiotherapists' experience of AIE and AIE+B

7.1 Safety

7.1.1 Serious and unexpected adverse events

The process for reporting adverse events is described in the study protocol. The number and percentage of participants experiencing a serious and unexpected adverse event (SUAE) that was related to the treatment intervention will be reported, both overall and by treatment arm (Table 12.5.1). Percentages will be calculated from the number of participants randomised into the trial who receive at least one treatment visit with the treating physiotherapist i.e., defining a "safety population". Details of each event will be described in text or table as appropriate.

We do not anticipate that many participants will experience multiple SUAE, but if they do, then the number of SUAEs that each person experienced will be reported as a percentage of participants experiencing one or more SUAEs.

No formal statistical testing will be used to test whether the number of adverse events differs between treatment arms; hence the percentage of adverse events will be evaluated descriptively and assessed for clinical significance.

7.1.2 Expected adverse events.

Expected adverse events could occur from wearing a knee brace, such as skin redness and blistering, hence participants are asked to report such events to the physiotherapist at the follow-up treatment visit, and on the follow-up questionnaires. This data will be summarised using outline Table 12.5.2 and Table 12.5.3.

7.2 Acceptability and experiences of trial procedures and interventions to participants and physiotherapists

7.2.1 Treatment acceptability to participants

Acceptability of the trial treatments to participants will be assessed using frequency and percentages of the responses to the individual treatment acceptability questions based on the theoretical framework of acceptability (TFA) (Sekhon et al. 2022) collected on the 3-month follow-up questionnaire (Table 12.5.4). Multiple imputation will not be used to impute missing data.

7.2.2 Participant adherence to trial treatment (AIE or AIE+B)

Adherence to the intervention will be described using responses to the question: *"In the last 3- or 6-months (deleted as appropriate depending on time point), have you been following the advice and treatment you received from the physiotherapist as often as you were advised"*, stratified by treatment arm (Table 12.5.5). The data will be interpreted descriptively based on the magnitude of the differences observed, hence no statistical tests will be used to test for (a) differences in intervention adherence by treatment arm, or (b) whether there is a reduction in levels of intervention adherence over time.

For participants in the AIE+B arm only, descriptive statistics will be used to describe the frequency of brace use (past 7 days: number of days brace worn, number of hours per day brace worn, total time wearing the brace) as reported on the 3-, 6- and 12-month follow-up questionnaires (Table 12.5.6).

SMS text messaging data will also be used to produce two data plots:

1. The mean (and associated 95% confidence intervals) for the total time spent wearing the brace in the last 7 days for each occasion of SMS text data collection[14].
2. The proportion (and associated 95% confidence intervals) of those reporting they had worn the brace for the minimal time (as defined in section Table 4.1.1) for each occasion of SMS text data collection.

These plots will be used to observe visually whether the time spent wearing the brace is, on average, changing over time (e.g. is there evidence that the brace is being worn more regularly at later time points as participants follow the guidance from the physiotherapist to increase the amount of time they spend wearing the brace) and whether the proportion of people wearing the brace for a minimum

[14] Note that if the participant reporting wearing the brace for 0 or 1 days, they were not sent the text to ask for the number of hours they had worn the brace. Data for such participants has therefore been estimated using multiple imputation.

length of time increases over time. We will also report the average time spent wearing the brace over the 52 weeks of follow-up and the percentage of time-points where the brace was worn for the minimal time.

Analysis in this section will be based on analysis population 1 after multiple imputation has been applied.

7.2.3 Participant barriers to use of a brace

Barriers to brace use will be reported using numbers and percentages at each follow-up time point (Table 12.5.6). The denominator used in the percentage calculation will be the number of participants randomised to the AIE+B trial arm (with no imputation of missing data).

7.2.4 Intervention delivery

Delivery of the AIE and AIE+B interventions will be described using numbers and percentages from the case report form data completed by the physiotherapists delivering the interventions and will include details of advice and information giving, brace fit (where applicable) and application of motivational interviewing techniques (where applicable) (Table 12.5.7, Table 12.5.8 and Table 12.5.9).

7.2.5 Knee x-rays and brace allocation as delivered during the trial

This section of the analysis plan aims to inform how the trial results can be implemented in future clinical practice and what role plain x-ray may take in allocating brace types to participants in clinical practice. To explore this, clinician judgement on the most severely affected knee compartment to be treated, and brace type allocation, will be reported at two time points:

1. the clinical assessment (i.e. prior to x-ray data being available)
2. the initial treatment session (i.e. where x-ray data is available to assist the decision making process).

Clinician judgement for each of these variables will be described using numbers and percentages for all randomised participants with data available for the relevant variables on the case-report forms (Table 12.5.10). Crosstabulations will also be produced to explore if and how clinical judgment is changed when knee x-ray data are available to guide the decision-making process, with such an association summarised using percent agreement and an unweighted, unadjusted kappa statistic (with a 95% confidence interval) (Table 12.5.11 and Table 12.5.12)[15].

For the crosstabulation relating to brace-type allocation, two or more brace types could be potentially selected as suitable at the clinical eligibility assessment, making assessment of "agreement" more difficult. Two analyses will therefore be run: the first based only on patients who were allocated a single brace type at the clinical assessment, and the second where it is assumed that the judgement on brace type has not changed if it was considered as a potential brace at the clinical assessment.

We will also report the level of confidence that physiotherapists report when deciding on which brace to select for the participant, and on reading the x-ray as part of brace selection (Table 12.5.13) and a

[15] Guidelines by (Landis & Koch. 1977) will be used to judge the magnitude of the Kappa statistics, but will be interpreted with caution as the Kappa statistics could be artificially inflated due to lack of independence between the two ratings i.e. the second judgement was made with knowledge of the first judgement, therefore the two ratings are not independent (Sim & Wright. 2005).

table will be produced to document the number of participants that had their brace type changed post-randomisation, to include the brace they were initially allocated to, the brace that they changed to, and the reason for the change in brace allocation from the allocation originally given (Table 12.5.14).

8 Further research questions of interest

The aim of this analysis plan is to explore the clinical and cost-effectiveness of AIE compared to AIE+B using a (largely) treatment policy approach. It's acknowledged however, further research questions could be asked of the trial data that are not included in this analysis plan.

Analysis plans for these questions could potentially be written after the results from the clinical and cost-effectiveness analyses have been published, so are beyond the scope of the current analysis plan as reported. Examples of such research questions include: further exploration of data on patient's experience of using the brace: statistical models could be fitted to the data to explore whether brace adherence differs by brace type/OA location; to explore factors that predict brace adherence; and whether objective measures of brace use (captured by ibuttons fitted to the Ossur Unloader One (tibiofemoral brace) and Ossur Formfit Knee Hinged (neutral stabilising brace) brace) offer insight into self-reported measures of brace adherence. Further modelling of predictors of outcome trajectories over time and mediation analysis, to understand the mechanisms of action if a significant treatment effect is found in the trial, could also be considered. We did not include a mediation analysis in the main trial analysis plan as we have limited data that could be used to define mediators of the treatment effect. However, it is acknowledged that treatment self-efficacy could be a potential candidate mediator to explore.

9 Software

Analysis in this analysis plan will be generated using STATA software and will use the most up-to-date version of the software available for analysis. The software version number will be reported in any published papers arising from the trial.

10 Data management plan

Trial data collection followed Data Management Plan (DMP) version 0.6 – 21st Jan 2021.

11 References

Austin PC, White IR, Lee DS, van Buuren S. Missing data in clinical research: A tutorial on multiple imputation. Can J Cardiol. 2021;37(9):1322-1331. doi: S0828-282X(20)31111-9 [pii].

BNF, 2022. https://bnf.nice.org.uk/ (Accessed 5 April, 2023)

Brady TJ. Measures of self-efficacy: Arthritis self-efficacy scale (ASES), arthritis self-efficacy scale-8 item (ASES-8), children's arthritis self-efficacy scale (CASE), chronic disease self-efficacy scale (CDSES), parent's arthritis self-efficacy scale (PASE), and rheumatoid arthritis self-efficacy scale (RASE). Arthritis Care Res (Hoboken). 2011;63 Suppl 11:473. doi: 10.1002/acr.20567 [doi].

Brooks R. EuroQol: the current state of play. Health Policy 1996; 37:53–72.

Clark TP, Kahan BC, Phillips A, White I, Carpenter JR. Estimands: Bringing clarity and focus to research questions in clinical trials. BMJ Open. 2022;12(1):e052953. http://bmjopen.bmj.com/content/12/1/e052953.abstract. doi: 10.1136/bmjopen-2021-052953.

Cro S, Morris TP, Kenward MG, Carpenter JR. Sensitivity analysis for clinical trials with missing continuous outcome data using controlled multiple imputation: A practical guide. Statistics in Medicine. 2020;39(21):2815-2842. https://doi.org/10.1002/sim.8569. doi: 10.1002/sim.8569.

Cro S, Morris TP, Kahan BC, Cornelius VR, Carpenter JR. A four-step strategy for handling missing outcome data in randomised trials affected by a pandemic. BMC Medical Research Methodology. 2020;20(1):208. https://doi.org/10.1186/s12874-020-01089-6. doi: 10.1186/s12874-020-01089-6.

Cudejko T, van der Esch M, van der Leeden M, et al. Effect of Soft Braces on Pain and Physical Function in Patients With Knee Osteoarthritis: Systematic Review With Meta-Analyses. Arch Phys Med Rehabil. 2018;99:153-163.

Duong V, Hunter DJ, Nicolson PJA. Predictors and measures of adherence to core treatments for osteoarthritis. *Clin Geriatr Med*. 2022;38(2):345-360. doi: 10.1016/j.cger.2021.11.007.

Fenwick E, O'Brien BJ, Briggs A. Cost-Effectiveness acceptability curves– facts, fallacies and frequently asked questions. Health Econ 2004; 13:405–15.

Foster NE, Thomas E, Barlas P, Hill JC, Young J, Mason E, Hay EM. Acupuncture as an adjunct to exercise based physiotherapy for osteoarthritis of the knee: randomised controlled trial. BMJ 2007;335:436.

Foster NE, Nicholls E, Holden MA, Healey EL, Hay EM, BEEP trial team. Improving the effectiveness of exercise therapy for adults with knee osteoarthritis: A pragmatic randomized controlled trial (BEEP trial). *Arch Rehabil Res Clin Transl*. 2023;5(2):100266. doi: 10.1016/j.arrct.2023.100266.

Hay EM, Foster NE, Thomas E, Peat G, Phelan M, Yates HE, Blenkinsopp A, Sim J. Pragmatic randomized clinical trial of the effectiveness of community physiotherapy and enhanced pharmacy review for knee pain in older people presenting to primary care. BMJ 2006;333:995-998.

Herdman M, Gudex C, Lloyd A, et al. Development and preliminary testing of the new five-level version of EQ-5D (EQ-5D-5L). Qual Life Res 2011; 20: 1727–36.

Holden MA, Callaghan M, Felson D, et al. Clinical and cost-effectiveness of bracing in symptomatic knee osteoarthritis management: Protocol for a multicentre, primary care, randomised, parallel-group, superiority trial. BMJ Open. 2021;11(3):e048196-048196. doi: 10.1136/bmjopen-2020-048196.

Huque MH, Carlin JB, Simpson JA, Lee KJ. A comparison of multiple imputation methods for missing data in longitudinal studies. BMC Medical Research Methodology. 2018;18(1):168. https://doi.org/10.1186/s12874-018-0615-6. doi: 10.1186/s12874-018-0615-6.

Hurtig-Wennlöf A, Hagströmer M, Olsson LA. The international physical activity questionnaire modified for the elderly: Aspects of validity and feasibility. Public Health Nutr. 2010;13(11):1847-1854. doi: 10.1017/S1368980010000157 [doi].

Husereau D, Drummond M, Augustovski F, de Bekker-Grob E, Briggs AH, Carswell C, Caulley L, Chaiyakunapruk N, Greenberg D, Loder E, Mauskopf J, Mullins CD, Petrou S, Pwu RF, Staniszewska S; CHEERS 2022 ISPOR Good Research Practices Task Force. Consolidated Health Economic Evaluation Reporting Standards 2022 (CHEERS 2022) Statement: Updated Reporting Guidance for Health Economic Evaluations. Value Health. 2022 Jan;25(1):3-9. doi: 10.1016/j.jval.2021.11.1351.

ICH E9 (R1) addendum on estimands and sensitivity analysis in clinical trials to the guideline on statistical principles for clinical trials European Medicines Agency. Published 30th July, 2020. https://www.ema.europa.eu/en/documents/scientific-guideline/ich-e9-r1-addendum-estimands-sensitivity-analysis-clinical-trials-guideline-statistical-principles_en.pdf (accessed 1st December 2022).

Jones KC, Weatherly H, Birch S, Castelli A, Chalkley M, Dargan A, Forder JE, Gao J, Hinde S, Markham S et al. Unit Costs of Health and Social Care 2022 Manual. Technical report. 2023 Personal Social Services Research Unit (University of Kent) & Centre for Health Economics (University of York), Kent, UK 10.22024/UniKent/01.02.100519.

Kahan B, Morris T. Assessing potential sources of clustering in individually randomised trials. BMC Medical Research Methodology 2013;13:58.

Kigozi et al. Construct validity and responsiveness of the single-item presenteeism question in patients with lower back pain for the measurement of presenteeism. Spine 2014;39(5):409-416.

Kingsbury SR, Corp N, Watt FE, et al. Harmonising data collection from osteoarthritis studies to enable stratification: Recommendations on core data collection from an arthritis research UK clinical studies group. *Rheumatology (Oxford)*. 2016;55(8):1394-1402. doi: 10.1093/rheumatology/kew201.

Kleinke K. Multiple imputation by predictive mean matching when sample size is small. Methodology. 2018;14(1):3-15. https://doi.org/10.1027/1614-2241/a000141. doi: 10.1027/1614-2241/a000141.

Krol M, Brouwer W. How to Estimate Productivity Costs in Economic Evaluations. Pharmacoeconomics 2014; 4:335–44.

Landis JR, Koch GG. The measurement of observer agreement for categorical data. Biometrics. 1977;33(1):159-174. http://www.jstor.org/stable/2529310. doi: 10.2307/2529310.

MacFarlane GJ, Croft PR, Schollum J, Silman AJ. Widespread pain: Is an improved classification possible? J Rheumatol. 1996;23(9):1628-1632.

Madley-Dowd P, Hughes R, Tilling K, Heron J. The proportion of missing data should not be used to guide decisions on multiple imputation. J Clin Epidemiol. 2019;110:63-73. https://www.sciencedirect.com/science/article/pii/S0895435618308710. doi: 10.1016/j.jclinepi.2019.02.016.

Manca A, Hawkins N, Sculpher M. Estimating mean QALYs in trial-based cost-effectiveness analysis: The importance of controlling for baseline utility Article in Health Economics. Health Econ 2005; 14:487–96.

Morris TP, White IR, Royston P. Tuning multiple imputation by predictive mean matching and local residual draws. BMC Medical Research Methodology. 2014;14(1):75. https://doi.org/10.1186/1471-2288-14-75. doi: 10.1186/1471-2288-14-75.

Morris TP, Walker AS, Williamson EJ, White IR. Planning a method for covariate adjustment in individually randomised trials: A practical guide. Trials. 2022;23(1):328. https://doi.org/10.1186/s13063-022-06097-z. doi: 10.1186/s13063-022-06097-z.

Moyer RF, Birmingham TB, Bryant DM, Giffin JR, Marriott KA, Leitch KM. Valgus bracing for knee osteoarthritis: a metaanalysis of randomized trials. Arthritis Care Res 2015;67:493-501.

National Institute for Health and Care Excellence (NICE) (2017) NICE health technology evaluations: the manual. PMG36. 2022. https://www.nice.org.uk/process/pmg36 (Accessed 5 April 2023)

National Institute for Health and Care Excellence (NICE) Position statement on use of the EQ-5D-5L value set for England. 2019 Available: https://www.nice.org.uk/about/what-we-do/our-programmes/nice-guidance/technology-appraisal-guidance/eq-5d-5l [Accessed 5 April 2023].

Nguyen CD, Carlin JB, Lee KJ. Practical strategies for handling breakdown of multiple imputation procedures. Emerging Themes in Epidemiology. 2021;18(1):5. https://doi.org/10.1186/s12982-021-00095-3. doi: 10.1186/s12982-021-00095-3.

NHS England. 2020/21 National Cost Collection Data Publication. 2022. https://www.england.nhs.uk/publication/2020-21-national-cost-collection-data-publication/ (Accessed 5 April 2023)

Office for National Statistics. Annual survey of hours and earnings 2022. https://www.ons.gov.uk/employmentandlabourmarket/peopleinwork/earningsandworkinghours/bulletins/annualsurveyofhoursandearnings/2022 (Accessed 5 April 2023).

Orkin AM, Gill PJ, Ghersi D, et al. Guidelines for reporting trial protocols and completed trials modified due to the COVID-19 pandemic and other extenuating circumstances: The CONSERVE 2021 statement. *JAMA*. 2021;326(3):257-265. doi: 10.1001/jama.2021.9941.

Parpia S, Morris TP, Phillips MR, et al. Sensitivity analysis in clinical trials: Three criteria for a valid sensitivity analysis. Eye. 2022;36(11):2073-2074. https://doi.org/10.1038/s41433-022-02108-0. doi: 10.1038/s41433-022-02108-0.

Pham T, van der Heijde D, Altman RD, et al. OMERACT-OARSI initiative: Osteoarthritis research society international set of responder criteria for osteoarthritis clinical trials revisited. Osteoarthritis and Cartilage. 2004;12(5):389-399.

Reilly MC, Zbrozek AS, Dukes E: The validity and reproducibility of a work productivity and activity impairment measure. PharmacoEconomics 1993; 4(5):353-365

Rombach I, Gray AM, Jenkinson C, Murray DW, Rivero-Arias O. Multiple imputation for patient reported outcome measures in randomised controlled trials: Advantages and disadvantages of imputing at the item, subscale or composite score level. BMC Med Res Methodol. 2018;18(1):87-6. doi: 10.1186/s12874-018-0542-6 [doi].

Roos EM, Lohmander LS. The Knee injury and Osteoarthritis Outcome Score (KOOS): from joint injury to osteoarthritis. Health and Quality of Life Outcomes 2003;1:64.

Rubin D, Schenker N. Multiple imputation in health-are databases: An overview and some applications. Stat Med 1991; 10:585–98.

Schnitzer, T.J., Conaghan, P.G., Berenbaum, F. et al. Effect size varies based on calculation method and may affect interpretation of treatment effect: an illustration using randomised clinical trials in osteoarthritis. Adv Rheumatol 64, 31 (2024). https://doi.org/10.1186/s42358-024-00358-y

Schulz KF, Altman DG, Moher D. CONSORT 2010 statement: Updated guidelines for reporting parallel group randomised trials. BMJ. 2010;340:c332. http://www.bmj.com/content/340/bmj.c332.abstract. doi: 10.1136/bmj.c332.

Sekhon M, Cartwright M, Francis JJ. Development of a theory-informed questionnaire to assess the acceptability of healthcare interventions. BMC Health Serv Res. 2022 Mar 1;22(1):279. doi: 10.1186/s12913-022-07577-3. PMID: 35232455; PMCID: PMC8887649.

Sim J, Wright CC. The kappa statistic in reliability studies: Use, interpretation, and sample size requirements. Phys Ther. 2005;85(3):257-268. https://doi.org/10.1093/ptj/85.3.257. Accessed 1/19/2023. doi: 10.1093/ptj/85.3.257.

Simons CL, Rivero-Arias O, Yu LM, Simon J. Multiple imputation to deal with missing EQ-5D-3L data: Should we impute individual domains or the actual index? Qual Life Res. 2015 Apr;24(4):805-15.

Singer, J., & Willet, J. (2003). Applied longitudinal data analysis (First ed.). USA: Oxford University Press.

Snaith RP. The hospital anxiety and depression scale. Health Qual Life Outcomes. 2003;1:29-29. doi: 1477-7525-1-29 [pii].

Squyer E, Stamper DL, Hamilton DT, Sabin JA, Leopold SS. Unloader knee braces for osteoarthritis: Do patients actually wear them? Clin Orthop Relat Res. 2013;471(6):1982-1991. doi: 10.1007/s11999-013-2814-0.

StataCorp. 2022. Stata Statistical Software: Release 17. College Station, TX: StataCorp LLC.

Troncoso P, Ana Morales-Gómez. Estimating the complier average causal effect via a latent class approach using gsem. The Stata Journal. 2022;22(2):404-415. https://doi.org/10.1177/1536867X221106416. doi: 10.1177/1536867X221106416.

Twisk J; Bosman L; Hoekstra T; Rijnhart J; Welten M and Heymans M. Different ways to estimate treatment effects in randomised controlled trials. Contemporary Clinical Trials Communications. 2018;10:80-85. https://www.sciencedirect.com/science/article/pii/S2451865417301849. doi: 10.1016/j.conctc.2018.03.008.

van Buuren S. Multiple imputation of discrete and continuous data by fully conditional specification. Stat Methods Med Res. 2007;16(3):219-242. https://doi.org/10.1177/0962280206074463. doi: 10.1177/0962280206074463.

van Hout B, Janssen MF, Feng YS, et al. Interim scoring for the EQ-5D-5L: Mapping the EQ-5D-5L to EQ-5D-3L value sets. Value Health. 2012;15(5):708-715. doi: 10.1016/j.jval.2012.02.008 [doi].

Van Lancker K, Tarima S, Bartlett J, et al. Estimands and their estimators for clinical trials impacted by the COVID-19 pandemic: A report from the NISS ingram olkin forum series on unplanned clinical trial disruptions. Statistics in Biopharmaceutical Research. 2023;15(1):94-111. https://doi.org/10.1080/19466315.2022.2094459. doi: 10.1080/19466315.2022.2094459.

Weir CB, Jan A. BMI classification percentile and cut off points. In: StatPearls. Treasure Island (FL): StatPearls Publishing LLC; 2022. NBK541070 [bookaccession].

Wen L, Terrera GM, Seaman SR. Methods for handling longitudinal outcome processes truncated by dropout and death. Biostatistics. 2017;19(4):407-425. https://doi.org/10.1093/biostatistics/kxx045. Accessed 4/22/2023. doi: 10.1093/biostatistics/kxx045.

White IR, Royston P, Wood AM. Multiple imputation using chained equations: Issues and guidance for practice. Stat Med. 2011;30(4):377-399. doi: 10.1002/sim.4067 [doi].

12 Outline tables and figures

12.1 Clinical effectiveness paper – primary figures

Figure 12.1.1: CONSORT flow diagram

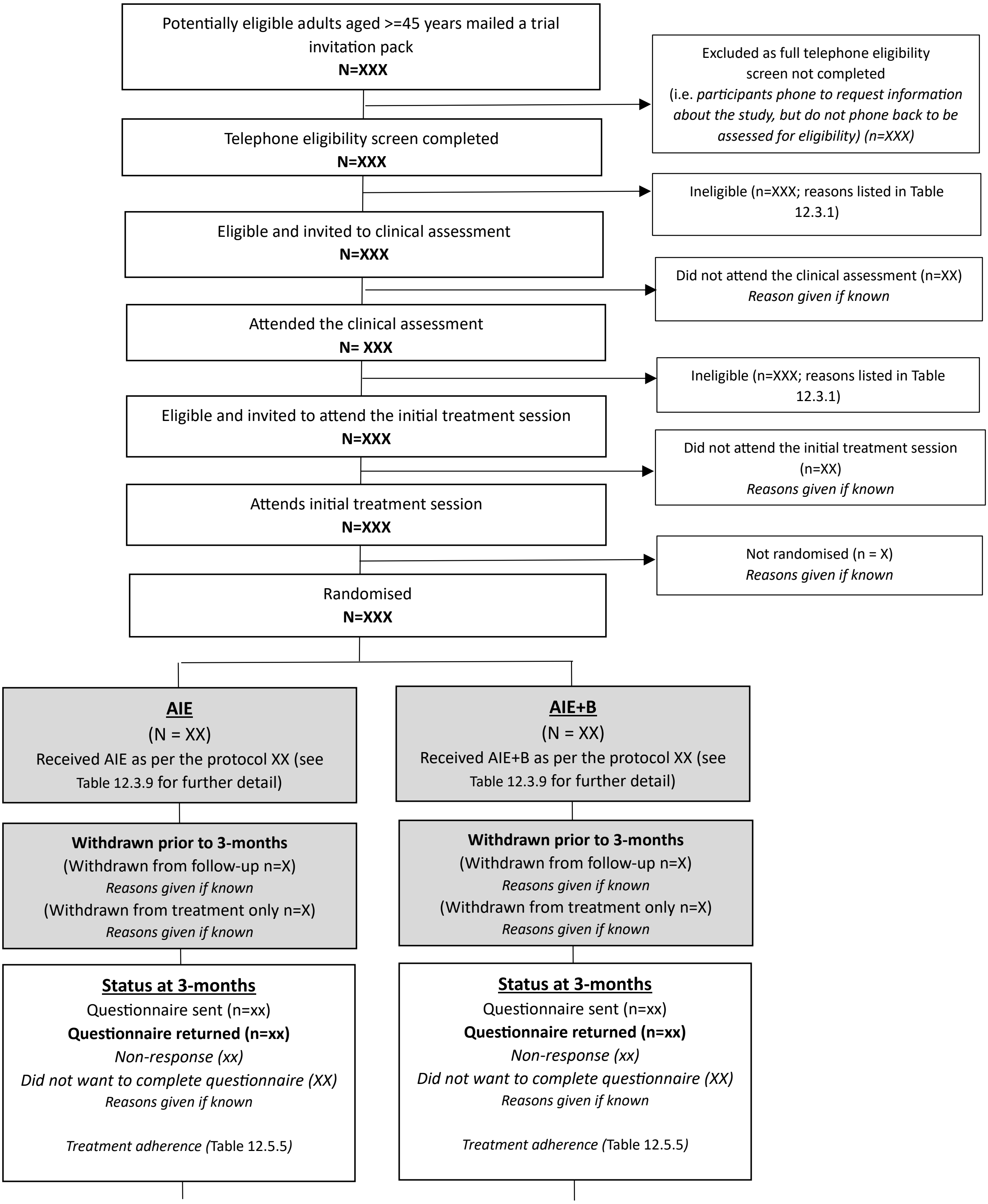

Withdrawn between 3- and 6-months
(Withdrawn from follow-up n=X)
Reasons given if known
(Withdrawn from treatment only n=X)
Reasons given if known

<u>Status at 6-months</u>
Questionnaire sent (n=xx)
Questionnaire returned (n=xx)
Non-response (xx)
Did not want to complete questionnaire (XX)
Reasons given if known

*Treatment adherence (*Table 12.5.5*)*

Withdrawn between 6- and 12-months
(Withdrawn from follow-up n=X)
Reasons given if known
(Withdrawn from treatment only n=X)
Reasons given if known

<u>Status at 12-months</u>
Questionnaire sent (n=xx)
Questionnaire returned (n=xx)
Non-response (xx)
Did not want to complete questionnaire (XX)
Reasons given if known

*Treatment adherence (*Table 12.5.5*)*

Withdrawn between 3- and 6-months
(Withdrawn from follow-up n=X)
Reasons given if known
(Withdrawn from treatment only n=X)
Reasons given if known

<u>Status at 6-months</u>
Questionnaire sent (n=xx)
Questionnaire returned (n=xx)
Non-response (xx)
Did not want to complete questionnaire (XX)
Reasons given if known

*Treatment adherence (*Table 12.5.5*)*

Withdrawn between 6- and 12-months
(Withdrawn from follow-up n=X)
Reasons given if known
(Withdrawn from treatment only n=X)
Reasons given if known

<u>Status at 12-months</u>
Questionnaire sent (n=xx)
Questionnaire returned (n=xx)
Non-response (xx)
Did not want to complete questionnaire (XX)
Reasons given if known

*Treatment adherence (*Table 12.5.5*)*

Figure 12.1.1: Descriptive plot of the mean KOOS-5 scores and 95% confidence intervals over time

<<Insert here a descriptive plot of the KOOS-5 outcome over time and annotate with results from the treatment by time interaction test>>

12.2 Clinical effectiveness paper – primary tables

Table 12.2.1: Key baseline characteristics

	All randomised participants N=XXX$^{\alpha}$	AIE N=XX$^{\alpha}$	AIE+B N=XX$^{\alpha}$
**Demographics**			
Age (years): Mean (SD)	xx (xx)	xx (xx)	xx (xx)
Female sex	xx (xx)	xx (xx)	xx (xx)
Ethnic group			
White	xx (xx)	xx (xx)	xx (xx)
Black-Caribbean	xx (xx)	xx (xx)	xx (xx)
Black-African	xx (xx)	xx (xx)	xx (xx)
Black – Other	xx (xx)	xx (xx)	xx (xx)
Indian	xx (xx)	xx (xx)	xx (xx)
Pakistani	xx (xx)	xx (xx)	xx (xx)
Bangladeshi	xx (xx)	xx (xx)	xx (xx)
Chinese	xx (xx)	xx (xx)	xx (xx)
Prefer not to say	xx (xx)	xx (xx)	xx (xx)
Other	xx (xx)	xx (xx)	xx (xx)
Left school to attend full-time education or university	xx (xx)	xx (xx)	xx (xx)
Currently in paid employment (full or part-time)			
Index of multiple deprivation (IMD) (1 - 32,844): Mean (SD)	xx (xx)	xx (xx)	xx (xx)
IMD Quintile			
1: IMD 1 to 6568	xx (xx)	xx (xx)	xx (xx)
2: IMD 6569 to 13137	xx (xx)	xx (xx)	xx (xx)
3: IMD 13138 to 19706	xx (xx)	xx (xx)	xx (xx)
4: IMD 19707 to 26275	xx (xx)	xx (xx)	xx (xx)
5: IMD 26276 to 32844	xx (xx)	xx (xx)	xx (xx)
**General Health and Wellbeing**			
Pain in the last 4 weeks lasting for a day or longer in any part of the body	xx (xx)	xx (xx)	xx (xx)
Has a long-term (>12 months) physical or mental health condition, disability or illness	xx (xx)	xx (xx)	xx (xx)
Blindness or partial sight	xx (xx)	xx (xx)	xx (xx)
A breathing condition e.g. asthma or COPD	xx (xx)	xx (xx)	xx (xx)
Cancer (diagnosis or treatment in the last 5 years)	xx (xx)	xx (xx)	xx (xx)
Deafness or hearing loss	xx (xx)	xx (xx)	xx (xx)
Diabetes	xx (xx)	xx (xx)	xx (xx)
Heart condition e.g. angina or atrial fibrillation	xx (xx)	xx (xx)	xx (xx)
High blood pressure	xx (xx)	xx (xx)	xx (xx)
Kidney or liver disease	xx (xx)	xx (xx)	xx (xx)
A mental health condition	xx (xx)	xx (xx)	xx (xx)
A neurological condition e.g. epilepsy	xx (xx)	xx (xx)	xx (xx)
A stroke (which affects day-to-day life)	xx (xx)	xx (xx)	xx (xx)
Takes more than 5 medications on a regular basis	xx (xx)	xx (xx)	xx (xx)
Body-mass index (BMI) (kg/m^2): Mean (SD)	xx (xx)	xx (xx)	xx (xx)

Categorised BMI			
Underweight: BMI <18.5 kg/m^2	xx (xx)	xx (xx)	xx (xx)
Normal weight: BMI>=18.5kg/m^2&<24.9 kg/m^2	xx (xx)	xx (xx)	xx (xx)
Overweight: BMI >= 24.9 kg/m^2 & < 29.9 kg/m^2	xx (xx)	xx (xx)	xx (xx)
Obese: BMI >= 29.9 kg/m^2	xx (xx)	xx (xx)	xx (xx)
HADS: anxiety (0-21): Mean (SD)	xx (xx)	xx (xx)	xx (xx)
HADS: depression (0-21): Mean (SD)	xx (xx)	xx (xx)	xx (xx)
**Stratifying variables in the randomisation process**			
Clinic site			
Staffordshire	xx (xx)	xx (xx)	xx (xx)
Manchester	xx (xx)	xx (xx)	xx (xx)
Cheshire	xx (xx)	xx (xx)	xx (xx)
Northumbria	xx (xx)	xx (xx)	xx (xx)
Predominant compartmental distribution of knee OA based on combination of clinical assessment and x-rays			
Medial tibiofemoral	xx (xx)	xx (xx)	xx (xx)
Lateral tibiofemoral	xx (xx)	xx (xx)	xx (xx)
Patellofemoral	xx (xx)	xx (xx)	xx (xx)
No clear predominant compartmental involvement	xx (xx)	xx (xx)	xx (xx)
Instability (buckling): Knee buckled at least once in the last 3-months			
Yes	xx (xx)	xx (xx)	xx (xx)
No/Not sure	xx (xx)	xx (xx)	xx (xx)
**Trial outcome measures (where measured at baseline)**			
KOOS-5 (primary outcome) (0-100): Mean (SD)	xx (xx)	xx (xx)	xx (xx)
KOOS: pain (0-100): Mean (SD)	xx (xx)	xx (xx)	xx (xx)
KOOS: symptoms (0-100): Mean (SD)	xx (xx)	xx (xx)	xx (xx)
KOOS: Activities of daily living (0-100): Mean (SD)	xx (xx)	xx (xx)	xx (xx)
KOOS: Sport/recreation (0-100): Mean (SD)	xx (xx)	xx (xx)	xx (xx)
KOOS: Knee related quality of life (0-100): Mean (SD)	xx (xx)	xx (xx)	xx (xx)
KOOS-4	xx (xx)	xx (xx)	xx (xx)
WOMAC			
Pain (0-20): Mean (SD)	xx (xx)	xx (xx)	xx (xx)
Stiffness (0-8): Mean (SD)	xx (xx)	xx (xx)	xx (xx)
Function (0-68): Mean (SD)	xx (xx)	xx (xx)	xx (xx)
Last 7 days, knee pain during activity in the knee (0-10): Mean (SD)	xx (xx)	xx (xx)	xx (xx)
Intermittent and constant pain (ICOAP)			
Constant pain subscale (0-100): Mean (SD)	xx (xx)	xx (xx)	xx (xx)
Intermittent pain subscale (0-100): Mean (SD)	xx (xx)	xx (xx)	xx (xx)
Total pain subscale (0-100): Mean (SD)	xx (xx)	xx (xx)	xx (xx)
Arthritis self-efficacy (SEE) (1-10): Mean (SD)	xx (xx)	xx (xx)	xx (xx)
Physical activity (IPAQ-E) (MET minutes per week; 0–19278): Median (IQR)	Xxxx (xxxx, xxxx)	Xxxx (xxxx, xxxx)	Xxxx (xxxx, xxxx)
Social participation (PROMIS) (27.5-64.2): Mean (SD)	xx (xx)	xx (xx)	xx (xx)
**X-ray characteristics**			
Kellgren-Lawrence (KL) highest grade per knee			
0	xx (xx)	xx (xx)	xx (xx)
1	xx (xx)	xx (xx)	xx (xx)

2	xx (xx)	xx (xx)	xx (xx)
3	xx (xx)	xx (xx)	xx (xx)
4	xx (xx)	xx (xx)	xx (xx)

Figures are numbers (percentages in brackets) unless otherwise stated. IQR = interquartile range, SD = Standard deviation. All outcome measures completed in reference to the knee to be treated. α = baseline questionnaire data is missing for xxx participants hence analysis based on xxx participants with data. IPAQ-E International Physical Activity Questionnaire - Elderly; ICOAP Intermittent & Constant Osteoarthritis Pain; KL = Kellgren-Lawrence; KOOS Knee Osteoarthritis Outcomes Score; NRS Numerical Rating Scale; WOMAC Western Ontario and McMaster Universities Arthritis Index α add in a footnote to explain the reason why the number of people with x-ray data does not equal the number of people randomised (if this happens) and the intra/inter rater reliability of the x-ray scoring. Variables in the randomisation stratification and trial outcome measures sections of this table, as well as age, sex, ethnicity, HADS scores (anxiety and depression) are based on analysis population 1; the remaining variables are reported using non-imputed data.

Table 12.2.2: Treatment effect estimates for the primary outcome (KOOS-5) at the 6-month follow-up

KOOS-5	6-months
**Descriptive statistics**	
AIE: Mean (SD)	xx(xx)
AIE+B: Mean (SD)	xx(xx)
**Primary analysis: Treatment effect: AIE vs AIE+B: Adjusted$^{\alpha}$ mean difference (95% CI)**	xx (xx, xx)
**Sensitivity analyses: Treatment effect: AIE vs AIE+B: Adjusted$^{\alpha}$ mean difference (95% CI)**	
Excluding brace adherence data from the imputation model as only available for the AIE+B arm of the trial	xx (xx, xx)
Additionally accounting for variation in outcomes between physiotherapists	xx (xx, xx)
Exploring the impact of the data not being missing at random (MAR)	
Delta = X	xx (xx, xx)
Delta = X	xx (xx, xx)
Delta = X	xx (xx, xx)
Delta = X	xx (xx, xx)
Delta = X for participants with all KOOS-5 subscales missing; 0 otherwise	xx (xx, xx)
Delta = X for participants with all KOOS-5 subscales missing; 0 otherwise	xx (xx, xx)
Delta = X for participants with all KOOS-5 subscales missing; 0 otherwise	xx (xx, xx)
Delta = X for participants with all KOOS-5 subscales missing; 0 otherwise	xx (xx, xx)
Estimating the treatment effect from a longitudinal model with no imputation of missing data	xx (xx, xx)
**Supplementary analyses: Treatment effect: AIE vs AIE+B: Adjusted$^{\alpha}$ mean difference (95% CI)**	
For the hypothetical scenario that all participants had clinical treatment delivered, and data collected, according to the study protocol	xx (xx, xx)
For the hypothetical scenario that all participants were recruited in a world where COVID-19 already exists, and where individuals could suffer from COVID-19 infections (Van Lancker K et al. 2023)	xx (xx, xx)
**Supplementary analyses: complier average causal effect (CACE) analysis: mean difference (95% CI)**	
For the principal stratum of participants who adhered to the bracing component of the AIE+B trial arm (adherence definition 1$^{\beta}$)	xx (xx, xx)
For the principal stratum of participants who adhered to the bracing component of the AIE+B trial arm (adherence definition 2$^{\mu}$)	xx (xx, xx)

$^{\alpha}$ Adjusted for PROP-OA clinic site, predominant compartmental distribution based on clinical and x-ray findings, presence/absence of instability (buckling), age, sex, baseline anxiety, baseline depression, baseline KOOS-5 score $^{\beta}$ Participants defined as being adherent to treatment if they report they wore the brace for minimal time at 3-months **or** 6-months (as defined using data on the self-reported questionnaires at these time points $^{\mu}$ Participants defined as being adherent to treatment if they report they wore the brace for minimal time at 3-months **and** 6-months (as defined using data on the self-reported questionnaires at these time points) . CI = confidence interval

Table 12.2.3: Treatment effect estimates for the primary outcome (KOOS-5) at the 3-month and 12-month follow-up and the secondary outcome measures at all time-points

Outcome measure	3-months	6-months	12-months
KOOS-5: (0-100)			
AIE: Mean (SD)	xx (xx)	See Table 12.2.2	xx (xx)
AIE+B: Mean (SD)	xx (xx)	See Table 12.2.2	xx (xx)
AIE vs AIE+B: Adjusted$^{\alpha}$ mean difference (95% CI)	xx (xx, xx)	See Table 12.2.2	xx (xx, xx)
AIE vs AIE+B: Adjusted$^{\alpha}$ mean difference (95% CI) estimated from a longitudinal mixed model on non-imputed data	xx (xx, xx)	See Table 12.2.2	xx (xx, xx)
KOOS: pain (0-100)			
AIE: Mean (SD)	xx (xx)	xx (xx)	xx (xx)
AIE+B: Mean (SD)	xx (xx)	xx (xx)	xx (xx)
AIE vs AIE+B: Adjusted$^{\alpha}$ mean difference (95% CI)	xx (xx, xx)	xx (xx, xx)	xx (xx, xx)
KOOS: symptoms (0-100)			
AIE: Mean (SD)	xx (xx)	xx (xx)	xx (xx)
AIE+B: Mean (SD)	xx (xx)	xx (xx)	xx (xx)
AIE vs AIE+B: Adjusted$^{\alpha}$ mean difference (95% CI)	xx (xx, xx)	xx (xx, xx)	xx (xx, xx)
KOOS: activities of daily living (0-100)			
AIE: Mean (SD)	xx (xx)	xx (xx)	xx (xx)
AIE+B: Mean (SD)	xx (xx)	xx (xx)	xx (xx)
AIE vs AIE+B: Adjusted$^{\alpha}$ mean difference (95% CI)	xx (xx, xx)	xx (xx, xx)	xx (xx, xx)
KOOS: sport/recreation (0-100)			
AIE: Mean (SD)	xx (xx)	xx (xx)	xx (xx)
AIE+B: Mean (SD)	xx (xx)	xx (xx)	xx (xx)
AIE vs AIE+B: Adjusted$^{\alpha}$ mean difference (95% CI)	xx (xx, xx)	xx (xx, xx)	xx (xx, xx)
KOOS: Knee related quality of life (0-100)			
AIE: Mean (SD)	xx (xx)	xx (xx)	xx (xx)
AIE+B: Mean (SD)	xx (xx)	xx (xx)	xx (xx)
AIE vs AIE+B: Adjusted$^{\alpha}$ mean difference (95% CI)	xx (xx, xx)	xx (xx, xx)	xx (xx, xx)
KOOS-4: (0-100)			

AIE: Mean (SD)	xx (xx)	xx (xx)	xx (xx)
AIE+B: Mean (SD)	xx (xx)	xx (xx)	xx (xx)
AIE vs AIE+B: Adjusted$^{\alpha}$ mean difference (95% CI)	xx (xx, xx)	xx (xx, xx)	xx (xx, xx)
WOMAC pain: (0-20)			
AIE: Mean (SD)	xx (xx)	xx (xx)	xx (xx)
AIE+B: Mean (SD)	xx (xx)	xx (xx)	xx (xx)
AIE vs AIE+B: Adjusted$^{\alpha}$ mean difference (95% CI)	xx (xx, xx)	xx (xx, xx)	xx (xx, xx)
WOMAC stiffness: (0-8)			
AIE: Mean (SD)	xx (xx)	xx (xx)	xx (xx)
AIE+B: Mean (SD)	xx (xx)	xx (xx)	xx (xx)
AIE vs AIE+B: Adjusted$^{\alpha}$ mean difference (95% CI)	xx (xx, xx)	xx (xx, xx)	xx (xx, xx)
WOMAC function: (0-68)			
AIE: Mean (SD)	xx (xx)	xx (xx)	xx (xx)
AIE+B: Mean (SD)	xx (xx)	xx (xx)	xx (xx)
AIE vs AIE+B: Adjusted$^{\alpha}$ mean difference (95% CI)	xx (xx, xx)	xx (xx, xx)	xx (xx, xx)
Last 7 days, knee pain during activity in the knee: (0-10)			
AIE: Mean (SD)	xx (xx)	xx (xx)	xx (xx)
AIE+B: Mean (SD)	xx (xx)	xx (xx)	xx (xx)
AIE vs AIE+B: Adjusted$^{\alpha}$ mean difference (95% CI)	xx (xx, xx)	xx (xx, xx)	xx (xx, xx)
Intermittent and constant pain (ICOAP): constant pain subscale: (0-100)			
AIE: Mean (SD)	xx (xx)	xx (xx)	xx (xx)
AIE+B: Mean (SD)	xx (xx)	xx (xx)	xx (xx)
AIE vs AIE+B: Adjusted$^{\alpha}$ mean difference (95% CI)	xx (xx, xx)	xx (xx, xx)	xx (xx, xx)
Intermittent and constant pain (ICOAP): Intermittent pain subscale: (0-100)			
AIE: Mean (SD)	xx (xx)	xx (xx)	xx (xx)
AIE+B: Mean (SD)	xx (xx)	xx (xx)	xx (xx)
AIE vs AIE+B: Adjusted$^{\alpha}$ mean difference (95% CI)	xx (xx, xx)	xx (xx, xx)	xx (xx, xx)
Intermittent and constant pain (ICOAP): total pain subscale: (0-100)			
AIE: Mean (SD)	xx (xx)	xx (xx)	xx (xx)

AIE+B: Mean (SD)	xx (xx)	xx (xx)	xx (xx)
AIE vs AIE+B: Adjusted[α] mean difference (95% CI)	xx (xx, xx)	xx (xx, xx)	xx (xx, xx)
Arthritis self-efficacy (SEE): (1-10)			
AIE: Mean (SD)	xx (xx)	xx (xx)	xx (xx)
AIE+B: Mean (SD)	xx (xx)	xx (xx)	xx (xx)
AIE vs AIE+B: Adjusted[α] mean difference (95% CI)	xx (xx, xx)	xx (xx, xx)	xx (xx, xx)
Physical activity (IPAQ-E): (MET minutes per week; 0 - 19278):			
AIE: Mean (SD)	xx (xx)	xx (xx)	xx (xx)
AIE+B: Mean (SD)	xx (xx)	xx (xx)	xx (xx)
AIE vs AIE+B: Adjusted[α] mean difference (95% CI)	xx (xx, xx)	xx (xx, xx)	xx (xx, xx)
Social participation (PROMIS): (27.5-64.2)			
AIE: Mean (SD)	xx (xx)	xx (xx)	xx (xx)
AIE+B: Mean (SD)	xx (xx)	xx (xx)	xx (xx)
AIE vs AIE+B: Adjusted[α] mean difference (95% CI)	xx (xx, xx)	xx (xx, xx)	xx (xx, xx)
OMERACT-OARSI responder criteria			
AIE: N (%)	xx (xx)	xx (xx)	xx (xx)
AIE+B: N (%)	xx (xx)	xx (xx)	xx (xx)
AIE vs AIE+B: Adjusted[α] odds ratio (95% CI)	xx (xx, xx)	xx (xx, xx)	xx (xx, xx)

[α] Adjusted for PROP-OA clinic site, predominant compartmental distribution, presence/absence of instability (buckling), age, sex, baseline anxiety, baseline depression, and baseline in the outcome of interest (except for the OMERACT-OARSI responder criteria). Results based on analysis population 1 unless otherwise stated. CI = confidence interval

12.3 Clinical effectiveness paper – supplementary tables (part 1: clinical effectiveness analysis of AIE compared to AIE+B)

Table 12.3.1: Reasons for ineligibility

**Ineligibility reasons on the telephone screen**	Total N(%)
Aged < 45 years	xx (xx)
Not a UK resident	xx (xx)
Unable to read/write English	xx (xx)
No access to a mobile phone that can receive text messages	xx (xx)
No knee pain	xx (xx)
Pain severity in the last 7 days <4 (index knee)	xx (xx)
Previous knee replacement	xx (xx)
Had cartilage implants in the last 12 months (index knee)	xx (xx)
Major injury or trauma to the index knee in the last 3 months	xx (xx)
Physiotherapy in the last 3 months (index knee)	xx (xx)
Injection in the last 3 months (index knee)	xx (xx)
Worn a knee brace in the last 3 months (index knee)	xx (xx)
On the waiting list for a hip or knee replacement in next 6-months	xx (xx)
Under regular follow-up with a rheumatologist	xx (xx)
Taking relevant medication for inflammatory arthritis	xx (xx)
Has fibromyalgia	xx (xx)
Has Parkinson's disease	xx (xx)
Pregnant or breast feeding	xx (xx)
Family member already in the study	xx (xx)
Unwilling to wear a knee brace	xx (xx)
Unwilling to attend study appointments	xx (xx)
Didn’t give consent to take part	xx (xx)
Reason for ineligibility unknown	xx (xx)
**Total**	**xxx**
**Ineligibility reasons at the clinical assessment**	Total N(%)
Has a red flag	xx (xx)
Has inflammatory/crystal arthritis	xx (xx)
Has a significant neurological disorder	xx (xx)
Vulnerable individual	xx (xx)
Fibromyalgia	xx (xx)
Previous major surgery to the index knee	xx (xx)
Autologous cartilage implantation in last 12-months (index knee)	xx (xx)
On waiting list for knee or hip replacement in the next 6-months	xx (xx)
Had physiotherapy in the last 3 months (index knee)	xx (xx)
Had injection in the last 3 months (index knee)	xx (xx)
Worn a brace in the last 3 months (index knee)	xx (xx)
Has a contra-indication to having new knee x-rays	xx (xx)
Knee brace contraindicated (superficial wounds, psoriasis, eczema, poor circulation, arterial insufficiency, severe varicosities, history of thrombophlebitis in either leg)	xx (xx)
Symptoms not attributable to knee osteoarthritis	xx (xx)
Fixed flexion deformity that prevents fitting of brace	xx (xx)
Brace size unavailable for leg circumference	xx (xx)
Does not want to take part	xx (xx)

**Total**	**xxx**

Figures are numbers (percentages in brackets). The first reason on the list above to apply is coded as the reason for ineligibility as multiple reasons for ineligibility can apply <<Add in a footnote to explain how many people had a repeat eligibility screen due to COVID>>

Table 12.3.2: Number randomised by recruitment method

	Randomised N = XXX
**Recruitment method**	
Physiotherapy	xx (xx)
GP (letter or consultation)	xx (xx)
Advertisement	xx (xx)
Social media	xx (xx)
Advertisement on a website	xx (xx)
Radio	xx (xx)
Advertisement on a bus	xx (xx)
Local poster/flyer	xx (xx)
Newspaper/magazine	xx (xx)
PROP OA study website	xx (xx)
Word of mouth	xx (xx)
Evergreen app	xx (xx)
Other	xx (xx)
Recruitment method missing	xx (xx)
Total	xx (xx)

Figures are numbers (percentages)

Table 12.3.3: Additional baseline characteristics: characteristics of knee problem by participant self-report and by plain x-ray of the participant's index knee

	All randomised participants N=XXX$^{\alpha}$	AIE N = XXX	AIE+B N = XXX
Last month, pain aching or stiffness in the knee			
No days	xx (xx)	xx (xx)	xx (xx)
Few days	xx (xx)	xx (xx)	xx (xx)
Some days	xx (xx)	xx (xx)	xx (xx)
Most days	xx (xx)	xx (xx)	xx (xx)
All days	xx (xx)	xx (xx)	xx (xx)
	Percentage denominator is participants reporting knee buckling (N= xx)	Percentage denominator is participants reporting knee buckling (N= xx)	Percentage denominator is participants reporting knee buckling (N= xx)
Knee buckling frequency last 3-months			
1 time	xx (xx)	xx (xx)	xx (xx)
2-5 times	xx (xx)	xx (xx)	xx (xx)
6-10 times	xx (xx)	xx (xx)	xx (xx)
11-24 times	xx (xx)	xx (xx)	xx (xx)
More than 24 times	xx (xx)	xx (xx)	xx (xx)
Don't know	xx (xx)	xx (xx)	xx (xx)
Fell and hit the floor/ground after knee buckling			
Yes	xx (xx)	xx (xx)	xx (xx)
No	xx (xx)	xx (xx)	xx (xx)
Don't know	xx (xx)	xx (xx)	xx (xx)
Activity partaken when knee buckled			
Walking	xx (xx)	xx (xx)	xx (xx)
Going up or down stairs	xx (xx)	xx (xx)	xx (xx)
Twisting or turning	xx (xx)	xx (xx)	xx (xx)
Other	xx (xx)	xx (xx)	xx (xx)
Don't know	xx (xx)	xx (xx)	xx (xx)

Figures are numbers (percentages in brackets). All data completed in reference to the knee to be treated. α = baseline questionnaire data is missing for XXX participants hence analysis based on XXX participants with data.

Table 12.3.4: Key baseline characteristics by method of recruitment

<<Table 12.2.1 will be copied, but stratified by method of recruitment (i.e. identification via screen of physiotherapy referrals, general practice consulters, self-referral from the community) rather than treatment arm>>

Table 12.3.5: Key baseline characteristics by loss to follow-up

<<Table 12.2.1 will be copied, but stratified by loss to follow-up at 3-month, 6-month and 12-month, rather than treatment arm>>

Table 12.3.6: Characteristics of participants at each stage of study recruitment

	Eligible on the Telephone screen (N =XXX)	Attended Clinical Assessment (N=XXX)	Eligible at Clinical Assessment (N=XXX)	Attended initial treatment visit (N=XXX)	Randomised (N=XXX)
Age: Mean (SD)	xx(xx)	xx(xx)	xx(xx)	xx(xx)	xx(xx)
Female sex	xx(xx)	xx(xx)	xx(xx)	xx(xx)	xx(xx)
Index of multiple deprivation (IMD) (1 - 32,844): Mean (SD)	xx(xx)	xx(xx)	xx(xx)	xx(xx)	xx(xx)
IMD Quintile					
1: IMD 1 to 6568	xx(xx)	xx(xx)	xx(xx)	xx(xx)	xx(xx)
2: IMD 6569 to 13137	xx(xx)	xx(xx)	xx(xx)	xx(xx)	xx(xx)
3: IMD 13138 to 19706	xx(xx)	xx(xx)	xx(xx)	xx(xx)	xx(xx)
4: IMD 19707 to 26275	xx(xx)	xx(xx)	xx(xx)	xx(xx)	xx(xx)
5: IMD 26276 to 32844	xx(xx)	xx(xx)	xx(xx)	xx(xx)	xx(xx)
**Clinical judgement** on the most severely affected compartment					
Medial TIB-FEM joint	α	xx(xx)	xx(xx)	xx(xx)	xx(xx)
Lateral TIB-FEM joint	α	xx(xx)	xx(xx)	xx(xx)	xx(xx)
Patellofemoral joint	α	xx(xx)	xx(xx)	xx(xx)	xx(xx)
No predominant compartment	α	xx(xx)	xx(xx)	xx(xx)	xx(xx)
Knee to be treated					
Left	α	α	α	xx(xx)	xx(xx)
Right	α	α	α	xx(xx)	xx(xx)
**Clinical judgement and x-ray findings** on the most severely affected compartment					
Medial TIB-FEM joint	α	α	α	xx(xx)	xx(xx)
Lateral TIB-FEM joint	α	α	α	xx(xx)	xx(xx)
Patellofemoral joint	α	α	α	xx(xx)	xx(xx)
No predominant compartment	α	α	α	xx(xx)	xx(xx)

Figures are numbers (percentages in brackets) unless otherwise stated. SD = Standard deviation. α = Data not collected for this group of participants

Table 12.3.7: Missing data rates for the primary outcome (KOOS-5)

	N(%) of missing data			
	Baseline<br>N=XX	3-months<br>N=XX	6-months<br>N=XX	12-months<br>N=XX
KOOS-5 (primary outcome) (0-100)	xx (xx)	xx (xx)	xx (xx)	xx (xx)
KOOS: pain (0-100)	xx (xx)	xx (xx)	xx (xx)	xx (xx)
KOOS: symptoms (0-100)	xx (xx)	xx (xx)	xx (xx)	xx (xx)
KOOS: Activities of daily living (0-100)	xx (xx)	xx (xx)	xx (xx)	xx (xx)
KOOS: Sport/recreation (0-100)	xx (xx)	xx (xx)	xx (xx)	xx (xx)
KOOS: Knee related quality of life (0-100)	xx (xx)	xx (xx)	xx (xx)	xx (xx)

Missing data rates for the secondary outcome measures will not be presented in tabular format but will be reported in the text of the paper with wording such as: "Missing data rates for the secondary outcomes was less than x% at all follow-up time-points".

Table 12.3.8: Missing data patterns for the primary outcome (KOOS-5)

Baseline	3-months	6-months	12-months	N (%)
Yes	Yes	Yes	Yes	x (x)
Yes	Yes	Yes	No	x (x)
Yes	Yes	No	No	x (x)
Etc				

<<The percentage of participants with 0, 1, 2, 3, and 4 data points with complete data will also be reported>>

Table 12.3.9: Protocol deviations

Deviation Number	Deviation	How many participants affected	Deviation occurred due to COVID pandemic	Deviation impacts on primary and secondary data collected at the time-point			Treatment Arm
				3-months	6-months	12-months	
1	xxxx	xx	xx	Y/N	Y/N	Y/N	xx
2	xxxx	xx	xx	Y/N	Y/N	Y/N	xx
etc	Participants do not meet our *a priori* definition of being treated per protocol (see section 2.3)	xx	xx	Y/N	Y/N	Y/N	xx

If this table is very long, we will add a footnote to say that only deviations that impact of primary and secondary data collection are listed.

Table 12.3.10: Exploratory subgroup analyses for the KOOS-5 primary outcome at 6-month follow-up

	Mean (SD) of KOOS-5	Interaction (95% CI)
**Categorical variables**		
**Predominant knee compartment based on clinical and x-ray findings**		
Medial tibiofemoral joint: N = X		
AIE	xx(xx)	0
AIE+B	xx(xx)	
Lateral tibiofemoral joint: N = X		
AIE	xx(xx)	xx (xx, xx)
AIE+B	xx(xx)	
Patellofemoral joint: N = X		
AIE	xx(xx)	xx (xx, xx)
AIE+B	xx(xx)	
No predominant Compartment: N = X		
AIE	xx(xx)	xx (xx, xx)
AIE+B	xx(xx)	
**Knee buckling at baseline**		
Yes: N = X		
AIE	xx(xx)	0
AIE+B	xx(xx)	
No/not sure: N =X		
AIE	xx(xx)	xx (xx, xx)
AIE+B	xx(xx)	
**Level of adherence at 6-month follow-up$^{\alpha}$**		
Never: N = X		
AIE	xx(xx)	0
AIE+B	xx(xx)	
Rarely: N = X		
AIE	xx(xx)	xx (xx, xx)
AIE+B	xx(xx)	
Sometimes: N = X		
AIE	xx(xx)	xx (xx, xx)
AIE+B	xx(xx)	
Often: N = X		
AIE	xx(xx)	xx (xx, xx)
AIE+B	xx(xx)	
All of the time: N = X		
AIE	xx(xx)	xx (xx, xx)
AIE+B	xx(xx)	
Don’t know: N = X		
AIE	xx(xx)	xx (xx, xx)
AIE+B	xx(xx)	
**Sex**		
Male: N= X		
AIE	xx(xx)	0

AIE+B	xx(xx)	
Female: N=X		
AIE	xx(xx)	xx (xx, xx)
AIE+B	xx(xx)	
**Ethnicity**		
White: N= X		
AIE	xx(xx)	0
AIE+B	xx(xx)	
Non-white: N=X		
AIE	xx(xx)	xx (xx, xx)
AIE+B	xx(xx)	
**Continuous variables at baseline**		
HADS anxiety score	N/A	xx (xx, xx)
HADS depression score	N/A	xx (xx, xx)
KOOS-5	N/A	xx (xx, xx)

SD = standard deviation, CI = confidence interval, N/A = not applicable, HADS = Hospital Anxiety and Depression Scale. α Defined at 6-month follow-up using the question: In the last 3-months, have you been following the advice and treatment you received from the physiotherapist as often as you were advised to? β Adjusted for PROP-OA clinic site, predominant compartmental distribution based on clinical and x-ray findings, presence/absence of instability (buckling), age, sex, baseline anxiety, baseline depression, baseline KOOS-5 score

12.4 Tables and figures for health economics paper

Table 12.4.1: Unit Costs applied for valuation of resource use

Health care resource	Unit cost (£)
**Interventions**	
**AIE+B**	
Knee brace (to be listed for each brace type)	XX.XX
Physiotherapist consultation (1 hour, dependent on grade)	XX.XX
Physiotherapist follow-up (30 minutes, dependent on grade)	XX.XX
X-ray	XX.XX
SMS prompts	XX.XX
Supporting patient material	XX.XX
Training of physiotherapists for brace fitting/use	XX.XX
**AIE**	
Physiotherapist consultation (40 minutes, dependent on grade)	XX.XX
Best primary care materials (written material**)**	XX.XX
**Primary care**	
General Practitioner: surgery consultation	XX.XX
General Practitioner: home consultation	XX.XX
Practice Nurse: Surgery consultation	XX.XX
District Nurse: Home visit	XX.XX
First contact practitioner	XX.XX
Other	XX.XX
Prescribed Medication	Patient-specific
**Hospital care contacts**	
Outpatient consultant visit	XX.XX
Physiotherapist	XX.XX
Orthopaedic surgeon	XX.XX
Osteopath	XX.XX
Rheumatologist	XX.XX
Occupational therapist	XX.XX
Other contacts as specified	XX.XX
Diagnostic tests: X-ray	XX.XX
Diagnostic tests: MRI scan	XX.XX
Diagnostic tests: other as specified	XX.XX
Injection (as specified)	XX.XX
Knee arthroscopy	XX.XX
Knee replacement	XX.XX
Interventions: other as specified	XX.XX

Private health care: as specified	XX.XX
Out-of-pocket treatments	Patient reported costs
Periods of work absence	Patient-specific

Table 12.4.2: Descriptive and incremental health outcomes over 12 months for the base-case analysis and the complete case analyses. Values are mean (SD) scores unless stated otherwise.

	AIE<br>n =xx	AIE+B<br>n =xx	Difference<br>(CI) (AIE+B minus AIE)
	Primary (Imputed) EQ-5D analysis		
Baseline EQ-5D	xx.xx	xx.xx	xx.xx
3-months EQ-5D	xx.xx	xx.xx	xx.xx
6-months EQ-5D	xx.xx	xx.xx	xx.xx
12-months EQ-5D	xx.xx	xx.xx	xx.xx
Unadjusted total QALYs	xx.xx	xx.xx	xx.xx
Adjusted total QALYs	-	-	
Complete-case analysis	n =	n=	
Unadjusted total QALYs	xx.xx	xx.xx	
Adjusted total QALYs.	xx.xx	xx.xx	

Table 12.4.3: Healthcare resource use and costs per patient by treatment group. Values are means (standard deviations) unless stated otherwise (illustrative – final table will be dependent on participant responses).

Resource category	Mean (SD)					
	Resource Use (units)				Cost (£)	
	n (%)	AIE<br>n = xx	n (%)	AIE+B<br>n = xx	AIE<br>n = xx	AIE+B<br>n = xx
Interventions						
AIE+B		XX.XX		XX.XX	XX.XX	XX.XX
AIE		XX.XX		XX.XX	XX.XX	XX.XX
Primary care						
GP		XX.XX		XX.XX	XX.XX	XX.XX
First contact practitioner		XX.XX		XX.XX	XX.XX	XX.XX
Practice nurse		XX.XX		XX.XX	XX.XX	XX.XX
District nurse		XX.XX		XX.XX	XX.XX	XX.XX
Prescribed Medication		XX.XX		XX.XX	XX.XX	XX.XX
Secondary care - NHS						
Outpatient		XX.XX		XX.XX	XX.XX	XX.XX
Physiotherapist		XX.XX		XX.XX	XX.XX	XX.XX
Visits other		XX.XX		XX.XX	XX.XX	XX.XX

Diagnostic tests	XX.XX	XX.XX	XX.XX	XX.XX
Hospital stay/surgery	XX.XX	XX.XX	XX.XX	XX.XX
Other interventions				
Secondary care – Private				
Outpatient consultant	XX.XX	XX.XX	XX.XX	XX.XX
Physiotherapist	XX.XX	XX.XX	XX.XX	XX.XX
Other visits	XX.XX	XX.XX	XX.XX	XX.XX
Out of pocket treatments	XX.XX	XX.XX	XX.XX	XX.XX
Time-off work	XX.XX	XX.XX	XX.XX	XX.XX

Table 12.4.4: Total costs and cost-effectiveness analysis results, by intervention arm

**Imputed analysis**	**AIE (£)**		**AIE+B (£)**
**Total NHS cost (base-case):**	xx.xx		xx.xx
Mean difference (95% CI)		xx.xx	
**Total Health care cost:**	xx.xx		xx.xx
Mean difference (95% CI)		xx.xx	
**Total Societal cost:**	xx.xx		xx.xx
Mean difference (95% CI)		xx.xx	

	Cost-effectiveness outcomes over 12 months			Probability SC is cost-effective at cost-effectiveness threshold of		
	Mean incremental costs (AIE+B minus AIE) (95% CI), £	Mean incremental QALYs (AIE+B minus AIE) (95% CI)	ICER £ per QALY gained	£20,000 per QALY	£30,000 per QALY	£50,000 per QALY
Base-case: NHS perspective	xx.xx	xx.xx		x.xx (x.xx)	x.xx (x.xx)	x.xx (x.xx)
**Sensitivity analysis 1:** Alternative perspectives						
Healthcare perspective	xx.xx	xx.xx		x.xx (x.xx)	x.xx (x.xx)	x.xx (x.xx)
Societal perspective	xx.xx	xx.xx		x.xx (x.xx)	x.xx (x.xx)	x.xx (x.xx)
**Sensitivity analysis 2:** Complete-case analysis						
NHS perspective	xx.xx	xx.xx				
**Sensitivity analyses:** Other (e.g. changing cost of brace)						
NHS perspective				x.xx (x.xx)	x.xx (x.xx)	x.xx (x.xx)

ICER = Incremental cost-effectiveness ratio. CI = confidence interval difference. NHS = National Health Service.

Figure 1: Cost-utility plane comparing AIE+B with AIE.

Figure 2: Cost-utility acceptability curve comparing AIE+B with AIE.

12.5 Clinical effectiveness paper – supplementary tables (part 2: patients' and physiotherapists' experience of AIE and AIE+B)

Table 12.5.1: Serious and unexpected adverse events

Date of adverse event onset (if known)	Date of report	Description	Serious adverse event (y/n)	Related to the intervention (y/n)	Treatment Arm
xx/xx/xx	xx/xx/xx	xx	xx	xx	xx
xx/xx/xx	xx/xx/xx	xx	xx	xx	xx
etc					

Table 12.5.2: Expected adverse events: physiotherapy follow-up case report form

Did the participant report, or have you observed any of the following over the site of the knee brace?	Participants allocated a knee brace with a follow-up visit N=XX
Skin redness	xx (xx)
Blistering	xx (xx)
Broken skin	xx (xx)
A marked increase in pain or swelling caused by the knee brace	xx (xx)
Sensation changes in the leg	xx (xx)
Severe skin soreness	xx (xx)
Other	xx (xx)

Figures are numbers (percentages in brackets).

Table 12.5.3: Expected adverse events: participant self-report

	**3-months**		**6-months**		**12-months**	
	AIE (N=XX)	AIE+B (N=XX)	AIE (N=XX)	AIE+B (N=XX)	AIE (N=XX)	AIE+B (N=XX)
Last X-months experienced any of the following in or around your knee						
Irritation/redness of skin	xx (xx)	xx (xx)	xx (xx)	xx (xx)	xx (xx)	xx (xx)
Blisters	xx (xx)	xx (xx)	xx (xx)	xx (xx)	xx (xx)	xx (xx)
New or abnormal symptoms	xx (xx)	xx (xx)	xx (xx)	xx (xx)	xx (xx)	xx (xx)
Increased swelling	xx (xx)	xx (xx)	xx (xx)	xx (xx)	xx (xx)	xx (xx)
Temporary increased soreness	xx (xx)	xx (xx)	xx (xx)	xx (xx)	xx (xx)	xx (xx)

Figures are numbers (percentages in brackets). X = 3 for the 3-month and 6-month follow-up; X= 6 for the 12-month follow-up

Table 12.5.4: Intervention acceptability at 3-month follow-up (from Sekhon et al., 2022)

	AIE (N=XX)	AIE+B (N=XX)
Acceptability of advice and treatment from the physiotherapist		
Completely unacceptable	xx(xx)	xx(xx)
Unacceptable	xx(xx)	xx(xx)
No opinion	xx(xx)	xx(xx)
Acceptable	xx(xx)	xx(xx)
Completely acceptable	xx(xx)	xx(xx)
Missing	xx(xx)	xx(xx)
Like or dislike the advice and treatment received from the physiotherapist		
Strongly dislike	xx(xx)	xx(xx)
Dislike	xx(xx)	xx(xx)
No opinion	xx(xx)	xx(xx)
Like	xx(xx)	xx(xx)
Strongly like	xx(xx)	xx(xx)
Missing	xx(xx)	xx(xx)
Effort to engage with treatment		
No effort at all	xx(xx)	xx(xx)
A little effort	xx(xx)	xx(xx)
No opinion	xx(xx)	xx(xx)
A lot of effort	xx(xx)	xx(xx)
Huge effort	xx(xx)	xx(xx)
Missing	xx(xx)	xx(xx)
There are moral or ethical consequences to engaging with the treatment and putting into practice the physiotherapists' advice		
Strongly disagree	xx(xx)	xx(xx)
Disagree	xx(xx)	xx(xx)
No opinion	xx(xx)	xx(xx)
Agree	xx(xx)	xx(xx)
Strongly agree	xx(xx)	xx(xx)
Missing	xx(xx)	xx(xx)
How fair (to all patients) is a system where all patients are offered the advice and treatment you received from the physiotherapist		
Very unfair	xx(xx)	xx(xx)
Unfair	xx(xx)	xx(xx)
No opinion	xx(xx)	xx(xx)
Fair	xx(xx)	xx(xx)
Very fair	xx(xx)	xx(xx)
Missing	xx(xx)	xx(xx)
Advice and treatment from the physiotherapist is likely to improve my knee problems in the long-term		
Strongly disagree	xx(xx)	xx(xx)
Disagree	xx(xx)	xx(xx)

No opinion	xx(xx)	xx(xx)
Agree	xx(xx)	xx(xx)
Strongly agree	xx(xx)	xx(xx)
Missing	xx(xx)	xx(xx)
It is clear how the advice and treatment from the physiotherapist would help me manage my knee problem in the long-term		
Strongly disagree	xx(xx)	xx(xx)
Disagree	xx(xx)	xx(xx)
No opinion	xx(xx)	xx(xx)
Agree	xx(xx)	xx(xx)
Strongly agree	xx(xx)	xx(xx)
Missing	xx(xx)	xx(xx)
Confidence to engage with the treatment and put into practice the advice from the physiotherapist in the long-term		
Very unconfident	xx(xx)	xx(xx)
Unconfident	xx(xx)	xx(xx)
No opinion	xx(xx)	xx(xx)
Confident	xx(xx)	xx(xx)
Very confident	xx(xx)	xx(xx)
Missing	xx(xx)	xx(xx)
Engaging with the treatment and putting into practice the physiotherapists' advice would interfere with my other priorities		
Strongly disagree	xx(xx)	xx(xx)
Disagree	xx(xx)	xx(xx)
No opinion	xx(xx)	xx(xx)
Agree	xx(xx)	xx(xx)
Strongly agree	xx(xx)	xx(xx)
Missing	xx(xx)	xx(xx)

Figures are numbers (percentages in brackets). Individual treatment acceptability questions are based on the theoretical framework of acceptability (TFA) (Sekhon et al. 2022)

Table 12.5.5: Adherence to the trial treatment (AIE or AIE+B)

	3-months		6-months		12-months	
	AIE (N=X)	AIE+B (N=X)	AIE (N=X)	AIE+B (N=X)	AIE (N=X)	AIE+B (N=X)
Last X-months, followed advice and treatment from physiotherapist as often as advised						
Never	xx (xx)	xx (xx)	xx (xx)	xx (xx)	xx (xx)	xx (xx)
Rarely	xx (xx)	xx (xx)	xx (xx)	xx (xx)	xx (xx)	xx (xx)
Sometimes	xx (xx)	xx (xx)	xx (xx)	xx (xx)	xx (xx)	xx (xx)
Often	xx (xx)	xx (xx)	xx (xx)	xx (xx)	xx (xx)	xx (xx)
All of the time	xx (xx)	xx (xx)	xx (xx)	xx (xx)	xx (xx)	xx (xx)
Don’t know	xx (xx)	xx (xx)	xx (xx)	xx (xx)	xx (xx)	xx (xx)

Footnote: X = 3 for the 3-month and 6-month follow-up; X= 6 for the 12-month follow-up

Table 12.5.6: Brace use in the AIE+B arm only

	**3-months** (N=X)	**6-months** (N=X)	**12-months** (N=X)
Past 7 days, number of days worn a knee brace for more than one hour			
0	xx (xx)	xx (xx)	xx (xx)
1	xx (xx)	xx (xx)	xx (xx)
2	xx (xx)	xx (xx)	xx (xx)
3	xx (xx)	xx (xx)	xx (xx)
4	xx (xx)	xx (xx)	xx (xx)
5	xx (xx)	xx (xx)	xx (xx)
6	xx (xx)	xx (xx)	xx (xx)
7	xx (xx)	xx (xx)	xx (xx)
Mean (SD)	xx (xx)	xx (xx)	xx (xx)
Past 7 days, number of hours per day worn a knee brace			
Mean (SD)	xx (xx)	xx (xx)	xx (xx)
Past 7 days, total time spent wearing a knee brace			
Mean (SD)	xx (xx)	xx (xx)	xx (xx)
Reasons for non-brace wear			
Problems with brace fit	xx (xx)	xx (xx)	xx (xx)
Brace look and feeling self-conscious	xx (xx)	xx (xx)	xx (xx)
Can't wear some types of clothing	xx (xx)	xx (xx)	xx (xx)
Don't know how to put it on	xx (xx)	xx (xx)	xx (xx)
Knee symptoms improved	xx (xx)	xx (xx)	xx (xx)
Don't think it's doing any good	xx (xx)	xx (xx)	xx (xx)
Increased pain or other symptoms	xx (xx)	xx (xx)	xx (xx)
Too much of a hassle putting it on/off	xx (xx)	xx (xx)	xx (xx)
Uncomfortable to wear	xx (xx)	xx (xx)	xx (xx)
Lost/mislaid the brace	xx (xx)	xx (xx)	xx (xx)
Brace is damaged or worn	xx (xx)	xx (xx)	xx (xx)
Too busy/not enough time	xx (xx)	xx (xx)	xx (xx)
No longer doing the activities that require the brace	xx (xx)	xx (xx)	xx (xx)
Don't want to become reliant on it	xx (xx)	xx (xx)	xx (xx)
Forget to wear it	xx (xx)	xx (xx)	xx (xx)
Wearing it has not become a habit	xx (xx)	xx (xx)	xx (xx)
Other$^{\alpha}$	xx (xx)	xx (xx)	xx (xx)

Figures are numbers (percentages in brackets) unless otherwise stated. SD = standard deviation. α includes….

Table 12.5.7: Treatment delivery – AIE

Treatment delivered	Participants in the AIE arm<br>N=XX
Provided verbal advice and education about osteoarthritis	XX (XXX)
Provided verbal advice about things to try at home to help with symptoms	XX (XXX)
Provided the osteoarthritis guidebook	XX (XXX)
Prescribed a knee exercise programme	XX (XXX)
Run through/demonstrate the exercise programme	XX (XXX)
Provide the written exercise programme	XX (XXX)
Other	XX (XXX)

Figures are numbers (percentages in brackets).

Table 12.5.8: Treatment delivery – AIE+B: initial treatment session

	Participants allocated a knee brace with an initial treatment visit N=xx (unless otherwise stated)
When the brace was issued did you:	
Contour brace hinges	xx (xx)
(denominator = those allocated a Ossur formfit knee hinged brace: N = XX)	
Cut brace straps	xx (xx)
Adjust brace 'force'	xx (xx)
(denominator = those allocated an unloader brace only: N = XX)	
Practice walking with the brace on	xx (xx)
Practice stairs with the brace on	xx (xx)
Get the participant to demonstrate taking the brace on and off	xx (xx)
Provide verbal advice on how the brace works and how to care for it	xx (xx)
Provide verbal advice on how often to wear the brace initially and how to build up use over time	xx (xx)
Provide the written brace information leaflet	xx (xx)
Address specific problems/concerns raised by participants	xx (xx$^{\alpha}$)
On first trying the brace on in clinic, did the participant report:	
Marked reduction in knee pain	xx (xx)
Marked increase in knee pain	xx (xx)
No marked change in knee pain	xx (xx)
How satisfied were you with your brace fitting for the participant	
Not at all satisfied	xx (xx)
Somewhat satisfied	xx (xx)
Moderately satisfied	xx (xx)
Very satisfied	xx (xx)
Extremely satisfied	xx (xx)
How many affirmations did you employ	

None	xx (xx)
1-2	xx (xx)
3-4	xx (xx)
5 or more	xx (xx)
How often did you employ reflective listening	
None of the time	xx (xx)
Reflected every statement made by patient	xx (xx)
For every question I asked, I gave 1-2 reflections	xx (xx)
Reflected occasionally when I felt it was necessary	xx (xx)
How often did you ask a patient an open-ended question	
None of the time	xx (xx)
For every one open ended question I asked one closed question	xx (xx)
For every two open ended questions I asked one closed question	xx (xx)
For every three open ended questions I asked one closed question	xx (xx)
How often did you elicit change talk from patients	
I did not hear any change talk	xx (xx)
I heard change talk infrequently	xx (xx)
I heard change talk sometimes	xx (xx)
I heard change talk often	xx (xx)
How often did you use summaries	
I did not use summaries	xx (xx)
I used summaries only at the beginning and end of the session	xx (xx)
I used summaries only when transitioning to another topic and at the beginning and end of the session	xx (xx)
I used summaries only when transitioning to another topic, at the beginning and end of the session, and at times when I wanted to ensure that I was understanding things correctly	xx (xx)
Did you provide a knee brace diary	xx (xx)

Figures are numbers (percentages in brackets). α = X participants did not raise and specific problems or concerns to be addressed

Table 12.5.9: Treatment delivery – AIE+B: follow-up treatment session

	Participants allocated a knee brace with a follow-up visit N=xx (unless otherwise stated)
How often, and for how long, the participant had worn a knee brace was discussed	xx (xx)
Knee brace diary was reviewed	xx (xx)
(denominator = participants given a brace diary at the initial treatment visit: N = xx)	
Knee brace diary was completed…	
(denominator = participants whose brace diary was reviewed: N = xx)	
Not at all	xx (xx)
On a few occasions	xx (xx)
Partially	xx (xx)
Very well (omissions on a few occasions only)	xx (xx)
Did you do any of the following:	
Adjust brace fit	xx (xx)
Practice walking with the brace on	xx (xx)
Practice stairs with the brace on	xx (xx)
Get the participant to demonstrate taking the brace on and off	xx (xx)
Provide verbal advice on how the brace works and how to care for it	xx (xx)
Provide verbal advice on how often to wear the brace	xx (xx)
Provide the written brace information leaflet	xx (xx)
Address specific problems/concerns raised by participants	xx (xx)
How many affirmations did you employ	
None	xx (xx)
1-2	xx (xx)
3-4	xx (xx)
5 or more	xx (xx)
How often did you employ reflective listening	
None of the time	xx (xx)
Reflected every statement made by patient	xx (xx)
For every question I asked, I gave 1-2 reflections	xx (xx)
Reflected occasionally when I felt it was necessary	xx (xx)

How often did you ask a patient an open-ended question	
None of the time	xx (xx)
For every one open ended question I asked one closed question	xx (xx)
For every two open ended questions I asked one closed question	xx (xx)
For every three open ended questions I asked one closed question	xx (xx)
How often did you elicit change talk from patients	
I did not hear any change talk	xx (xx)
I heard change talk infrequently	xx (xx)
I heard change talk sometimes	xx (xx)
I heard change talk often	xx (xx)
How often did you use summaries	
I did not use summaries	xx (xx)
I used summaries only at the beginning and end of the session	xx (xx)
I used summaries only when transitioning to another topic and at the beginning and end of the session	xx (xx)
I used summaries only when transitioning to another topic, at the beginning and end of the session, and at times when I wanted to ensure that I was understanding things correctly	xx (xx)

Figures are numbers (percentages in brackets)

Table 12.5.10: Clinical assessment and x-ray findings on the most severely affected compartment in the knee to be treated.

	All randomised participants N=XX
**Clinical Assessment**	
Medial tibiofemoral joint	xx (xx)
Lateral tibiofemoral joint	xx (xx)
Patellofemoral joint	xx (xx)
No predominant compartment	xx (xx)
**X-ray**	
No/minimal radiographic OA	xx (xx)
Patellofemoral joint	xx (xx)
Medial tibiofemoral joint	xx (xx)
Lateral tibiofemoral joint	xx (xx)
Mixed radiographic OA - no predominant compartment	xx (xx)
**Combined judgement: clinical & x-ray**	
Medial tibiofemoral joint	xx (xx)
Lateral tibiofemoral joint	xx (xx)
Patellofemoral joint	xx (xx)
No predominant compartment	xx (xx)

Figures are numbers (percentages in brackets)

Table 12.5.11: Clinical judgement on the most severely affected compartment in the knee to be treated: comparing clinical judgement alone, with clinical judgement and x-ray findings combined

	**Clinical judgement combined with x-ray findings**				
**Clinical judgement alone**	Medial tibiofemoral joint	Lateral tibiofemoral joint	Patellofemoral joint	No predominant compartment	**Total**
Medial tibiofemoral joint	X	X	X	X	XX (XX)
Lateral tibiofemoral joint	X	X	X	X	XX (XX)
Patellofemoral joint	X	X	X	X	XX (XX)
No predominant compartment	X	X	X	X	XX (XX)
**Total**	X	X	X	X	XX (XX)
Agreement	XX				
Kappa$^{\alpha}$ (95% confidence interval)	XX (XX, XX)				

Figures are numbers (percentages in brackets). $^{\alpha}$ Kappa statistic is unweighted and unadjusted for prevalence and bias

Table 12.5.12: Comparing brace allocation based on clinical judgement alone with clinical judgement combined with x-ray findings (N=XXX)

	Clinical judgement and x-ray findings				
**Clinical judgment alone**	Medial unloader N=XX	Lateral unloader N=XX	Patellofemoral brace N=XX	Neutral stabilising brace N=XX	Total
Medial unloader	XX	XX	XX	XX	XX (XX)
Lateral unloader	XX	XX	XX	XX	XX (XX)
Patellofemoral brace	XX	XX	XX	XX	XX (XX)
Neutral stabilising brace	XX	XX	XX	XX	XX (XX)
Medial & Neutral	XX	XX	XX	XX	XX (XX)
Patellofemoral & Neutral	XX	XX	XX	XX	XX (XX)
Etc	XX	XX	XX	XX	XX (XX)
Total	XX (XX)	XX (XX)	XX (XX)	XX (XX)	XX (XX)
**Based on data where a single brace type was selected at both time points**					
Agreement			XX (XX)		
Kappa$^{\alpha}$ (95% confidence interval)			XX (XX, XX)		
**Based on all data where at least one brace type was selected at both time points**					
Agreement			XX (XX)		
Kappa$^{\alpha}$ (95% confidence interval)			XX (XX, XX)		

Figures are numbers (percentages in brackets) unless otherwise stated. $^{\alpha}$ Kappa statistic is unweighted and unadjusted for prevalence and bias

Table 12.5.13: Physiotherapists' confidence in judging the most severely affected compartment in the knee to be treated

	All randomised participants N=XX
**Clinical assessment alone**	
Not at all confident	xx (xx)
Somewhat confident	xx (xx)
Moderately confident	xx (xx)
Very confident	xx (xx)
Extremely confident	xx (xx)
**X-ray alone**	
Not at all confident	xx (xx)
Somewhat confident	xx (xx)
Moderately confident	xx (xx)
Very confident	xx (xx)
Extremely confident	xx (xx)
**Combined clinical assessment and x-ray**	
Not at all confident	xx (xx)
Somewhat confident	xx (xx)
Moderately confident	xx (xx)
Very confident	xx (xx)
Extremely confident	xx (xx)

Figures are numbers (percentages in brackets).

Table 12.5.14: Details of participants that changed the brace type they were allocated to after trial randomisation

Participant number$^{\alpha}$	Brace allocated prior to randomisation	Brace allocated post randomisation	Reason for the change in brace allocation
1			
2			
3			
4			
Etc.			

$^{\alpha}$ note that this is a sequential number only and not the participants' ID number in the trial

Supplementary figures

1. Graph of the mean (and associated 95% confidence intervals) for the total time spent wearing the brace in the last 7 days for each occasion of SMS text data collection.

2. Graph of the proportion (and associated 95% confidence intervals) of those reporting they had worn the brace for the minimal time (as defined in Table 4.1.1) for each occasion of SMS text data collection.

13 Appendices

13.1 Data coding rules applied prior to analyses

Table 13.1.1: Data coding rules applied prior to analyses

Participant Study Identification Number	Data source and question number	Issue	Principle Applied to the data	Action Required
xx	xx	xx	xx	xx
xx	xx	xx	xx	xx
xx	xx	xx	xx	xx
xx	xx	xx	xx	xx
xx	xx	xx	xx	xx
xx	xx	xx	xx	xx

Note that the KOOS, the ICOAP, IPAQ-E, Arthritis Self-efficacy (ASE-8), and EQ-5D-5L have specific instructions as part of the tool around how data are coded at the item level (prior to creation of the outcome measure of interest), which will need to be observed when completing the table above. We will use the references in Table 4.1.1 to access this information and to apply the rule as appropriate to the data where a coding decision is needed.

13.2 Pre-planned adaptations to the imputation strategy

Numerical issues, failure, and breakdown of the multiple imputation algorithm can arise, particularly when there are many variables to include in the imputation model (Nguyen et al. 2021). If this does arise, we plan to use the strategy below (sequentially) to explore how the imputation model can be adapted to ensure that it can be applied to the data.

13.2.1 Perfect prediction

Perfect prediction can arise when multiple categorical variables are included in the imputation model. This would be addressed by adding the STATA "augment" option to the imputation model – a procedure that works by adding in additional "pseudo-observations" to prevent the outcome being perfectly predicted (Nguyen et al. 2021).

13.2.2 Ordinal variables

Ordinal variables can be challenging to include in an imputation model due to the number of categories they contain. If, after inspection of the imputation model, it appears that the reason why the imputation model will not run is due to the inclusion of too many ordinal variables, we will use the STATA "ascontinuous" option for the ordinal variables. This imputes the ordinal outcomes using ordinal regression, but, when these outcomes are included as predictor variables in the imputation model for other outcomes, they are assumed to be continuous variables, rather than categorical, to reduce the number of degrees of freedom in the imputation model (StataCorp. 2022). We will only use this approach for ordinal outcomes that are measured using a relatively large number of the response categories (>= 4).

13.2.3 Number of nearest neighbours (k) in the predictive mean matching (PMM) models

Kleinke 2018, highlight that there is a trade-off when considering the number of nearest neighbours (k) to include in the PMM model: if k is too small a single participant's data could be repeatedly chosen as a donor in the imputation model, which would underestimate model standard errors, whereas if k is too large might results in inadequate donors and implausible imputations, hence biased inferences.

We have used the recommendation by Morris et al. 2014 to set the value of k in the imputation model to be 10. If this decision means that the imputation model breaks down when we fit it to our data, we will re-run the imputation model, firstly with k=5 and then secondly with k = 15 to see if these changes enable the imputation model to run in our data. We will try K=5, before K=15, as the former is preferred default value for K used in the alternative statistical software packages of SAS and R (Kleinke 2018).

13.2.4 Collinearity

We have chosen to include all primary and secondary outcome measures in our imputation model to reduce bias. However, in doing so, this could lead to breakdown of the model as some of the variables are direct transformations of other variables in the model so will be highly correlated (e.g. the KOOS-5 is a direct transformation of the subscale scores it is derived from). If the model does not run for this reason, we would exclude the derived variables from the imputation model and use the "mi passive" procedure in STATA to derive these measures after the imputation had been performed instead. The advantage of this approach is that the derived variables will always be consistent with the subscale scores (e.g. if a participant scored 0 for all KOOS subscales it would guarantee that the imputed KOOS-5 total score would be 0), which can't always be assumed under the primary approach. However, this was not chosen as the primary approach as reducing bias in treatment effect estimates was considered a greater priority.

13.2.5 Re-coding of the "Patient global rating of change" question

A key role for the "Patient global rating of change" question in this study is to facilitate the scoring of the OMERACT-OARSI responder criteria. The "Patient global rating of change" question is measured on a 6-point Likert scale in the data, however, to score the OMERACT-OARSI responder criteria, all that is required is to know whether the participant's symptoms have improved, not changed, or deteriorated. Given this, we will consider whether to reduce the number of categories for this measure by merging some data categories before the data are imputed. We will try to minimise the amount of category merging that is required, to avoid loss of information, so aim to firstly explore whether merge option 1 is successful before considering merge option 2 below.

Merging option 1: Completely better and Much better/Better/No change/Worse/Much worse

Merge option 2 if merge option 1 does not run: Completely better, much better, Better/No change/Worse and much worse

13.2.6 Dropping variables from the imputation model

It is recognised that we will have many variables to include as predictors in the imputation model. If, after the strategies described above have been employed, the imputation model still does not converge we will consider dropping some of the variables from the model, considered in the order below:

1. SMS text data on adherence at all time-points excluding our three key time-points of interest (3-, 6- and 12-months post-randomisation)
2. Drop all SMS text data from the model (we have focussed on the SMS text data as we know from our internal pilot study that this data is less complete than from the self-reported questionnaire data)
3. Drop the WOMAC scores for pain, stiffness and function at all time-points. The WOMAC scores are likely to be highly correlated with the KOOS-5 and KOOS-4 data (as they are derived from the same questions) so may make the imputation model not converge. The WOMAC was included to ensure this data was available for future meta-analyses so is not considered a key secondary outcome for this study.

13.2.7 Adapting the imputation model

We have chosen to use MICE as our imputation method, however, other imputation models exist, such as Multi-Variate Normal Imputation (MVNI), which could be used as an alternative approach (Nguyen et al. 2021). Therefore, if our MICE imputation model is unsuccessful, we will explore changing the imputation method to MVNI, to see if we can successfully impute the data using this method. In addition, as our data are in a repeated measures format, we would also consider whether a fully conditional specification (FCS) two-fold imputation model would be appropriate for our data. This procedure offers greater flexibility to the full imputation approach, as it includes only a subset of data collected within a pre-specified time-window in the imputation model, reducing the number of variables in the imputation model, and making it less likely to breakdown in the data (e.g. a time window of one would include in the imputation model only data collected at time t, t+1 and t-1, where applicable) (Huque et al. 2018).
